# Novel benzofuran-furan-pyrano[2,3-*c*]pyrazole hybrids as potential anticancer agents: design, synthesis, *in silico* profiling, and multi-mechanistic biological evaluation

**DOI:** 10.1039/d6ra05827b

**Published:** 2026-08-28

**Authors:** Elena Daniela Hutanu, Bassam A. Najri, Amine Hafis Abdelsalam, Katia Mohand Saidi, Sevki Arslan, Arif Kivrak

**Affiliations:** a Department of Biology, Faculty of Science, Pamukkale University Denizli 20160 Turkey; b Department of Chemistry, Faculty of Science, Eskisehir Osmangazi University Eskisehir 26040 Turkey; c Advanced Organic Synthesis Laboratory, Technology and Innovation Center, Eskisehir Osmangazi University Eskisehir 26040 Turkey; d Pharmaceutical Chemistry Team, Laboratory of Phytochemistry and Pharmacology, University of Mohamed Seddik Ben Yahia-Jijel Ouled Aissa, BP 98 Jijel 18000 Algeria

## Abstract

The development of anticancer agents capable of affecting multiple cancer-related cellular processes remains an important strategy in cancer drug discovery. In this study, a novel series of benzofuran-furan-pyrano[2,3-*c*]pyrazole hybrids was synthesized and comprehensively investigated through *in vitro* biological assays and *in silico* analyses. The synthesized compounds exhibited concentration-dependent cytotoxicity against LNCaP, MDA-MB-231, A549, and Caco-2 cancer cells, with EC_50_ values ranging from 20.49 to 274.37 µM while displaying lower toxicity toward non-malignant HUVEC cells. Among the synthesized derivatives, compounds 4j, 4h, and 4d showed the strongest antiproliferative activities and significantly suppressed cancer cell migration, invasion, and colony formation. Mechanistic studies demonstrated that the active hybrids induced G1/S phase cell cycle arrest, increased intracellular reactive oxygen species (ROS) production, disrupted mitochondrial membrane potential, and promoted apoptotic cell death. These findings were further supported by quantitative real-time PCR, which revealed downregulation of BCL2 together with upregulation of BAX, CASP3, CASP8, CASP9, and TP53, indicating activation of both intrinsic and extrinsic apoptotic pathways. *In silico* analysis of absorption, distribution, metabolism, excretion, and toxicity (ADMET) predicted favorable drug-likeness and pharmacokinetic properties for the synthesized hybrids. Molecular docking suggested potential interactions with CDK2, the androgen receptor, VEGFR-2, and Bcl-2, with compound 4f exhibiting the most favorable overall docking profile. Collectively, these findings demonstrate that benzofuran-furan-pyrano[2,3-*c*]pyrazole hybrids possess promising multifaceted *in vitro* anticancer activity by inhibiting cancer cell proliferation, migration, invasion, and clonogenicity while inducing ROS-mediated mitochondrial apoptosis, highlighting these hybrid scaffolds as attractive candidates for further anticancer drug development.

## Abstract 2

The development of anticancer agents capable of affecting multiple cancer-related cellular processes remains an important strategy in cancer drug discovery.

## Graphical abstract

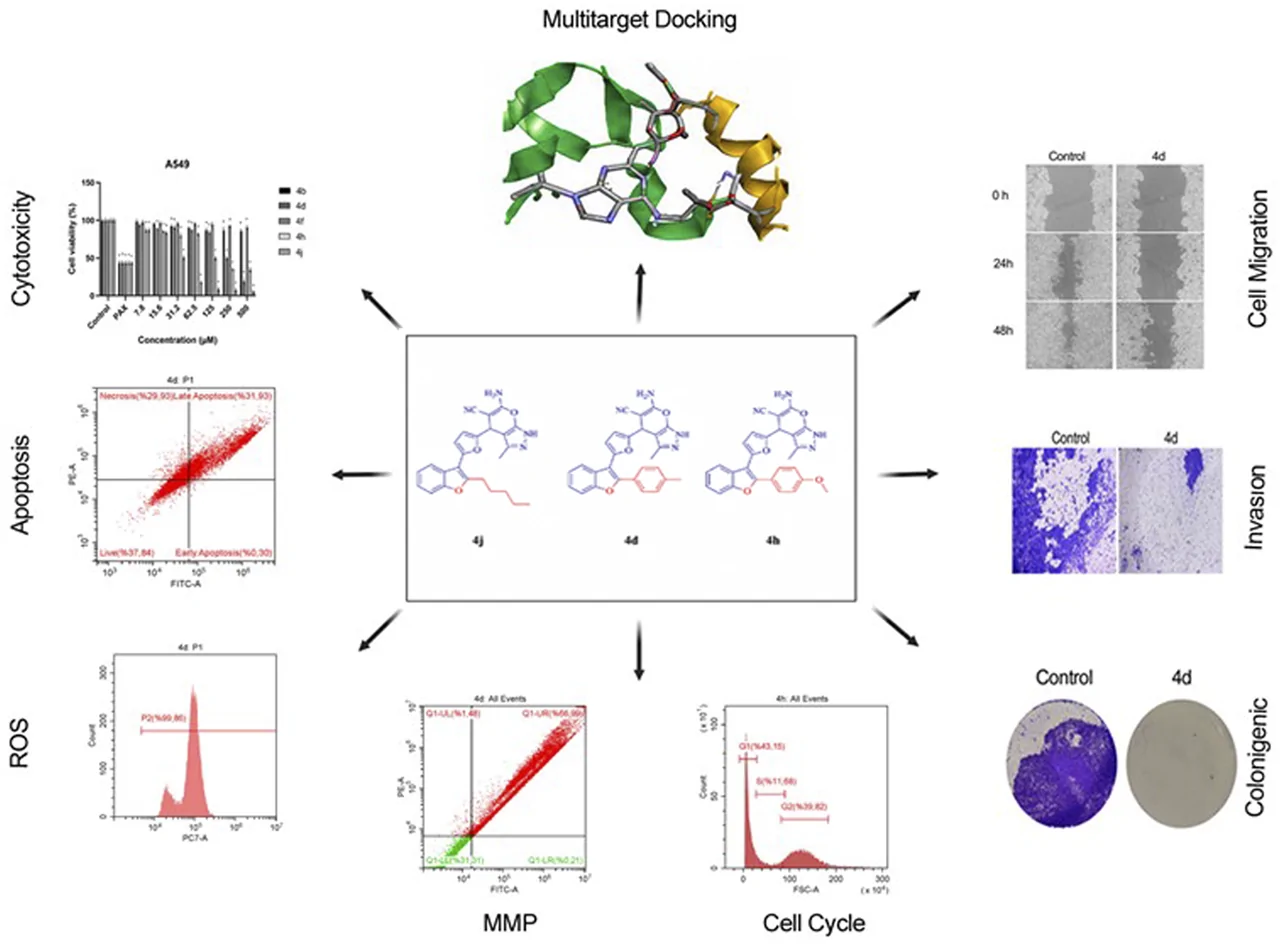

## 1. Introduction

Cancer remains one of the leading causes of morbidity and mortality worldwide and continues to represent a major public health burden despite substantial advances in diagnosis and treatment. Although surgery, radiotherapy, chemotherapy, and targeted therapies have significantly improved clinical outcomes for several malignancies, treatment efficacy is frequently compromised by severe systemic toxicity, acquired drug resistance, and tumor recurrence. Therefore, the development of novel anticancer agents with improved selectivity, enhanced therapeutic efficacy, and reduced adverse effects remains an important objective in modern medicinal chemistry.^1,2^

Tumor heterogeneity is a major obstacle to successful cancer therapy because genetically and phenotypically distinct cell populations within the same tumor can activate alternative signaling pathways and escape therapeutic intervention. Consequently, increasing attention has been directed toward multitarget therapeutic strategies capable of simultaneously modulating multiple cancer-related pathways. Among these approaches, molecular hybridization has emerged as an attractive drug design strategy by integrating two or more biologically active pharmacophores into a single molecular framework to enhance efficacy while minimizing resistance and toxicity.^3,4^

Heterocyclic compounds constitute one of the most important classes of bioactive molecules in medicinal chemistry and are present in more than 60% of currently marketed drugs. Their structural diversity and favorable physicochemical properties allow efficient interactions with numerous biological targets, making them indispensable scaffolds for the development of novel therapeutic agents.^2,5,6^ Among these privileged structures, benzofuran derivatives have attracted considerable interest because of their diverse pharmacological activities, including anticancer, antimicrobial, antioxidant, anti-inflammatory, and antiviral effects. Numerous studies have demonstrated that benzofuran derivatives inhibit cancer cell proliferation by inducing cell cycle arrest, promoting mitochondria-mediated apoptosis, increasing intracellular reactive oxygen species (ROS), and modulating multiple oncogenic signaling pathways.^1,7,8^

Similarly, pyran-containing heterocycles are recognized as valuable pharmacophores due to their broad spectrum of biological activities. Pyran derivatives regulate key signaling pathways, including PI3K/Akt, MAPK, and NF-κB, thereby influencing cell proliferation, apoptosis, oxidative stress, and cell cycle progression. Their structural versatility further supports their widespread application in anticancer drug discovery.^9–11^ Although pyrazole derivatives are relatively uncommon in nature, they have emerged as one of the most extensively investigated heterocyclic scaffolds owing to their remarkable antiproliferative, anti-inflammatory, and antimicrobial activities. Synthetic pyrazole-containing molecules have demonstrated significant cytotoxic activity against a variety of human cancer cell lines through modulation of kinase-mediated signaling, apoptosis, and oxidative stress pathways, making them attractive building blocks for hybrid molecule design.^9,11,12^

Previous studies have reported anticancer activity for benzofuran-pyrazole and pyrano[2,3-*c*]pyrazole systems. Benzofuropyrazole derivatives inhibited tumor cell growth in A549 and K562 cells, while pyrazole-based benzofuran analogues exhibited antiproliferative activity against A549, HT-29, and MCF-7 cells and were associated with mitochondrial dysfunction and apoptosis.^13,14^ Pyrano[2,3-*c*]pyrazole derivatives have also been developed as RalA inhibitors against hepatocellular carcinoma and as AKT2/PKBβ inhibitors with anti-glioma activity.^15,16^ In contrast to these previously reported scaffolds, the present compounds integrate benzofuran, furan, and pyrano[2,3-*c*]pyrazole motifs within a single molecular framework. This structural combination was designed to combine the reported pharmacological features of these heterocyclic scaffolds and to explore their effects on multiple cancer-related cellular processes.

In the present study, a novel series of benzofuran-furan-pyrano[2,3-*c*]pyrazole hybrids was rationally designed and synthesized to investigate the anticancer potential of integrating these privileged heterocyclic pharmacophores within a single molecular framework. The synthesized compounds were structurally characterized by ^1^H/^13^C nuclear magnetic resonance (NMR) spectroscopy and high-resolution mass spectrometry (HRMS), and their drug-likeness was evaluated according to Lipinski's Rule of Five. Their anticancer potential was comprehensively investigated through *in vitro* cytotoxicity, apoptosis, cell cycle, reactive oxygen species (ROS), mitochondrial membrane potential, migration, invasion, and clonogenic assays using multiple human cancer cell lines and non-malignant endothelial cells. In addition, molecular docking and ADMET analyses were performed to elucidate their potential molecular interactions and pharmacokinetic properties. This study provides a comprehensive biological and computational evaluation of benzofuran-furan-pyrano[2,3-*c*]pyrazole hybrids and highlights this hybrid scaffold as a promising candidate for further anticancer drug development.

## 2. Experimental section

### 2.1. General information

All synthesized compounds (1b, 1d, 1f, 1h and 1j, 2b, 2d, 2f, 2h and 2j, 3b, 3d, 3f, 3h and 3j, and 4b, 4d, 4f, 4h and 4j) were characterized by ^1^H and ^13^C NMR spectroscopy. The spectra were recorded on a 500 MHz NMR spectrometer for ^1^H NMR and 125 MHz for ^13^C NMR, using deuterated chloroform (CDCl_3_) as solvent and trimethylsilane (TMS) as the internal reference. Chemical shifts (*δ*) are expressed in ppm and coupling constants (*J*) in Hz. The following abbreviations are used for signal multiplicities: s = singlet, d = doublet, t = triplet, q = quartet, p = pentet, and m = multiplet. High-resolution mass spectra (HRMS) of the final compounds (4b, 4d, 4f, 4h and 4j) were recorded using an Agilent 6530 B LC-Q-TOF mass spectrometer equipped with an electrospray ionization source operating in positive-ion mode (ESI^+^). The HRMS data are reported as mass-to-charge ratios (*m*/*z*) for the protonated molecular ions [M + H]^+^. Reaction progress was monitored by analytical thin layer chromatography (TLC) on silica gel 60 F254 plates (Merck), and compounds were detected under ultraviolet (UV) light (254 nm). Purification by column chromatography was carried out on silica gel (230–400 mesh, CDH Fine Chemicals).

### 2.2. Synthesis of benzofuran-furan-pyrano[2,3-*c*]pyrazole hybrids (4b, 4d, 4f, 4h and 4j)

The target benzofuran-furan-pyrano[2,3-*c*]pyrazole hybrids (4b, 4d, 4f, 4h and 4j) were synthesized *via* a four-step reaction pathway, as depicted in Scheme S1. The complete compound series comprised benzene-containing derivatives (4a, 4c, 4e, 4g and 4i) and furan-containing derivatives (4b, 4d, 4f, 4h and 4j); the present study focuses exclusively on the furan-containing subgroup. The preparation of the intermediates (1b, 1d, 1f, 1h and 1j) (2b, 2d, 2f, 2h and 2j) and (3b, 3d, 3f, 3h and 3j) was carried out according to the procedure reported by Najri *et al.*,^17–21^ and the corresponding synthetic details are provided in the SI.

For the synthesis of compounds (4b, 4d, 4f, 4h and 4j), the appropriate benzofuran-furan aldehyde derivative (3b, 3d, 3f, 3h and 3j) (1.0 equiv.) was dissolved in ethanol (EtOH), followed by the addition of malononitrile (2.0 equiv.) and 3-methyl-1*H*-pyrazol-5-ol (1.5 equiv.). Potassium carbonate (K_2_CO_3_, 10 mol%) was then added, and the reaction mixture was stirred at room temperature. Rapid formation of a solid product was observed. Reaction progress was monitored by TLC, and after 2 min the spot corresponding to the starting benzofuran-furan aldehyde was no longer detectable. Stirring was immediately stopped, and the precipitated product was isolated by filtration and washed thoroughly with cold ethanol. No additional chemical quenching step was required because the product was directly separated from the reaction medium by filtration. The isolated solid was dried and subsequently recrystallized from ethanol to afford the desired benzofuran-furan-pyrano[2,3-*c*]pyrazole hybrids (4b, 4d, 4f, 4h and 4j).

All final compounds were confirmed by nuclear magnetic resonance (NMR) spectroscopy and the corresponding data are provided in the SI.

#### 2.2.1. 6-Amino-3-methyl-4-(5-(2-phenylbenzofuran-3-yl)furan-2-yl)-1,4-dihydropyrano[2,3-*c*]pyrazole-5-carbonitrile (4b)

Orange solid; 96% yield; ^1^H NMR (500 MHz, CDCl_3_) *δ* 12.15 (s, 1H), 7.71 (t, *J* = 7.0 Hz, 3H), 7.62 (d, *J* = 8.2 Hz, 1H), 7.43–7.34 (m, 4H), 7.29 (t, *J* = 7.5 Hz, 1H), 6.98 (s, 2H), 6.73 (d, *J* = 3.3 Hz, 1H), 6.45 (d, *J* = 3.3 Hz, 1H), 4.83 (s, 1H), 1.91 (s, 3H); ^13^C NMR (125 MHz, CDCl_3_) *δ* 162.1, 156.0, 155.4, 153.8, 151.2, 145.1, 136.3, 130.1, 129.8, 129.2, 127.8, 127.6, 126.0, 124.2, 121.2, 111.8, 110.4, 108.6, 108.1, 95.5, 54.5, 30.6, 10.0; HRMS (ESI^+^) *m*/*z*: calcd. for C_26_H_19_N_4_O_3_ [M + H]^+^ 435.14517; found 435.14582.

#### 2.2.2. 6-Amino-3-methyl-4-(5-(2-(*p*-tolyl)benzofuran-3-yl)furan-2-yl)-1,4-dihydropyrano[2,3-*c*]pyrazole-5-carbonitrile (4d)

Orange solid; 94% yield; ^1^H NMR (500 MHz, CDCl_3_) *δ* 12.15 (s, 1H), 7.69 (d, *J* = 7.8 Hz, 1H), 7.59 (dd, *J* = 11.8, 8.1 Hz, 3H), 7.34 (t, *J* = 7.7 Hz, 1H), 7.27 (t, *J* = 7.6 Hz, 1H), 7.20 (d, *J* = 7.8 Hz, 2H), 6.99 (s, 2H), 6.70 (d, *J* = 3.3 Hz, 1H), 6.45 (d, *J* = 3.3 Hz, 1H), 4.83 (s, 1H), 2.31 (s, 3H), 1.92 (s, 3H); ^13^C NMR (125 MHz, CDCl_3_) *δ* 162.0, 155.9, 155.4, 153.7, 151.5, 145.3, 139.6, 136.3, 129.8, 127.9, 127.5, 127.2, 125.8, 124.1, 121.2, 121.0, 111.7, 111.7, 108.6, 107.4, 95.5, 54.5, 30.6, 21.5, 10.0; HRMS (ESI^+^) *m*/*z*: calcd. for C_27_H_21_N_4_O_3_ [M + H]^+^ 449.16082; found 449.16207.

#### 2.2.3. 6-Amino-3-methyl-4-(5-(2-(naphthalen-1-yl)benzofuran-3-yl)furan-2-yl)-1,4-dihydropyrano[2,3-*c*]pyrazole-5-carbonitrile (4f)

Orange solid; 91% yield; ^1^H NMR (500 MHz, CDCl_3_) *δ* 12.07 (s, 1H), 8.09 (d, *J* = 8.3 Hz, 1H), 8.03–8.00 (m, 2H), 7.78–7.75 (m, 1H), 7.66 (d, *J* = 8.3 Hz, 1H), 7.61 (t, *J* = 7.7 Hz, 2H), 7.55–7.52 (m, 1H), 7.45–7.41 (m, 2H), 7.35 (dd, *J* = 9.3, 6.0 Hz, 1H), 6.97 (s, 2H), 6.07 (d, *J* = 3.3 Hz, 1H), 6.00 (d, *J* = 3.4 Hz, 1H), 4.67 (s, 1H), 1.74 (s, 3H); ^13^C NMR (125 MHz, CDCl_3_) *δ* 162.2, 156.1, 155.3, 154.5, 150.2, 146.2, 142.8, 136.3, 133.7, 131.5, 131.0, 130.0, 129.1, 127.8, 127.6, 126.9, 126.4, 125.9, 125.4, 124.2, 122.0, 121.3, 112.0, 111.0, 108.6, 107.6, 95.4, 54.1, 30.7, 9.9; HRMS (ESI^+^) *m*/*z*: calcd. for C_30_H_21_N_4_O_3_ [M + H]^+^ 485.16082; found 485.16019.

#### 2.2.4. 6-Amino-4-(5-(2-(4-methoxyphenyl)benzofuran-3-yl)furan-2-yl)-3-methyl-1,4-dihydropyrano[2,3-*c*]pyrazole-5-carbonitrile (4h)

Orange solid; 93% yield; ^1^H NMR (500 MHz, CDCl_3_) *δ* 12.17 (s, 1H), 7.66 (dd, *J* = 18.5, 8.2 Hz, 3H), 7.59 (d, *J* = 8.2 Hz, 1H), 7.33 (t, *J* = 7.7 Hz, 1H), 7.26 (t, *J* = 7.5 Hz, 1H), 7.00 (s, 2H), 6.93 (dd, *J* = 7.1, 4.9 Hz, 2H), 6.70 (d, *J* = 3.3 Hz, 1H), 6.46 (d, *J* = 3.3 Hz, 1H) 4.84 (s, 1H), 3.79 (s, 3H), 1.92 (s, 3H); ^13^C NMR (125 MHz, CDCl_3_) *δ* 162.0, 160.5, 155.6, 153.6, 151.4, 145.4, 136.4, 132.0, 129.1, 128.0, 126.0, 124.1, 122.4, 120.9, 118.0, 114.6, 111.6, 110.0, 108.7, 106.6, 95.5, 55.8, 54.5, 30.6, 10.0; HRMS (ESI^+^) *m*/*z*: calcd. for C_27_H_21_N_4_O_4_ [M + H]^+^ 465.15574; found 465.15567.

#### 2.2.5. 6-Amino-3-methyl-4-(5-(2-pentylbenzofuran-3-yl)furan-2-yl)-1,4-dihydropyrano[2,3-*c*]pyrazole-5-carbonitrile (4j)

Orange solid; 95% yield; ^1^H NMR (500 MHz, CDCl_3_) *δ* 12.16 (s, 1H), 7.79–7.72 (m, 1H), 7.54–7.47 (m, 1H), 7.28–7.20 (m, 2H), 6.98 (s, 2H), 6.68 (d, *J* = 3.3 Hz, 1H), 6.32 (d, *J* = 3.3 Hz, 1H), 4.83 (s, 1H), 2.95–2.84 (m, 2H), 1.97 (s, 3H), 1.59 (*p*, *J* = 7.2 Hz, 2H), 1.25–1.16 (m, 4H), 0.78 (t, *J* = 6.5 Hz, 3H); ^13^C NMR (125 MHz, CDCl_3_) *δ* 162.2, 155.7, 155.5, 155.2, 153.8, 146.5, 136.2, 126.2, 124.7, 123.7, 121.3, 120.7, 111.5, 107.9 (2C), 107.8, 95.4, 63.3, 54.4, 31.2, 27.9, 27.7, 22.4, 14.4, 10.1; HRMS (ESI^+^) *m*/*z*: calcd. for C_25_H_25_N_4_O_3_ [M + H]^+^ 429.19212; found 429.19265.

### 2.3. Drug-likeness and ADMET evaluation

The drug-likeness and ADMET (absorption, distribution, metabolism, excretion, and toxicity) profiles of the synthesized benzofuran-furan-pyrano[2,3-*c*]pyrazole hybrids (4b, 4d, 4f, 4h and 4j), together with the reference drugs seliciclib (SEL), enzalutamide (ENZ), tivozanib (TIV), and venetoclax (VEN), were evaluated using the ADMETlab 2.0 online platform (https://admetmesh.scbdd.com/).^22^ All compounds were submitted in Simplified Molecular Input Line Entry System (SMILES) format. Drug-likeness was assessed according to Lipinski's Rule of Five using the criteria of molecular weight (*M*_W_) ≤ 500 g mol^−1^, log *P* ≤ 5, hydrogen bond donors (HBD) ≤ 5, and hydrogen bond acceptors (HBA) ≤ 10, while compounds with fewer than two violations were considered acceptable.^23^ In addition, the topological polar surface area (TPSA) was calculated, and values within 0–140 A^2^ were regarded as favorable for intestinal absorption and membrane permeability. Absorption parameters included human colorectal adenocarcinoma cell line (Caco-2) permeability, for which values > −5.15 cm s^−1^ were considered favorable, and Madin–Darby canine kidney (MDCK) permeability, classified as low (<2 × 10^−6^ cm s^−1^), medium (2–20 × 10^−6^ cm s^−1^), or high (>20 × 10^−6^ cm s^−1^). Human intestinal absorption (HIA) and oral bioavailability (*F*_20%_) were interpreted according to the ADMETlab probability scale as excellent (0–0.3), medium (0.3–0.7), and poor (0.7–1.0). Distribution was evaluated through plasma protein binding (PPB), with values ≤90% considered acceptable, volume of distribution (VD) with an optimal range of 0.04–20 L kg^−1^, blood–brain barrier (BBB) penetration interpreted as excellent (0–0.3), medium (0.3–0.7), and poor (0.7–1.0), and fraction unbound (Fu) categorized as high (>20%), medium (5–20%), and low (<5%). Metabolic properties were predicted from the probability of the compounds acting as inhibitors or substrates of major cytochrome P450 isoforms, including CYP1A2, CYP3A4, CYP2C9, CYP2C19, and CYP2D6, where 0 indicates non-inhibitor/non-substrate and 1 indicates inhibitor/substrate. Excretion-related parameters included clearance (CL), classified as high (>15 mL min^−1^ kg^−1^), moderate (5–15 mL min^−1^ kg^−1^), and low (<5 mL min^−1^ kg^−1^), and half-life (*T*_1/2_), interpreted as excellent (0–0.3), medium (0.3–0.7), and poor (0.7–1.0). Toxicity assessment comprised human ether-à-go-go-related gene (hERG) inhibition, skin sensitization, carcinogenicity, eye corrosion, and eye irritation, all interpreted on the same probability scale of excellent (0–0.3), medium (0.3–0.7), and poor (0.7–1.0). This computational evaluation enabled a comparative assessment of the synthesized hybrids and the reference drugs in terms of drug-likeness, pharmacokinetic behavior, and safety profile.

### 2.4. Molecular docking studies

#### 2.4.1. Protein preparation

The three-dimensional crystal structures of four target proteins, namely cyclin-dependent kinase 2 (CDK2; PDB ID: 1HCK), androgen receptor ligand-binding domain (AR; PDB ID: 2AMA), vascular endothelial growth factor receptor 2 (VEGFR-2; PDB ID: 4ASE), and B-cell lymphoma 2 (Bcl-2; PDB ID: 4IEH), were retrieved from the RCSB Protein Data Bank (https://www.rcsb.org/).^24^ Prior to docking, all crystallographic water molecules, co-crystallized ligands, and other heteroatoms were removed from the protein structures. The prepared proteins were then processed using AutoDock Tools (version 1.5.6) by adding polar hydrogen atoms and Kollman charges, and the resulting receptor files were saved in PDBQT format for subsequent docking analysis. These targets were chosen on the basis of their mechanistic relevance to anticancer action and their simple structural accessibility. CDK2 plays a pivotal role as a controller of cell-cycle progression and tumor proliferation,^25^ AR is a central controller of hormonally controlled cancer signaling,^26^ VEGFR-2 plays a critical role in tumor angiogenesis,^27^ and Bcl-2 has a potent role in the cell survival of the tumor.^28^ Accordingly, docking studies were conducted to investigate the possible molecular interactions of the synthesized compounds with proteins involved in proliferation, angiogenesis, hormonal signaling, and apoptosis.

#### 2.4.2. Ligand preparation

The chemical structures of the synthesized benzofuran-furan-pyrano[2,3-*c*]pyrazole hybrids (4b, 4d, 4f, 4h and 4j) and the reference drugs SEL, ENZ, TIV, and VEN were sketched using ChemDraw Professional 16.0 and subsequently energy-minimized in Chem3D using the MMFF94 force field with a root mean square (RMS) gradient of 0.1. The optimized ligand structures were imported into AutoDock Tools (version 1.5.6), where non-polar hydrogen atoms were merged, Gasteiger charges were assigned, and rotatable bonds were defined. The prepared ligands were then saved in PDBQT format for docking calculations.

#### 2.4.3. Docking analysis

Molecular docking simulations were carried out using AutoDock Tools (version 1.5.6). Grid boxes were defined around the active sites of the target proteins based on the positions of the co-crystallized ligands. The docking calculations were performed by applying the Genetic Algorithm to explore the preferred binding conformations of the ligands within the receptor binding pockets.^29^ To validate the docking protocol, the native co-crystallized ligands were re-docked into their respective active sites, and the accuracy of the docking procedure was evaluated by calculating the root mean square deviation (RMSD) between the docked and crystallographic conformations; RMSD values below 2.0 A were considered acceptable.^30^ The binding modes and intermolecular interactions were visualized using BIOVIA Discovery Studio Visualizer (version 2021). The docking poses were ranked according to their predicted binding energies, and the resulting data were used for the relative comparison of ligand–protein interactions under identical docking conditions. All calculations were performed on a Windows 10 (64-bit) operating system equipped with an Intel Core i5-6200U CPU and 16 GB RAM.

### 2.5. Anticancer activity

#### 2.5.1. Cell viability assay (MTT assay)

The cytotoxic effects of synthesized benzofuran-furan-pyrano[2,3-*c*]pyrazole hybrids were evaluated against human breast (MDA-MB-231), colon (Caco-2), lung (A549), and prostate (LNCaP) cancer cell lines, as well as normal human umbilical vein endothelial cells (HUVEC). All cell lines used in this study were obtained from the European Collection of Cell Cultures (ECACC). Cells were cultured in DMEM or RPMI-1640 medium supplemented with 10% fetal bovine serum (FBS) and 1% penicillin/streptomycin at 37 °C in a 95% humidified incubator with 5% CO_2_ as described previously.^31^ For the MTT assay, cells were seeded into 96-well plates at a density of approximately 2 × 10^3^ cells per well and incubated overnight. The cells were then treated with varying concentrations of the test compounds for 24 h. Compounds were dissolved in dimethyl sulfoxide (DMSO), with the final solvent concentration not exceeding 0.5% (v/v). Following treatment, 10 µL of MTT solution (5 mg mL^−1^) was added to each well and incubated for 2–4 h at 37 °C. Subsequently, formazan crystals were dissolved with 50 µL of DMSO, and absorbance was measured at 590 nm using a microplate reader Epoch spectrophotometer (BioTek, USA). Cell viability was expressed as a percentage relative to untreated controls, and half-maximal effective concentration (EC_50_) values were calculated and used for subsequent experiments.^32^

#### 2.5.2. Apoptosis analysis

The apoptotic effects of the synthesized benzofuran-furan-pyrano[2,3-*c*]pyrazole hybrids on Caco-2, LNCaP, A549, and MDA-MB-231 cancer cell lines were evaluated using an Annexin V-FITC/PI Apoptosis Detection Kit (Elabscience, E-CK-A211). Cells were cultured in DMEM or RPMI-1640 medium supplemented with 10% fetal bovine serum (FBS) and 1% penicillin/streptomycin and maintained at 37 °C in a humidified atmosphere containing 5% CO_2_. Cells were subcultured at 80–90% confluency, counted, and seeded prior to treatment. For apoptosis analysis, 3 × 10^4^ cells per well were seeded into 6-well plates containing 3 mL of complete culture medium and incubated for 24 h to allow cell attachment. Cells were then treated with the test compounds at their respective EC_50_ concentrations and incubated for an additional 24 h. Dimethyl sulfoxide (DMSO) was used as the negative control, while paclitaxel (PAX) served as the positive control.

Following treatment, the culture supernatant was removed, and cells were detached using 300 µL of trypsin-EDTA and incubated for 3 min. The resulting cell suspensions were collected into microcentrifuge tubes and centrifuged at 2000 rpm for 5 min. The supernatant was discarded, and the cell pellets were washed twice with phosphate-buffered saline (PBS). Each pellet was resuspended in 100 µL of 1× Annexin V binding buffer, followed by the addition of 2.5 µL Annexin V-FITC and 2.5 µL propidium iodide (PI). Samples were gently mixed and incubated for 20–25 min at room temperature in the dark. Subsequently, 400 µL of 1× binding buffer was added to each sample, and apoptotic cell populations were analyzed using a CytoFLEX flow cytometer. The percentages of viable, early apoptotic, late apoptotic, and necrotic cells were quantified.^33^

#### 2.5.3. Real-time PCR analysis of apoptosis-related mRNA expression

The mRNA expression levels of apoptosis-related genes were quantified by real-time quantitative polymerase chain reaction (RT-qPCR) following treatment of cancer cell lines with benzofuran-6-amino-3-methyl-1,4-dihydropyrano(2,3-*c*)pyrazole-5-carbonitrile derivatives at their respective EC_50_ concentrations. Total RNA was isolated using a commercial extraction kit according to the manufacturer's instructions (GeneAll Biotechnology, Korea). RNA concentration, purity, and integrity were assessed by spectrophotometric analysis (A260/A280) and agarose gel electrophoresis.

Purified RNA (2.5 µg per reaction) was reverse-transcribed into complementary DNA (cDNA) using the OneScript Plus cDNA Synthesis Kit (abm, Canada). Gene-specific primers for BAX, BCL2, CASP3, CASP8, CASP9, and P53 were designed based on GenBank/EMBL database sequences (Table 1). Quantitative PCR amplification was performed using BlasTaq™ 2× qPCR MasterMix (abm, Canada) in a real-time PCR system.

**Table 1:** Sequences of primers used for RT-PCR in current study

Gene	Forward primer	Reverse primer
*GAPDH*	TGTGGGCATCAATGGATTTGG	ACACCATGTATTCCGGGTCAAT
*BAX*	TCAGGATGCGTCCACCAAGAAG	TGTGTCCACGGCGGCAATCATC
*BCL2*	ATCGCCCTGTGGATGACTGAGT	GCCAGGAGAAATCAAACAGAGGC
*CASP3*	GGAAGCGAATCAATGGACTCTGG	GCATCGACATCTGTACCAGACC
*CASP8*	AGAAGAGGGTCATCCTGGGAGA	TCAGGACTTCCTTCAAGGCTGC
*CASP9*	GTTTGAGGACCTTCGACCAGCT	CAACGTACCAGGAGCCACTCTT
*P53*	CCTCAGCATCTTATCCGAGTGG	TGGATGGTGGTACAGTCAGAGC

Relative gene expression levels were calculated after normalization to the internal reference gene GAPDH using the comparative 2 −ΔΔCt method, as previously described.^34^

#### 2.5.4. Measurement of intracellular reactive oxygen species (ROS)

Intracellular reactive oxygen species (ROS) levels were quantified using a DCFH-DA-based assay (Elabscience, E-BC-K138-F). Cells were seeded in 6-well plates at a density of approximately 3 × 10^4^ cells per well and incubated overnight at 37 °C in a humidified atmosphere containing 5% CO_2_. Cells were then treated with the EC_50_ concentrations of the test compounds for 24 h. *Tert*-butyl hydroperoxide (TBHP) was used as the positive control.

Following treatment, cells were incubated with DCFH-DA for 45 min (except for the negative control), harvested by trypsinization, and washed twice with serum-free medium by centrifugation at 1000×*g* for 7 min. The final cell pellet was resuspended in 500 µL of serum-free medium, and approximately 3 × 10^4^ cells per sample were analyzed by CytoFLEX flow cytometry (Beckman Coulter). ROS levels were expressed as fluorescence intensity relative to the control samples.^35^

#### 2.5.5. Flow cytometric analysis of mitochondrial membrane potential (MMP)

Mitochondrial membrane potential (MMP) was evaluated using a JC-1 fluorescent probe-based assay (Elabscience, ECK-A301). Cells were seeded in 6-well plates at a density of approximately 3 × 10^4^ cells per well and incubated overnight at 37 °C in a humidified atmosphere containing 5% CO_2_. Cells were then treated with the benzofuran pyrano-pyrazole derivatives at their respective EC_50_ concentrations for 24 h.

Following treatment, the culture medium was replaced, and carbonyl cyanide 3-chlorophenylhydrazone (CCCP, 150 µM) was added to the positive control wells and incubated for 5 min at 37 °C. JC-1 dye (final concentration prepared from a 200 µM stock solution) was then added to all wells and incubated for 15–20 min at 37 °C in 5% CO_2_. Cells were washed with PBS, harvested by trypsinization, and centrifuged at 400×*g* for 4 min at 4 °C. The resulting cell pellets were resuspended in 500 µL of PBS, and approximately 3 × 10^4^ cells per sample were analyzed by Beckman Coulter flow cytometry.

Changes in MMP were expressed as the ratio of red (JC-1 aggregates) to green (JC-1 monomers) fluorescence relative to the control samples as previously described.^36^

#### 2.5.6. Cell cycle analysis

Cell cycle distribution was analyzed using a fluorometric assay kit (Elabscience, E-CK-A351). Cells were seeded in 6-well plates at a density of approximately 3 × 10^4^ cells per well and incubated for 24 h at 37 °C in a humidified atmosphere containing 5% CO_2_. Cells were then treated with the test compounds at their respective EC_50_ concentrations for an additional 24 h.

Following treatment, cells were harvested by trypsinization, washed with PBS, and fixed in ice-cold ethanol (final concentration ∼70%) at −20 °C for overnight. After fixation, cells were washed with PBS and incubated with RNase A at 37 °C for 30 min to remove residual RNA. Subsequently, cells were stained with propidium iodide (PI) for 30 min in the dark at 4 °C.

Cell cycle profiles were acquired using a CytoFLEX flow cytometer (Beckman Coulter), and the proportions of cells in the G_0_/G_1_, S, and G_2_/M phases were quantified using standard analysis software as previously described.^17^

#### 2.5.7. Transwell matrigel invasion assay

The effects of benzofuran-furan-pyrano[2,3-*c*]pyrazole hybrids on the invasive potential of cancer cells were evaluated using a Transwell Matrigel invasion assay (Corning, USA).

The Matrigel inserts were thawed at −20 °C, rehydrated with serum-free culture medium in both the upper and lower chambers, and activated by incubation at 37 °C in a humidified atmosphere containing 5% CO_2_ for 2 h. Subsequently, complete culture medium supplemented with 20% fetal bovine serum (FBS) was added to the lower wells as a chemoattractant. Cells (3 × 10^4^ per insert) were suspended in serum-free medium and treated with the derivatives at their respective EC_50_ concentrations before being seeded into the upper chamber. After 24 h of incubation, non-invading cells on the upper surface of the membrane were gently removed using a cotton swab. Invaded cells on the lower surface were fixed with cold methanol and stained with crystal violet.

Images were captured using an inverted light microscope, and the number of invaded cells was quantified using ImageJ software. The results were expressed relative to the negative control.^37^

#### 2.5.8. Wound healing (scratch) assay for the evaluation of cancer cell migration

The effects of the derivatives on cancer cell migration were evaluated using a wound healing (scratch) assay. Cells were harvested using trypsin-EDTA, counted, and seeded into 6-well culture plates at a density of approximately 4 × 10^4^ cells per well. After overnight incubation and upon reaching 80–90% confluence, a linear scratch was created across the cell monolayer using a sterile 200 µL pipette tip.

Detached cells were removed by washing with PBS, and baseline images of the wound area (0 h) were captured using an inverted microscope. Cells were then incubated in complete medium containing the test compounds at their respective EC_50_ concentrations, while control wells received medium alone. Wound closure was monitored and imaged at 24 and 48 h. Images were analyzed with the help of the ImageJ program and the results were evaluated by comparing with the negative control group as described previously.^38^

#### 2.5.9. Colony formation assay

The clonogenic potential of MDA-MB-231, A549, LNCaP, and Caco-2 cells was assessed using a colony formation assay. Cells were seeded into 6-well plates at a density of 1 × 10^3^ cells per well and incubated for 24 h at 37 °C in a humidified atmosphere containing 5% CO_2_. Subsequently, cells were treated with the derivatives at their respective EC_50_ concentrations for 24 h.

Following treatment, the medium was replaced with fresh complete medium, and the cells were maintained for 10–14 days, with the medium renewed every 3–4 days. At the end of the incubation period, colonies were fixed with cold methanol (−20 °C for 25 min) and stained with 0.4% crystal violet for 5 min. Excess stain was removed by washing with phosphate-buffered saline (PBS), and the plates were air-dried at room temperature.

Colonies were visualized and counted using an inverted microscope. Quantitative analysis was performed using ImageJ software, and the results were expressed relative to the untreated control group, as previously described.^39^

#### 2.5.10. Statistical analysis

All experiments were performed in triplicate, and data are presented as mean ± standard deviation (SD). Statistical analyses were conducted using SPSS (v23.0) and GraphPad Prism (v9.0). Differences between two groups were evaluated using Student's *t*-test, while comparisons among multiple groups were performed by one-way analysis of variance (ANOVA). Graphs and result visualizations were generated using GraphPad Prism. A *p* value of less than 0.05 was considered statistically significant.

## 3. Results and discussion

### 3.1. Synthesis of benzofuran-furan-pyrano[2,3-*c*]pyrazole hybrids

The synthetic pathway for the preparation of the target benzofuran-furan-pyrano[2,3-*c*]pyrazole hybrids (4b, 4d, 4f, 4h and 4j) is illustrated in Scheme 1, while the chemical structures of the final compounds are shown in Fig. 1. The designed molecules were constructed through a molecular hybridization strategy combining three biologically relevant heterocyclic fragments, namely benzofuran, furan, and pyrano[2,3-*c*]pyrazole, into a single scaffold. This structural combination was intended to increase molecular diversity and provide compounds with promising physicochemical and biological properties. As shown in Scheme 1, the final compounds (4b, 4d, 4f, 4h and 4j) were synthesized from the corresponding benzofuran-furan aldehyde intermediates (3b, 3d, 3f, 3h and 3j) through a one-pot multicomponent reaction with malononitrile and 3-methyl-1*H*-pyrazol-5-ol in the presence of K_2_CO_3_ as a mild base. The reaction was performed in ethanol at room temperature. TLC monitoring showed complete consumption of the corresponding benzofuran-furan aldehyde after approximately 2 min, concomitant with formation of the solid product. The reaction was therefore stopped by discontinuing stirring and immediately isolating the precipitated product by filtration, followed by washing with cold ethanol and recrystallization. No separate chemical quenching step was required under these conditions. The five derivatives were obtained in isolated yields ranging from 91 to 96%. The rapid formation of the products was consistently observed for all five derivatives investigated in the present study. Furthermore, the same synthetic protocol was successfully reproduced for additional structurally related derivatives and at larger reaction scales, supporting the reproducibility and scalability of the reaction conditions. Detailed results for these additional derivatives and scale-up experiments are beyond the scope of the present study and will be reported separately in future work.

**Scheme 1:**
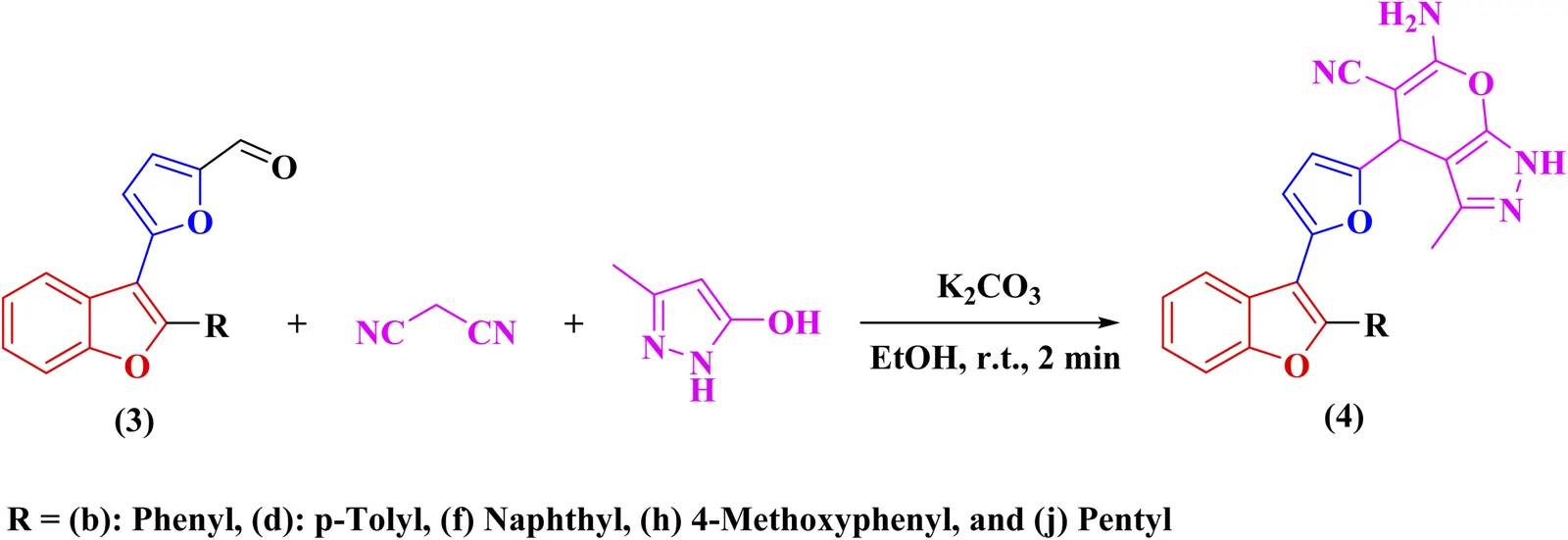
Synthetic route for the preparation of benzofuran-furan-pyrano[2,3-*c*]pyrazole hybrids (4b, 4d, 4f, 4h and 4j).

**Fig. 1:**
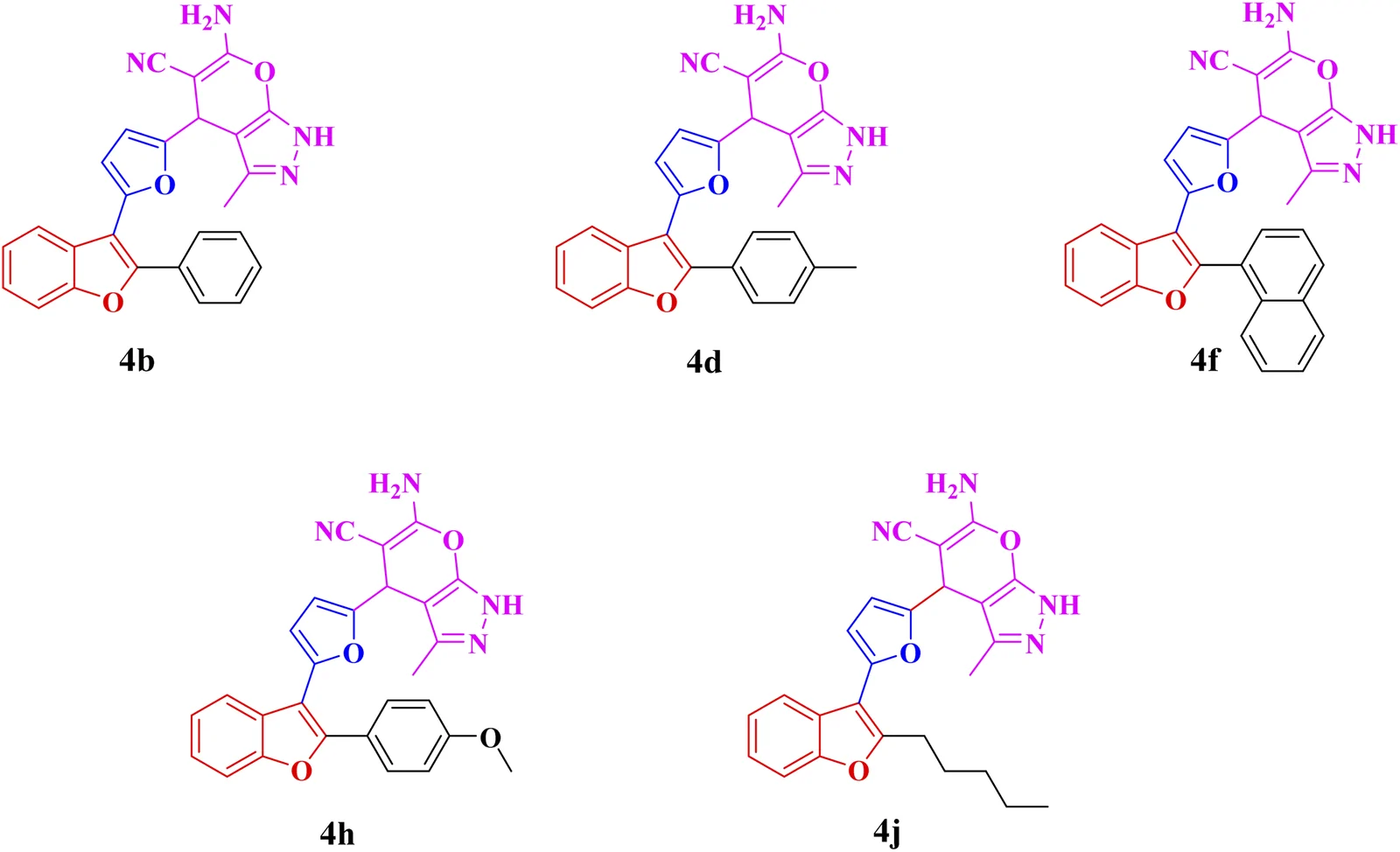
Chemical structures of the synthesized benzofuran-furan-pyrano[2,3-*c*]pyrazole hybrids (4b, 4d, 4f, 4h and 4j).

Several rapid synthetic approaches to pyrano[2,3-*c*]pyrazole derivatives have previously been reported. Kashtoh *et al.* synthesized dihydropyrano[2,3-*c*]pyrazoles using pyrazolones and preformed benzylidene derivatives in the presence of triethylamine at room temperature, with reaction times of 25–30 min.^40^ More recently, Subrahmanyam *et al.* reported a one-pot four-component synthesis using a Fe_5_(PW_10_V_2_O_40_)_3_ nanocatalyst in ethanol at room temperature, affording pyrano[2,3-*c*]pyrazole derivatives in 88–98% yields in less than 15 min.^41^ In comparison, the present protocol employs K_2_CO_3_ (10 mol%) in ethanol at room temperature and provides the target benzofuran-furan-pyrano[2,3-*c*]pyrazole hybrids within approximately 2 min in 91–96% isolated yields, representing a substantially shorter reaction time under mild conditions.

The final series included five derivatives bearing different substituents on the benzofuran moiety, as presented in Fig. 1. Compounds 4b, 4d, 4f, 4h and 4j contain phenyl, *p*-tolyl, naphthyl, 4-methoxyphenyl, and pentyl substituents, respectively. These structural variations allowed the preparation of both aromatic and aliphatic derivatives, enabling further evaluation of the influence of substituent type on the predicted drug-likeness, ADMET behavior, and molecular docking interactions. Accordingly, compounds 4b, 4d, 4f, 4h and 4j were selected for biological evaluation to represent this structural diversity and to enable comparative assessment of the final benzofuran-furan-pyrano[2,3-*c*]pyrazole scaffold. In contrast, compounds of the 1, 2, and 3 series served as synthetic intermediates in the preparation of the final hybrids and were not included in the biological screening. Therefore, their absence from the biological evaluation reflects the predefined scope of the present study rather than a lack of biological activity. Structure-based searches of the SciFinder and Reaxys databases were performed for compounds 4b, 4d, 4f, 4h and 4j. No identical structures were identified in either database at the time of the search, indicating that these benzofuran-furan-pyrano[2,3-*c*]pyrazole hybrids have not previously been reported.

The proposed reaction pathway for the formation of compounds (4b, 4d, 4f, 4h and 4j) is depicted in Scheme 2. Initially, the aldehyde group of intermediate 3 undergoes Knoevenagel condensation with malononitrile under basic conditions, accompanied by the elimination of water to generate an activated benzofuran-furan arylidene malononitrile intermediate. This electron-deficient intermediate then reacts with 3-methyl-1*H*-pyrazol-5-ol through a Michael-type addition. Subsequently, intramolecular cyclization occurs through nucleophilic attack on the cyano group, followed by proton transfer and tautomerization, leading to the formation of the fused pyrano[2,3-*c*]pyrazole ring system.

**Scheme 2:**
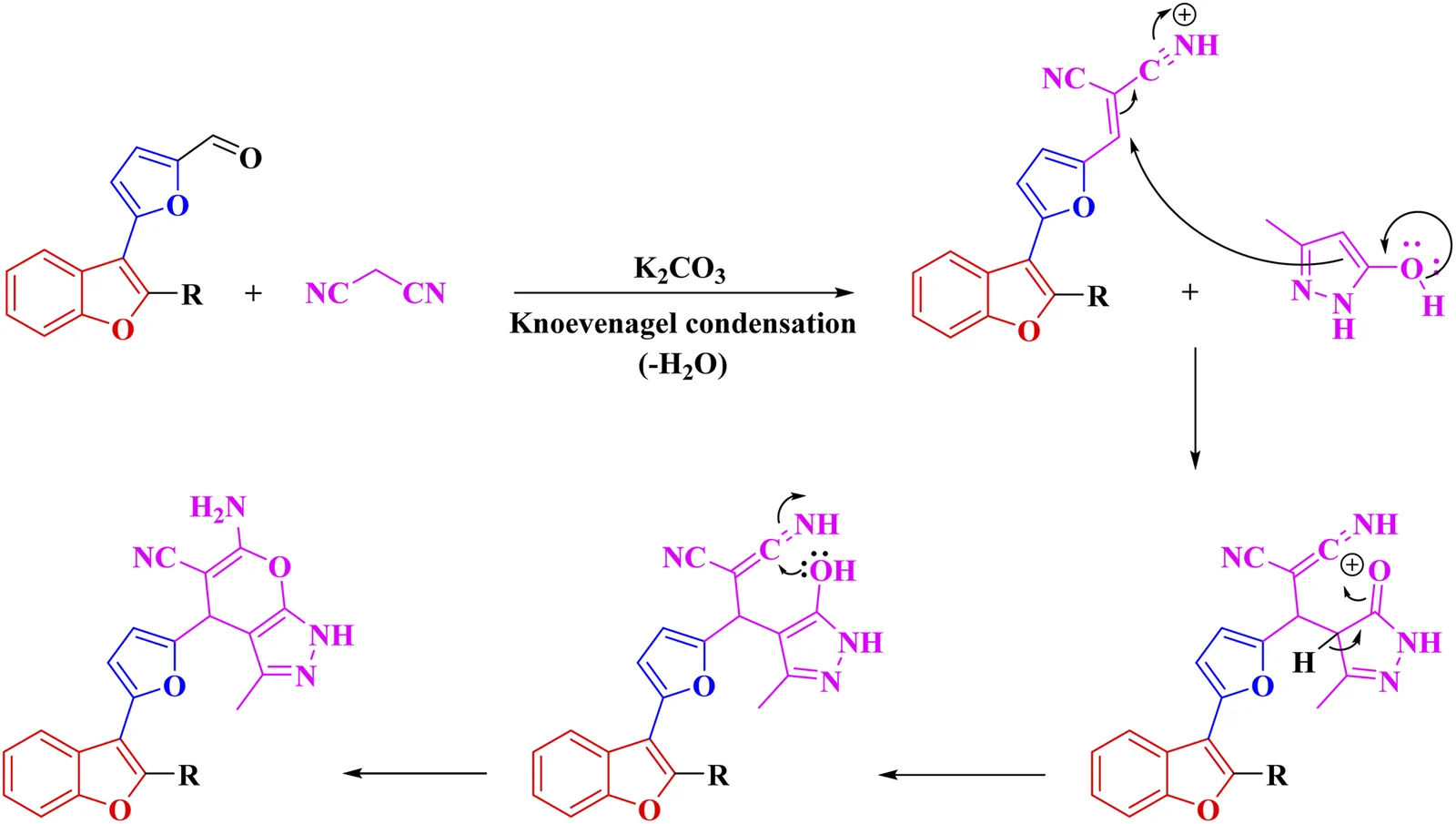
Proposed reaction mechanism for the formation of benzofuran-furan-pyrano[2,3-*c*]pyrazole hybrids (4b, 4d, 4f, 4h and 4j).

All final compounds were confirmed by nuclear magnetic resonance (NMR) spectroscopy, and the corresponding spectral data are provided in the SI.

### 3.2. *In silico* studies

#### 3.2.1. Drug-likeness and ADMET assessment

Early-stage pharmacokinetic screening plays a pivotal role in identifying promising drug candidates by providing preliminary insight into their oral bioavailability, membrane permeability, and overall drug-likeness.^42^ Among the most widely applied criteria for this purpose is Lipinski's Rule of Five, which states that orally active compounds generally possess a molecular weight (*M*_W_) ≤ 500 g mol^−1^, log *P* ≤ 5, no more than 5 hydrogen bond donors (HBD), and no more than 10 hydrogen bond acceptors (HBA). In general, compounds containing at most two violations of these parameters are said to have acceptable drug-like properties. In Table 2, the synthesized benzofuran-furan-pyrano[2,3-*c*]pyrazole hybrids (4b, 4d, 4f, 4h and 4j) were generally well-behaved according to Lipinski, with all of the hybrids described as accepted. Only one violation was observed in each derivative and was associated with slightly high values of log *P* ranging from 5.341 to 6.263. However, all of the synthesized compounds met the rest of the parameters, with *M*_W_ values of 428.18–484.15 g mol^−1^, HBD of 3, and HBA of 7–8 being within acceptable parameters. Moreover, TPSA values of compounds (4b, 4d, 4f, 4h and 4j) were observed to be in the favorable range of 0–140 A^2^ (114.00–123.23 A^2^), which is typically attributed to acceptable intestinal absorption and cell membrane permeability. Comparing the reference drugs SEL, ENZ, and TIV, all drugs were in complete agreement with Lipinski criteria and had no violations, which justifies their behavior as accepted drug analogs. In comparison, VEN was classified as rejected due to the three violations, including excessive *M*_W_ (867.32 g mol^−1^), log *P* (7.203), and HBA (14), as well as a TPSA value (172.03 A^2^) that is above the recommended value. These results suggest that despite the synthesized benzofuran-furan-pyrano[2,3-*c*]pyrazole hybrids (4b, 4d, 4f, 4h and 4j) having higher lipophilicity, their good agreement with the other major physicochemical descriptors, along with their good TPSA values, supports their potential as useful drug-like candidates for further pharmacokinetic and biological evaluation.

**Table 2:** Physicochemical properties of benzofuran-furan-pyrano[2,3-*c*]pyrazole hybrids (4b, 4d, 4f, 4h and 4j) and the reference drugs SEL, ENZ, TIV, and VEN

Compound	*M*_W_	log *P*	HBD	HBA	No. of violations	Decision	TPSA
	≤500	≤5	≤ 5	≤ 10	≤2		0–140
4b	434.14	5.341	3	7	1	Accepted	114.00
4d	448.15	5.781	3	7	1	Accepted	114.00
4f	484.15	6.263	3	7	1	Accepted	114.00
4h	464.15	5.399	3	8	1	Accepted	123.23
4j	428.18	5.892	3	7	1	Accepted	114.00
SEL	354.22	3.516	3	7	0	Accepted	87.89
ENZ	464.09	3.011	1	6	0	Accepted	76.44
TIV	454.10	4.500	2	9	0	Accepted	107.74
VEN	867.32	7.203	3	14	3	Rejected	172.03

Table 3 presents the comparison of the predicted ADMET properties of the synthesized benzofuran-furan-pyrano[2,3-*c*]pyrazole hybrids (4b, 4d, 4f, 4h and 4j) and the reference drugs SEL, ENZ, TIV, and VEN. This discussion offers the overall pharmacokinetic and safety profiles of the studied compounds and gives insight into their applicability as drug candidates in the next stages of scientific development. Compounds (4b, 4d, 4f, 4h and 4j) behaved very promisingly from the absorption point of view. Caco-2 permeability was found to be between −4.947 and −4.923, with all values being above the acceptable threshold, meaning that they showed good predicted intestinal permeability. Similarly, the efficiency of passive membrane permeability is also supported by the MDCK permeability values (1.2 × 10^−5^–1.5 × 10^−5^ cm s^−1^). The extremely low HIA values (0.008–0.023) and *F*_20%_ (0.004–0.020) also indicate good intestinal absorption and decent oral bioavailability. The synthesized hybrids exhibited a more uniform absorption pattern compared with the reference drugs, namely ENZ and VEN, particularly the latter, which was the least favorable in terms of Caco-2 permeability. These data imply that the synthesized compounds have appropriate absorption properties to be considered orally active. The pharmacokinetic potential of the synthesized series is also supported by the distribution data. The VD of (4b, 4d, 4f, 4h and 4j) was predicted to be 0.544–1.717 L kg^−1^, which fell within the acceptable range, meaning that there was good tissue distribution. The BBB values of compounds (4b, 4d, 4f and 4h) were also among the lowest (0.010–0.038), with that of 4j being slightly higher (0.357), which implies that the extensive penetration of many of these derivatives through the blood–brain barrier is unlikely. In the case of anticancer agents that are designed to work outside the central nervous system, such behavior can be viewed as an additional benefit, as it might decrease the occurrence of CNS-related side effects. Nonetheless, the PPB values of the synthesized compounds were extremely high (100.2–100.9%), and Fu values were equally low (0.792–0.888%), meaning that a very low percentage of each compound would be circulating freely as a drug. This high binding tendency can reduce the accessible active fraction, despite the otherwise acceptable distribution profile. The predicted metabolic activity of compounds (4b, 4d, 4f, 4h and 4j) is characterized by a strong probability of interaction with key cytochrome P450 isoenzymes. The compounds were strong inhibitors of CYP1A2, CYP3A4, CYP2C9, CYP2C19, and CYP2D6, with most of them being near or higher than 0.8, indicating a significant possibility of disrupting enzyme metabolism and affecting drug metabolism. Simultaneously, moderate and high substrate probabilities were also determined for a variety of different isoforms, especially CYP3A4, CYP2C9, and CYP2D6, meaning that these compounds themselves could be metabolized through these pathways. The given profile indicates that metabolic behavior is a potential vulnerability and that there is a possible risk of drug–drug interactions. SEL showed a relatively lower level of metabolic interaction among the reference drugs compared to the compounds, but ENZ, TIV, and VEN also had significant levels of CYP-related liabilities with selected enzymes. The synthesized hybrids exhibited low values of CL in terms of excretion (3.214–3.874 mL min^−1^ kg^−1^) and therefore showed slower elimination than SEL and ENZ, which recorded higher values of clearance. Their *T*_1/2_ scores (0.008–0.021) were predicted to be within the correct range (0.0–0.02), which reflects an acceptable half-life profile according to the predictive formula. It could suggest long systemic persistence of the synthesized compounds, which might be advantageous in sustaining exposure, although the likelihood of accumulation cannot be ruled out without direct experimentation. One of the most promising factors of the ADMET analysis was the toxicity predictions. All synthesized derivatives had low hERG scores (0.069–0.096), indicating a likely low cardiotoxicity risk. Equally, the skin sensitization scores (0.083–0.183) and carcinogenicity scores (0.045–0.081) were within desirable limits, indicating limited toxicological concern for these endpoints. The values of eye corrosion (0.003 for all compounds) and eye irritation (0.009–0.022) were also not significant. Conversely, not all the reference drugs had favorable safety trends, specifically ENZ, which had high carcinogenicity, and VEN, which had a significantly high hERG value. These comparisons indicate that the synthesized benzofuran-furan-pyrano[2,3-*c*]pyrazole hybrids (4b, 4d, 4f, 4h and 4j) have a more balanced and safer computational toxicity profile than some of the reference agents used in clinics. These data show that the produced hybrids combine good predicted intestinal permeability, favorable oral absorption, acceptable distribution volume, low BBB penetration, and low predicted toxicity, indicating that they have potential as effective drug-like candidates. Nevertheless, their very high plasma protein binding, low unbound fraction, low clearance, and strong CYP interaction potential suggest that additional optimization and experimental pharmacokinetic studies will be necessary to confirm their *in vivo* performance.

**Table 3:** Predicted ADMET propertrano[2,3-*c*]pyrazole hybrids (4b, 4d, 4f, 4h and 4j) and the reference drugs SEL, ENZ, TIV, and VEN

Parameter	4b	4d	4f	4h	4j	SEL	ENZ	TIV	VEN
**Absorption**									
Caco2	−4.924	−4.926	−4.947	−4.923	−4.948	−4.987	−5.184	−5.068	−5.462
MDCK	1.3 × 10−5	1.3 × 10−5	1.2 × 10−5	1.2 × 10−5	1.5 × 10−5	5 × 10−6	2.8 × 10−5	1 × 10−5	2.4 × 10−5
HIA (% not absorbed)	0.008	0.009	0.008	0.009	0.023	0.054	0.014	0.008	0.003
*F*_20%_	0.006	0.005	0.020	0.004	0.004	0.096	0.007	0.001	0.003

**Distribution**									
PPB(%)	100.5	100.8	100.9	100.5	100.2	88.13	94.90	99.50	100.9
VD	1.209	1.177	1.015	0.544	1.717	1.409	1.047	0.306	1.041
BBB	0.038	0.026	0.010	0.017	0.357	0.308	0.964	0.031	0.107
Fu (%)	0.799	0.792	0.811	0.888	0.887	12.78	1.921	1.474	0.379

**Metabolism**									
CYP1A2 inhibitor-substrate	0.964–0.425	0.914–0.650	0.942–0.485	0.929–0.791	0.965–0.862	0.967–0.193	0.475–0.631	0.426–0.951	0.065–0.143
CYP3A4 inhibitor-substrate	0.950–0.868	0.934–0.908	0.940–0.857	0.944–0.904	0.937–0.893	0.801–0.309	0.643–0.921	0.716–0.517	0.919–0.915
CYP2C9 inhibitor-substrate	0.963–0.549	0.956–0.723	0.955–0.784	0.963–0.837	0.965–0.731	0.744–0.248	0.885–0.855	0.857–0.904	0.899–0.926
CYP2C19 inhibitor-substrate	0.976–0.106	0.971–0.114	0.976–0.085	0.973–0.116	0.974–0.294	0.207–0.096	0.886–0.796	0.920–0.685	0.816–0.072
CYP2D6 inhibitor-substrate	0.816–0.663	0.845–0.864	0.794–0.859	0.813–0.892	0.821–0.748	0.085–0.204	0.457–0.255	0.307–0.913	0.170–0.815

**Excretion**									
CL (mL min^−1^ kg^−1^)	3.244	3.257	3.259	3.874	3.214	11.727	6.997	3.478	1.585
*T*_1/2_ (h)	0.020	0.016	0.008	0.021	0.019	0.456	0.122	0.225	0.012

**Toxicity**									
hERG	0.069	0.084	0.076	0.096	0.086	0.115	0.399	0.247	0.942
Skin sensitization	0.128	0.099	0.083	0.105	0.183	0.196	0.310	0.170	0.215
Carcinogencity	0.064	0.059	0.081	0.045	0.062	0.476	0.850	0.581	0.426
Eye corrosion	0.003	0.003	0.003	0.003	0.003	0.003	0.003	0.003	0.003
Eye irritation	0.016	0.015	0.022	0.012	0.009	0.015	0.007	0.015	0.003

#### 3.2.2. Molecular docking analysis

##### 3.2.2.1. Redocking validation

The molecular docking protocol was validated by re-docking the native co-crystallized ligands into their corresponding protein binding sites. The redocking validation was performed for ATP with CDK2 (PDB ID: 1HCK), DHT with the androgen receptor (PDB ID: 2AMA), TIV with VEGFR-2 (PDB ID: 4ASE), and 1 × 10^9^ with Bcl-2 (PDB ID: 4IEH). The superimposition of the docked and crystallographic poses is presented in Fig. 2, while the corresponding 3D and 2D interaction profiles are shown in Fig. S46. As summarized in Table 4, all redocked ligands showed RMSD values below the accepted threshold of 2.0 A, confirming the reliability of the docking protocol. The lowest RMSD value was obtained for TIV in the VEGFR-2 binding site, with an RMSD of 0.862 A, followed by DHT with an RMSD of 1.102 A. Although ATP and 1 × 10^9^ showed relatively higher RMSD values of 1.972 and 1.987 A, respectively, these values remained within the acceptable validation range. The redocked ligands also showed favorable binding energies, ranging from −6.92 to −9.44 kcal mol^−1^, together with interaction patterns involving key residues within the corresponding binding pockets. These findings indicate that the selected docking parameters were suitable for reproducing the experimentally observed ligand orientations and could therefore be reliably applied to the docking analysis of the synthesized benzofuran-furan-pyrano[2,3-*c*]pyrazole hybrids.

**Fig. 2:**
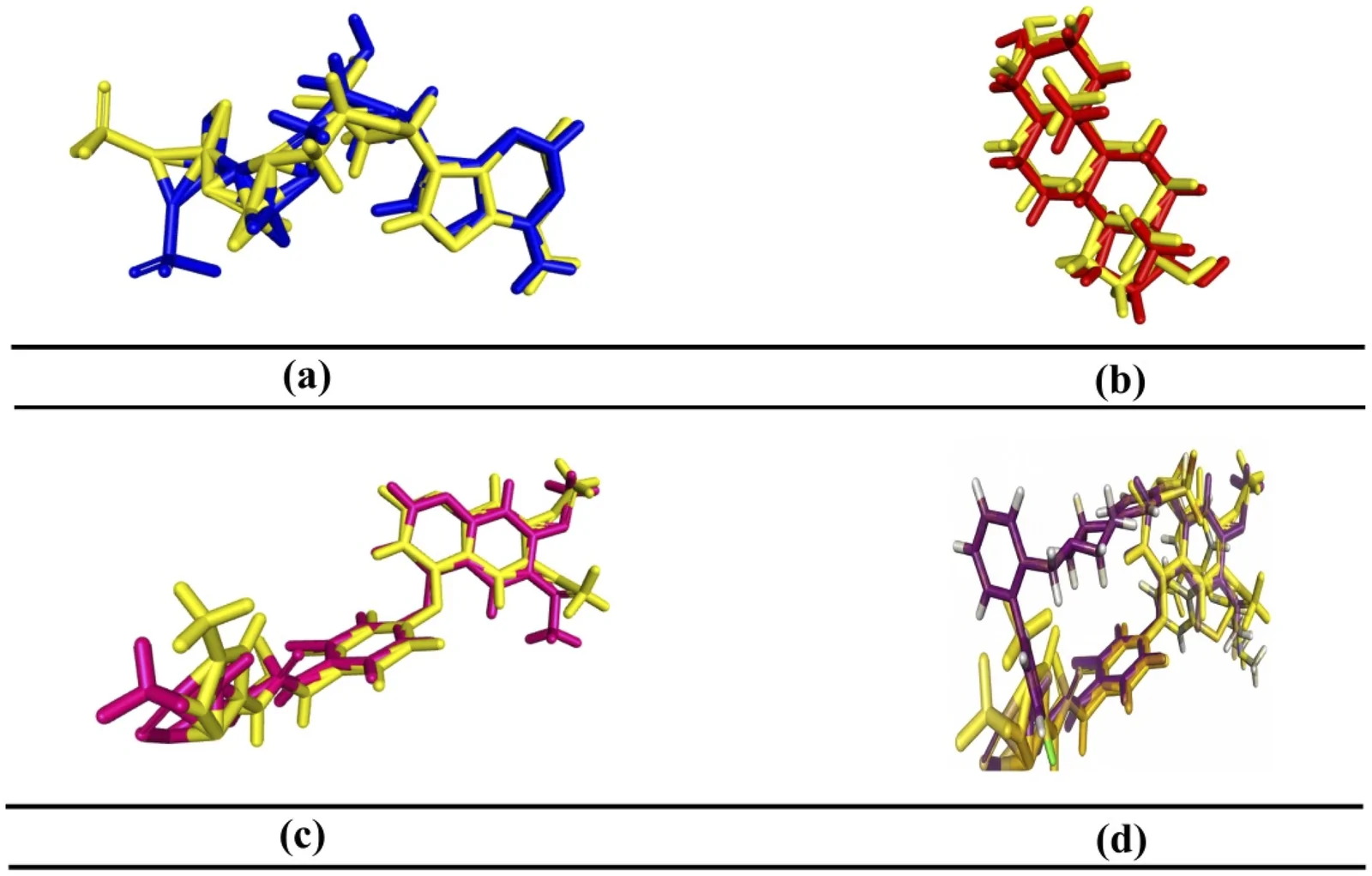
Redocking pose superimposition of co-crystallized ligands ATP/1HCK, DHT/2AMA, TIV/4ASE, and 1 × 10^9^/4IEH, where yellow represents the original crystallographic poses and the colored structures represent the redocked poses.

**Table 4:** Redocking validation results of the native co-crystallized ligands with their corresponding protein targets^a^

Protein/PDB ID	Co-crystallized ligand	Binding energy (kcal mol^−1^)	RMSD (A)	Amino acid residues
CDK2/1HCK	ATP	−9.44	1.972	GLU81, LEU83, ASP86, THR14, GLY13, GLN131, LYS33, LYS129, ILE10, VAL18, ALA31, LEU134, GLY11, GLU12, TYR15, GLY16, PHE80, PHE82, HIS84, ASN132, ASP145, THR158, VAL64, VAL164, THR165
Androgen receptor/2AMA	DHT	−8.33	1.102	ASN705, THR877, ARG752, PHE764, MET742, TRP741, LEU873, LEU704, MET745, LEU707, MET749, PHE891, LEU880, ILE899, MET895, GLY708, GLN711, LEU701, PHE876, MET780, MET787, VAL746
VEGFR-2/4ASE	TIV	−6.74	0.862	SER884, LEU886, ILE888, ILE890, ILE892, VAL898, ASN900, LEU901, VAL914, PHE918, ILE1034, LEU1036, SER1037, LYS1043, ILE1044
Bcl-2/4IEH	1 × 10^9^	−6.92	1.987	GLY104, TYR161, ARG66, TYR67, PHE63, PHE71, VAL115, MET74, ALA108, LEU96, VAL107, ARG105, ALA59, ASP62, TRP103, ASN102, ASP70, PHE112, GLU111, VAL92, GLU95, PHE157

a ATP: adenosine-5′-triphosphate; DHT: 5α-dihydrotestosterone; TIV: tivozanib; 1 × 10^9^: PDB ligand code of the co-crystallized Bcl-2 inhibitor.

##### 3.2.2.2. Docking results of synthesized compounds

The molecular docking results of the synthesized benzofuran-furan-pyrano[2,3-*c*]pyrazole hybrids (4b, 4d, 4f, 4h and 4j) against four cancer-related protein targets are summarized in Table 5. These targets included CDK2 (1HCK), androgen receptor (2AMA), VEGFR-2 (4ASE), and Bcl-2 (4IEH), which are associated with cell-cycle regulation, hormone-dependent cancer signaling, angiogenesis, and apoptosis, respectively. The docking scores were compared with the corresponding reference drugs: SEL, ENZ, TIV, and VEN. More negative binding energy values indicate stronger predicted ligand-protein affinity.^43,44^ Overall, the synthesized compounds showed promising binding affinities toward all investigated targets. Among the tested derivatives, compound 4f exhibited the strongest docking performance across the four proteins, with binding energies of −9.96 kcal mol^−1^ against 1HCK, −10.29 kcal mol^−1^ against 2AMA, −7.76 kcal mol^−1^ against 4ASE, and −8.62 kcal mol^−1^ against 4IEH. This better activity can be explained by the existence of the naphthyl substituent, which can increase hydrophobic-based and π–π interactions within the binding pockets. All the synthesized compounds showed a higher binding affinity toward CDK2 (1HCK) than the reference drug SEL (−5.11 kcal mol^−1^). The binding affinities were in the order 4f > 4b > 4d > 4h > 4j, which showed that the aromatic substitution at the 4th position was more effective in interacting with the CDK2 active site than the aliphatic pentyl analog 4j. In the case of the androgen receptor (2AMA), compounds (4b, 4d, 4f, 4h, 4j) had higher binding affinities than ENZ (−8.37 kcal mol^−1^), and 4f, once again, had the best score (−10.29 kcal mol^−1^). Compounds 4d and 4h also exhibited high and comparable binding energies (−9.58 kcal mol^−1^), indicating that *p*-tolyl and methoxyphenyl substitutions are favorable for receptor binding. Compound 4j, however, exhibited a lower affinity (−7.63 kcal mol^−1^) compared to ENZ. Compounds (4b, 4d, 4f, 4h, and 4j) obtained higher docking scores than TIV (−6.74 kcal mol^−1^) in the case of VEGFR-2 (4ASE), whereas compound 4j had the lowest affinity (−5.87 kcal mol^−1^). Compound 4f was the most active one (−7.76 kcal mol^−1^), followed by 4b and 4h. This implies that extended aromatic groups can be beneficial for the binding of VEGFR-2. For Bcl-2 (4IEH), compound 4f showed the strongest affinity (−8.62 kcal mol^−1^), exceeding the reference drug VEN (−7.05 kcal mol^−1^). Compounds 4b and 4d also demonstrated favorable binding energies, whereas 4j again displayed the weakest interaction among the synthesized derivatives. These results indicate that the synthesized hybrids, especially compound 4f, possess strong predicted binding affinity toward multiple cancer-related targets. The consistent superiority of 4f suggests that the naphthyl-containing benzofuran-furan-pyrano[2,3-*c*]pyrazole scaffold may represent a promising lead structure for further anticancer investigation. In contrast, compound 4j, bearing an aliphatic pentyl group, generally showed the weakest docking performance, highlighting the importance of aromatic substituents in improving receptor binding. Therefore, the docking study supports the potential multitarget anticancer relevance of these newly synthesized hybrids.

**Table 5:** Molecular docking binding energies (kcal mol^−1^) of benzofuran-furan-pyrano[2,3-*c*]pyrazole hybrids (4b, 4d, 4f, 4h and 4j) and reference drugs against selected cancer-related protein targets

Protein	Binding energy (kcal mol^−1^)								
	4b	4d	4f	4h	4j	SEL	ENZ	TIV	VEN
1HCK	−8.96	−8.84	−9.96	−8.30	−7.49	−5.11	—	—	—
2AMA	−9.37	−9.58	−10.29	−9.58	−7.63	—	−8.37	—	—
4ASE	−7.49	−6.87	−7.76	−7.21	−5.87	—	—	−6.74	—
4IEH	−7.96	−7.80	−8.62	−7.49	−6.58	—	—	—	−7.05

The interacting amino acid residues obtained from the molecular docking analysis are summarized in Table 6, while the corresponding key hydrogen-bonding and hydrophobic/π interaction types are provided in Table S1 of the SI. This table provides further insight into the binding behavior of the synthesized benzofuran-furan-pyrano[2,3-*c*]pyrazole hybrids (4b, 4d, 4f, 4h and 4j) within the active pockets of the selected cancer-related protein targets, namely CDK2 (1HCK), androgen receptor (2AMA), VEGFR-2 (4ASE), and Bcl-2 (4IEH). The interaction profiles were also compared with the corresponding reference drugs, SEL, ENZ, TIV, and VEN. For CDK2 (1HCK), compounds (4b, 4d, 4f, 4h and 4j) interacted with several common residues, including ILE35, ARG36, HIS125, ALA144, PHE146, GLY147, LEU148, ALA149, PHE152, VAL154, and PRO155. These residues were also observed in the interaction profile of the reference drug SEL, indicating that the synthesized compounds bind within a similar region of the CDK2 active site. The presence of aromatic residues such as PHE146 and PHE152, together with hydrophobic residues such as ILE35, LEU148, VAL154, and PRO155, may contribute to the stabilization of the ligand-protein complexes through hydrophobic and π-related interactions. In the case of the androgen receptor (2AMA), the synthesized compounds showed interactions with residues such as LEU, SER, GLU, TYR, PHE, MET, and VAL residues located within the receptor-binding pocket. Several of these residues were also involved in the binding of the reference drug ENZ, suggesting that compounds (4b, 4d, 4f, 4h and 4j) may occupy a comparable binding region. The repeated involvement of hydrophobic residues, particularly LEU, VAL, PHE, and MET, indicates that non-covalent hydrophobic interactions play an important role in stabilizing the ligands within the androgen receptor pocket. For VEGFR-2 (4ASE), the synthesized compounds interacted with key residues including ASN900, LEU901, ILE915, PHE918, ASN923, ASN1033, ILE1034, LEU1036, SER1037, LYS1043, ILE1044, and ARG1051. Many of these residues, particularly ASN900, LEU901, PHE918, LEU1036, SER1037, LYS1043, and ILE1044, were also found in the interaction pattern of the reference drug TIV. This overlap supports the ability of the synthesized hybrids to bind effectively within the VEGFR-2 active pocket and suggests a possible contribution to anti-angiogenic activity. For Bcl-2 (4IEH), compounds (4b, 4d, 4f, 4h and 4j) showed a highly consistent interaction pattern with residues such as LYS22, ARG26, PHE63, SER64, ARG68, ARG69, ASP70, ALA72, SER75, GLU111, GLY114, VAL115, and VAL118. These residues were also largely observed for the reference drug VEN, indicating that the synthesized compounds bind in a region similar to the known Bcl-2 inhibitor. The strong overlap with VEN is particularly important because Bcl-2 is associated with anti-apoptotic cancer cell survival, and interaction with these residues may support the potential apoptosis-related anticancer activity of the synthesized compounds. Table 6 demonstrates that the synthesized hybrids interact with important amino acid residues within the active sites of all selected proteins. The high similarity between the residue profiles of compounds (4b, 4d, 4f, 4h and 4j) and the reference drugs supports the reliability of their docking poses and suggests that these compounds may act through binding modes comparable to known inhibitors. Among the synthesized compounds, the aromatic derivatives, especially compound 4f, showed extensive interactions with several key residues, which is consistent with its superior binding energy values reported in Table 5. Therefore, the amino acid interaction analysis further supports the predicted interactions of these benzofuran-furan-pyrano[2,3-*c*]pyrazole hybrids with multiple cancer-related targets.

**Table 6:** Interacting amino acid residues of benzofuran-furan-pyrano[2,3-*c*]pyrazole hybrids (4b, 4d, 4f, 4h and 4j) and reference drugs within the binding pockets of selected cancer-related protein targets

Protein	Compound	Interacted amino acid residues
1HCK	4b	VAL17, LYS34, ILE35, ARG36, HIS125, ALA144, PHE146, GLY147, LEU148, ALA149, ARG150, PHE152, GLY153, VAL154, PRO155
	4d	ILE35, ARG36, HIS125, ARG126, ALA144, PHE146, GLY147, LEU148, ALA149, ARG150, PHE152
	4f	VAL17, ILE35, ARG36, HIS125, ARG126, ALA144, PHE146, GLY147, LEU148, ALA149, ARG150, PHE152, GLY153, VAL154, PRO155
	4h	LYS34, ILE35, ARG36, PHE80, LEU124, HIS125, ARG126, ALA144, PHE146, GLY147, LEU148, ALA149, PHE152, VAL154, ARG169
	4j	VAL17, LYS34, ILE35, ARG36, HIS125, ARG126, ALA144, PHE146, GLY147, LEU148, ALA149, PHE152, VAL154, PRO155
	SEL	HIS125, ARG126, LEU128, ALA144, PHE146, GLY147, LEU148, ALA149, PHE152, GLY153, VAL154, PRO155, ARG169
2AMA	4b	LEU707, MET745, VAL746, GLY750, SER753, LEU762, TYR763, ALA765, LEU768, PHE770, SER778, CYS784, MET787, LEU873
	4d	LEU701, SER703, GLU706, LEU707, GLY708, GLN711, TRP741, MET742, MET745, SER778, LEU873, PHE876, PHE878, LEU880, LEU881, PHE891, ILE899
	4f	LEU700, LEU701, SER703, LEU707, GLY708, MET742, MET745, VAL746, GLY750, SER753, LEU762, TYR763, PHE770, SER778, MET787
	4h	LEU701, SER703, GLU706, LEU707, GLY708, GLU709, TRP741, MET742, MET745, VAL746, LEU762, LEU768, PHE770, MET787, LEU873, MET895
	4j	LEU700, LEU701, SER702, SER703, GLU706, LEU707, GLY708, GLN711, MET745, LEU762, TYR763, ALA765, LEU768, PHE770
	ENZ	LEU701, SER702, SER703, GLU706, LEU707, GLY708, GLU709, VAL746, ALA748, GLY750, SER753, LEU762, TYR763, LEU768, PHE770, MET787
4ASE	4b	ASN900, ILE915, PHE918, PHE921, GLY922, ASN923, LEU924, ARG1032, ASN1033, ILE1034, LEU1036, SER1037, LYS1043, ILE1044, ARG1051
	4d	VAL865, VAL898, ASN900, LEU901, ILE915, PHE918, GLY922, ASN923, LEU924, ARG1032, ASN1033, ILE1034, LEU1036, LYS1043, ILE1044, ARG1051
	4f	ILE849, VAL865, VAL867, ASN900, LEU902, ILE915, PHE918, LEU1036, SER1037, LYS1043
	4h	VAL867, ASN900, LEU901, LEU902, VAL914, ILE915, HIS1026, ASN1033, ILE1034, LEU1036, SER1037, LYS1043, ILE1044
	4j	VAL898, ASN900, LEU902, PHE918, ASN1033, ILE1034, LEU1036, VAL1042, LYS1043, ILE1044
	TIV	SER884, LEU886, ILE888, ILE890, ILE892, VAL898, ASN900, LEU901, VAL914, PHE918, ILE1034, LEU1036, SER1037, LYS1043, ILE1044
4IEH	4b	LYS22, ARG26, PHE63, SER64, ARG65, ARG68, ASP70, ALA72, SER75, GLU111, GLY114, VAL115, VAL118
	4d	LYS22, GLN25, ARG26, PHE63, SER64, ASP70, ALA72, SER75, GLU111, VAL115, VAL118, GLU119, ASN122
	4f	ARG26, PHE63, SER64, ASP70, ALA72, SER75, GLU111, PHE112, VAL115, VAL118, GLU119
	4h	LYS22, GLN25, ARG26, PHE63, SER64, ARG68, ARG69, ASP70, ALA72, SER75, GLU111, GLY114, VAL115, VAL118, GLU119
	4j	LYS22, ARG26, PHE63, SER64, ARG68, ARG69, ASP70, ALA72, SER75, GLU111, GLY114, VAL115, VAL118
	VEN	LYS22, GLN25, ARG26, TYR28, PHE63, SER64, ARG68, ARG69, ASP70, ALA72, SER75, LEU96, PHE109, GLU111, PHE112, GLY114, VAL115, VAL118, ASN122

To further characterize these binding modes, the key conventional hydrogen-bonding and hydrophobic/π interactions were analyzed in detail. The principal interaction types, including conventional hydrogen bonds, π–π, π–σ, π-alkyl, and other relevant non-covalent contacts, are summarized in Table S1 of the SI. The corresponding 3D and 2D interaction profiles are presented in Fig. S47–S50.

The binding modes of the synthesized benzofuran-furan-pyrano[2,3-*c*]pyrazole hybrids were further examined through 3D docking poses and 2D interaction diagrams. Fig. 3 presents the detailed interaction profile of the most active derivative, compound 4f, with CDK2 (1HCK), together with the reference inhibitor SEL. The complete docking interaction maps of all synthesized compounds and the corresponding reference drugs are provided in the SI as Fig. S47–S50 for 1HCK, 2AMA, 4ASE, and 4IEH, respectively. As shown in Fig. 3, compound 4f was well accommodated within the CDK2 active pocket and adopted a stable binding orientation. The interaction pattern involved several important residues, including VAL17, ILE35, ARG36, HIS125, ARG126, ALA144, PHE146, GLY147, LEU148, ALA149, ARG150, PHE152, GLY153, VAL154, and PRO155. The extended naphthyl-type aromatic moiety of 4f seems to make a significant contribution to the stabilization of the complexes, especially with aromatic and hydrophobic residues such as PHE146, PHE152, VAL154, and PRO155. This interaction profile positively contributes to the excellent docking behavior of 4f reported in Table 5. The entire profiles of CDK2 interaction presented in Fig. S47 reveal that compounds (4b, 4d, 4f, 4h and 4j) possess a similar binding space to that of SEL. Their interactions with residues were shared with ILE35, ARG36, HIS125, ALA144, PHE146, LEU148, ALA149, PHE152, VAL154, and PRO155, indicating a similar binding mode in the CDK2 pocket. The synthesized compounds, particularly the aromatic derivatives, notably 4f, had a greater number of interactions than the aliphatic derivative 4j, indicating that the aromatic substitution plays a positive role in enhancing ligand accommodation. In the case of the androgen receptor model 2AMA, the interaction maps in Fig. S48 reveal that the synthesized compounds were located in the ligand-binding domain, where they interacted chiefly with hydrophobic residues, such as LEU, VAL, MET, PHE, and TYR residues. This interaction is relevant to the stabilization of the ligands within the androgen receptor pocket. The interaction profile of compound 4f was especially ideal, which can be explained by its longer aromatic surface and better hydrophobic complementarity with the receptor cavity. The docking poses in the case of VEGFR-2 (4ASE) (Fig. S48) showed that the synthesized hybrids were inserted into the active pocket and interacted with residues that were also identified in the binding profile of the reference drug TIV. Important residues included ASN900, LEU901, ILE915, PHE918, ASN923, ASN1033, ILE1034, LEU1036, SER1037, LYS1043, ILE1044, and ARG1051. These interactions suggest that the benzofuran-furan-pyrano[2,3-*c*]pyrazole scaffold can establish stable contacts within the VEGFR-2 binding site. The binding interactions with Bcl-2 4IEH are shown in Fig. S50. Compounds (4b, 4d, 4f, 4h and 4j) interacted with several residues within the Bcl-2 pocket, including LYS22, ARG26, PHE63, SER64, ARG68, ARG69, ASP70, ALA72, SER75, GLU111, GLY114, VAL115, and VAL118. The fact that the interaction profile of the synthesized compounds overlaps with that of the reference inhibitor VEN shows that these hybrids bind at a similar area of the Bcl-2 active site. The docking score results are summarized in Table 5, and the amino acid interaction data are tabulated in Table 6, which are supported by 3D and 2D docking visualizations. The synthesized hybrids, particularly compound 4f, showed favorable predicted binding poses within the active pockets of CDK2, AR, VEGFR-2, and Bcl-2. These computational findings support further experimental investigation of the naphthyl-substituted benzofuran-furan-pyrano[2,3-*c*]pyrazole scaffold.

**Fig. 3:**
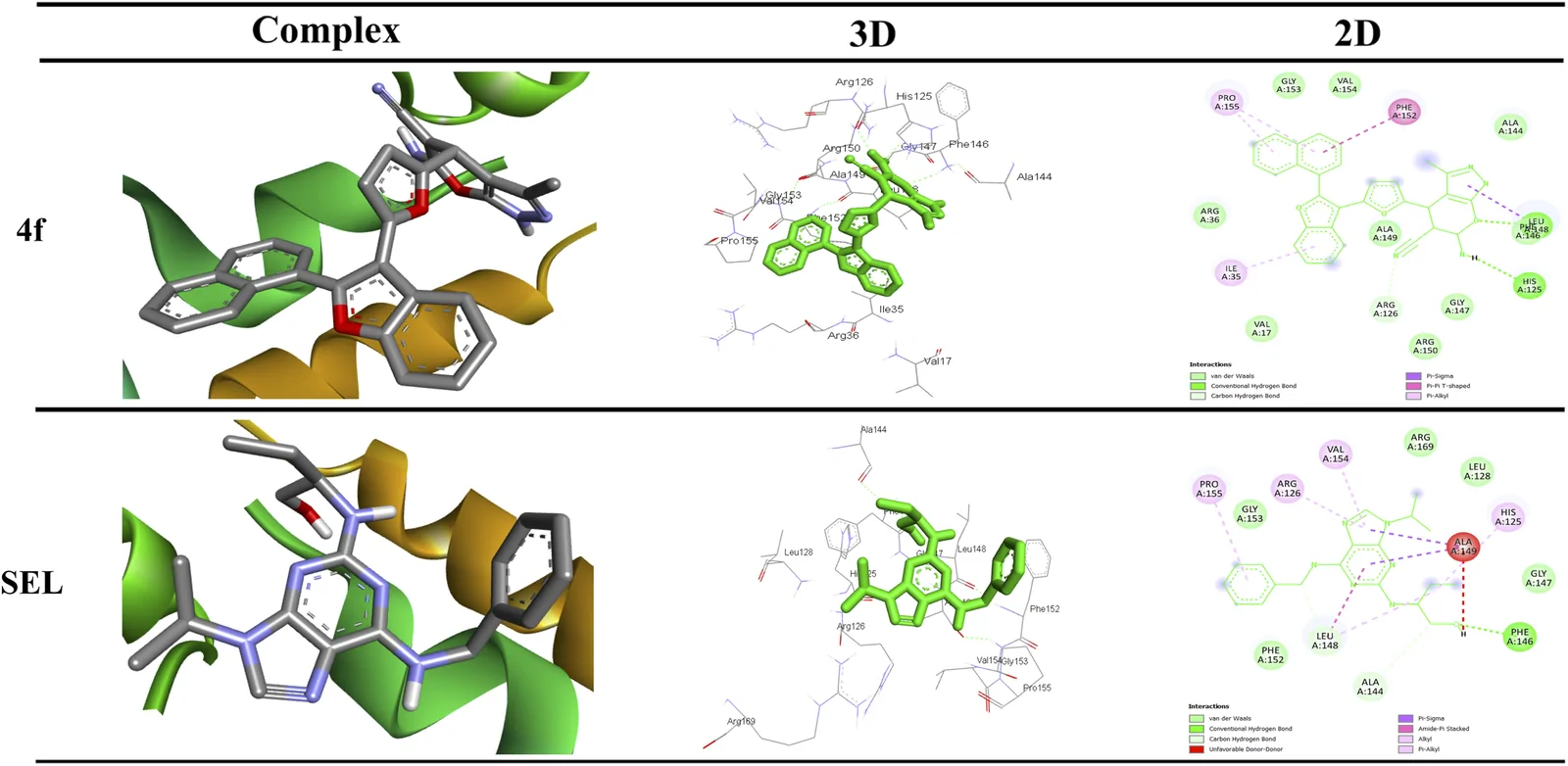
3D binding pose and 2D interaction diagram of compound 4f within the active pocket of CDK2 (PDB ID: 1HCK) compared with the reference inhibitor SEL.

### 3.3. *In vitro* anticancer activity

#### 3.3.1 Cytotoxicity

The cytotoxic profiles of benzofuran-furan-pyrano[2,3-*c*]pyrazole hybrids (4b, 4d, 4f, 4h and 4j) were evaluated against human lung (A549), colon (Caco-2), prostate (LNCaP), and breast (MDA-MB-231) cancer cell lines, as well as healthy human umbilical vein endothelial cells (HUVEC), using the MTT cell viability assay. Cells were treated with concentrations ranging from 7.8 to 500 µM for 24 h, and paclitaxel (34.4 µM) was employed as a positive control.

Derivatives 4b and 4f did not exhibit significant cytotoxic effects across the tested cancer cell lines (Fig. 4). In contrast, compounds 4d, 4h and 4j demonstrated dose-dependent cytotoxicity in A549 cells, with EC_50_ values of 274.37, 179.83, and 32.71 µM, respectively. In Caco-2 cells, the corresponding EC_50_ values for compounds 4d, 4h and 4j were 117.04, 83.50, and 20.49 µM, respectively. Similarly, in LNCaP cells, EC_50_ values of 219.58, 102.73, and 20.84 µM were obtained for these compounds. In the MDA-MB-231 cell line, only compounds 4h and 4j displayed measurable cytotoxicity, with EC_50_ values of 88.26 and 53.14 µM, respectively. In HUVEC cells, derivatives 4b, 4d, 4f and 4h did not induce significant cytotoxicity within the tested concentration range, whereas compound 4j exhibited an EC_50_ value of 45.13 µM (Fig. 4).

**Fig. 4:**
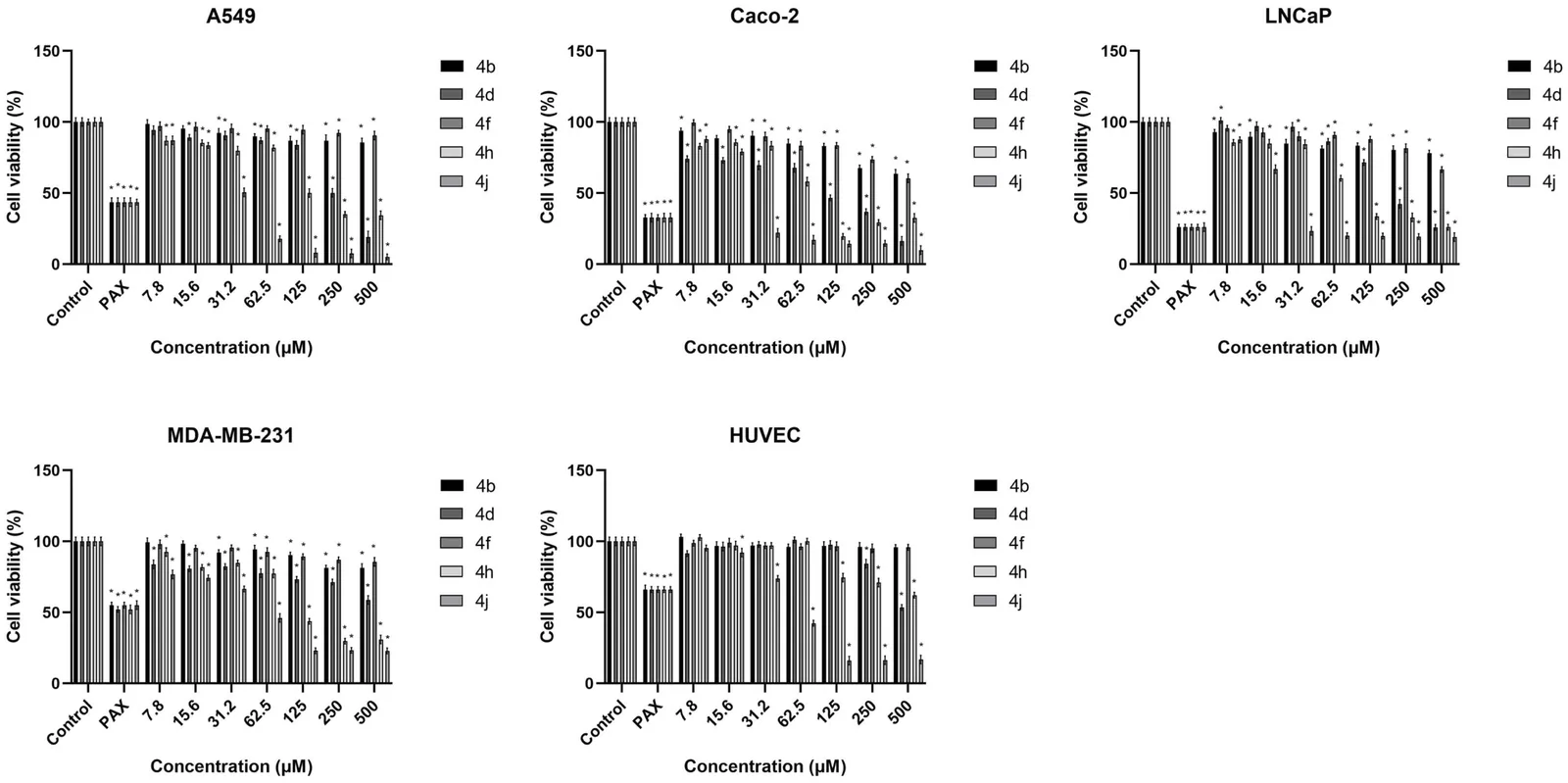
Cytotoxic activity of benzofuran-furan-pyrano[2,3-*c*]pyrazole hybrid derivatives (4b, 4d, 4f, 4h and 4j) in A549, Caco-2, LNCaP, MDA-MB-231, and HUVEC cells. Cell viability was assessed by the MTT assay after 24 h of treatment (7.8–500 µM). *p < 0.05 was considered statistically significant.

Compounds 4d, 4h and 4j were selected for subsequent mechanistic studies because they exhibited clear concentration-dependent cytotoxicity and yielded measurable EC_50_ values in the tested cancer cell lines.

This study demonstrates that newly synthesized benzofuran-furan-pyrano[2,3-*c*]pyrazole hybrids, particularly compounds 4d, 4h and 4j, exhibit pronounced and selective anticancer activity across multiple human cancer cell lines (A549, Caco-2, LNCaP, and MDA-MB-231), while largely preserving the viability of non-malignant endothelial cells. MTT-based cytotoxicity profiling revealed that the hybrid compounds displayed significant cytotoxic effects in A549 (EC_50_ = 32.71–274.37), Caco-2 (EC_50_ = 20.49–117.04), LNCaP (EC_50_ = 20.84–219.58), and MDA-MB-231 (EC_50_ = 53.14–88.26) cells (Fig. 4). A preliminary structure–activity relationship (SAR) analysis revealed a clear influence of the substituent attached to the benzofuran-containing framework on cytotoxic activity. The unsubstituted phenyl derivative 4b showed no significant cytotoxicity, whereas introduction of an electron-donating methyl group in 4d resulted in measurable activity against A549, Caco-2, and LNCaP cells. Replacement of the methyl group with the stronger electron-donating methoxy substituent in 4h further improved cytotoxic potency and extended measurable activity to MDA-MB-231 cells. In contrast, the bulkier naphthyl-containing derivative 4f remained largely inactive, suggesting that increased steric bulk may be unfavorable for biological activity in this series. Notably, replacement of the aryl substituent with the more flexible hydrophobic alkyl chain in 4j produced the greatest cytotoxic potency across all tested cancer cell lines. Collectively, these observations suggest that moderate electron-donating substitution and substituent flexibility favor cytotoxic activity, whereas excessive aromatic bulk may be detrimental. However, given the limited number of derivatives, these SAR trends should be considered preliminary.

Previous studies have reported that various benzofuran derivatives, particularly imidazopyridine-benzofuran analogues containing electron-donating substituents at the 4-position of the phenyl ring and halogenated modifications, exhibit strong cytotoxic activity against several cancer cell lines, including lung (A549), breast (MCF-7), leukemia (HL-60), colon (SW480), epithelial ovarian (A2780), and colorectal adenocarcinoma (Colo-205) cells.^7^ Similarly, several benzofuran-trimethoxybenzamide derivatives have shown potent antimitotic activity across multiple cancer cell models. Within this series, compound 6g emerged as the most active derivative, with IC_50_ values of 3.01 µM for MDA-MB-231, 5.20 µM for HCT-116, 9.13 µM for HT-29, and 11.09 µM for HeLa cells.^45^ In another study, hydrazone-derived pyrazole-benzofuran compounds exhibited marked antiproliferative activity against various human cancer cell lines (HeLa, A549, and HT29), suppressing cell growth through multiple molecular targets. XTT-based analyses confirmed dose-dependent antiproliferative effects for all compounds, with compound 3d showing notable potency in A549 cells with an IC_50_ value of 0.28 µM and a high selectivity index.^46,47^ Anwar and colleagues demonstrated that nanoparticle-formulated benzofuran-pyrazole derivatives (BZP-NP) exerted strong cytotoxic effects against MCF-7 and MDA-MB-231 breast cancer cells while maintaining a favorable safety profile in healthy breast cells.^48^ Similarly, halogenated 3-amino-1-aryl-8-methoxy-pyran carbonitrile derivatives, particularly compounds 4m, 4n, and 4j, exhibited potent antiproliferative activity against MCF-7, HCT-116, and HepG-2 cells, with minimal toxicity toward normal cell lines.^49^ In addition, 4*H*-pyran derivatives were reported to significantly suppress HCT-116 cell proliferation, with compounds 4d and 4k showing IC_50_ values of 75.1 and 85.88 µM, respectively.^50^

Overall, compound 4j exhibited the highest cytotoxic potency in A549, Caco-2, LNCaP and MDA-MB-231 cell lines at low micromolar concentrations while maintaining a higher EC_50_ value in HUVEC cells, indicating a degree of selectivity toward cancer cells. Compounds 4h and 4d also demonstrated greater cytotoxic activity in cancer cell lines compared to healthy endothelial cells, whereas compounds 4b and 4f remained largely inactive under the experimental conditions.

#### 3.3.2. Flow cytometric analysis of apoptosis induced by benzofuran-furan-pyrano[2,3-*c*]pyrazole derivatives

Apoptotic responses to benzofuran-furan-pyrano[2,3-*c*]pyrazole hybrid derivatives were quantified by flow cytometry following 24 h of exposure at their respective EC_50_ concentrations in A549, Caco-2, LNCaP, and MDA-MB-231 cancer cell lines. Paclitaxel (11.4 µM) was used as the positive control (Fig. 5).

**Fig. 5:**
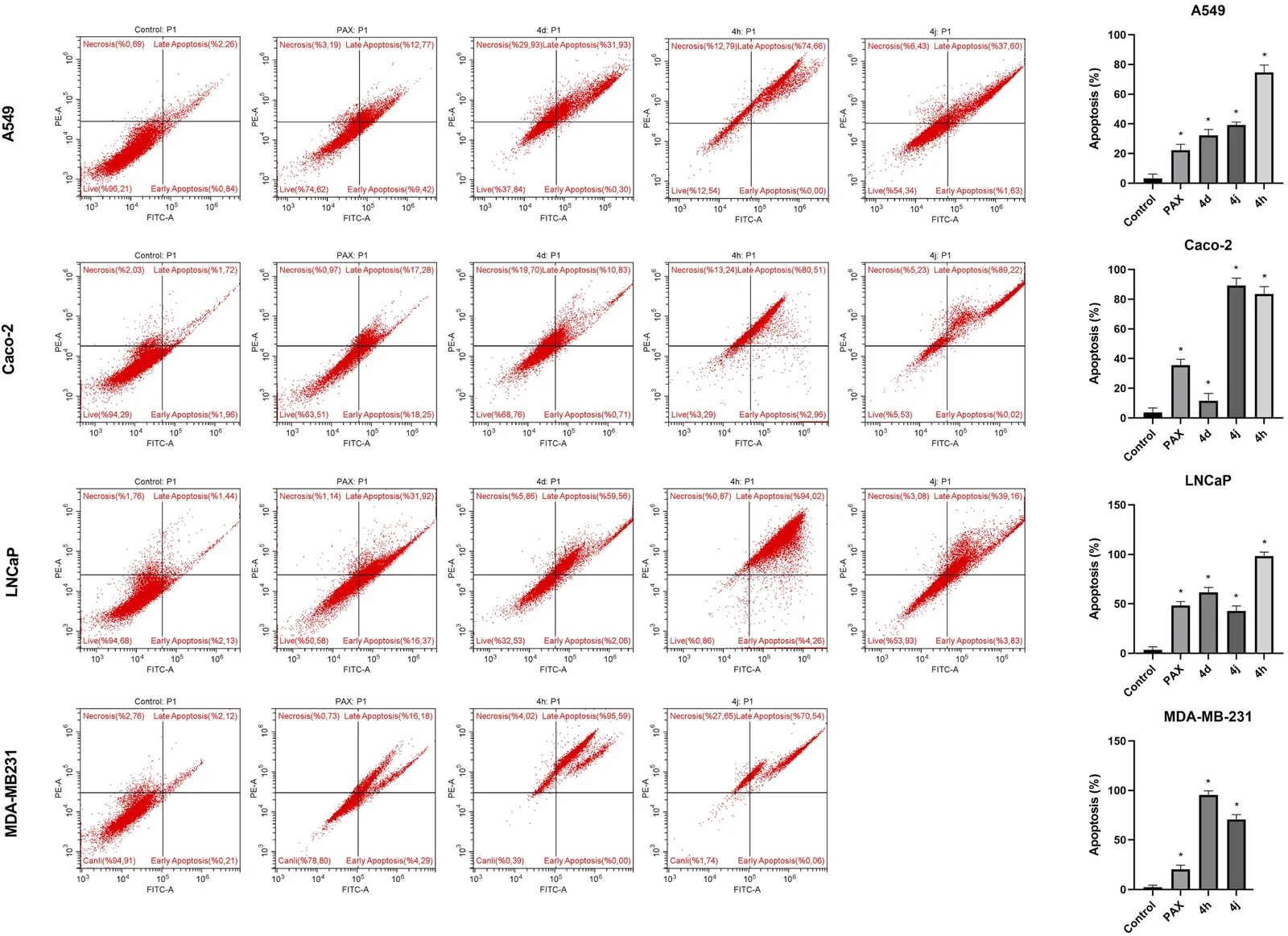
Apoptotic effects of benzofuran-furan-pyrano-pyrazole hybrids (4d, 4h and 4j) in A549, Caco-2, LNCaP, and MDA-MB-231 cells. Apoptosis was quantified by flow cytometry after 24 h of treatment at EC_50_ concentrations; paclitaxel (11.4 µM) was used as the positive control. *p < 0.05 indicates statistical significance.

In A549 cells, basal apoptosis in the negative control was 3.10%, comprising 0.84% early and 2.26% late apoptosis, while paclitaxel increased apoptotic cells to 22.19%, including 9.42% early and 12.77% late apoptosis. Treatment with compounds 4d, 4h and 4j significantly elevated total apoptotic populations to 32.23%, 74.66%, and 39.23%, respectively. The corresponding early apoptotic populations were 0.30%, 0.00%, and 1.63%, while the late apoptotic populations were 31.93%, 74.66%, and 37.60%, respectively. The corresponding necrotic fractions were 29.93%, 12.79%, and 6.43%.

In Caco-2 cells, the negative control exhibited 3.68% total apoptosis, consisting of 1.96% early and 1.72% late apoptosis, whereas paclitaxel induced 35.53% total apoptosis, including 18.25% early and 17.28% late apoptosis. Compounds 4d, 4h and 4j resulted in total apoptotic rates of 11.54%, 83.47%, and 89.24%, respectively. The corresponding early apoptotic populations were 0.71%, 2.96%, and 0.02%, while the late apoptotic populations were 10.83%, 80.51%, and 89.22%, respectively. The necrotic fractions were 19.70%, 13.24%, and 5.23%, respectively (Fig. 5).

In LNCaP cells, basal apoptosis was 3.57%, comprising 2.13% early and 1.44% late apoptosis, and paclitaxel increased total apoptosis to 48.29%, including 16.37% early and 31.92% late apoptosis. Exposure to compounds 4d, 4h and 4j led to pronounced total apoptotic responses of 61.62%, 98.28%, and 42.99%, respectively. The corresponding early apoptotic populations were 2.06%, 4.26%, and 3.83%, while the late apoptotic populations were 59.56%, 94.02%, and 39.16%, respectively. Necrosis remained minimal at 5.86%, 0.87%, and 3.08%, respectively (Fig. 5). In MDA-MB-231 cells, untreated controls showed 2.33% total apoptosis, consisting of 0.21% early and 2.12% late apoptosis, and paclitaxel induced 20.47% total apoptosis, including 4.29% early and 16.18% late apoptosis. Compounds 4h and 4j markedly enhanced total apoptosis to 95.59% and 70.62%, respectively. Early apoptotic populations were 0.00% and 0.08%, whereas late apoptotic populations reached 95.59% and 70.54%, respectively. However, compound 4j was associated with a higher necrotic fraction (27.65%) compared with compound 4h (4.02%) (Fig. 5).

In conclusion, these findings demonstrate that derivatives 4d, 4h and 4j elicit significant apoptotic responses across multiple cancer cell lines. The increases in total apoptosis were predominantly associated with accumulation in the late apoptotic population, particularly following treatment with compounds 4h and 4j.

#### 3.3.3. qRT-PCR analysis of apoptosis-related genes

To elucidate the molecular mechanisms underlying the apoptotic effects of benzofuran-furan-pyrano[2,3-*c*]pyrazole hybrids, quantitative real-time PCR (qRT-PCR) analysis was performed in A549, Caco-2, LNCaP, and MDA-MB-231 cancer cell lines following 24 h of exposure to compounds 4d, 4h and 4j at their respective EC_50_ concentrations. Total RNA was isolated, reverse-transcribed into cDNA, and the mRNA expression levels of apoptosis-related genes (BCL2, BAX, CASP3, CASP8, CASP9, and TP53) were quantified and normalized to the housekeeping gene GAPDH. Gene expression levels were evaluated relative to untreated controls.

In A549 cells, treatment with 4d, 4h and 4j resulted in marked downregulation of the anti-apoptotic gene BCL2 to 34.70%, 28.05%, and 20.17% of control, respectively. This was accompanied by significant upregulation of BAX (2.21-, 1.89-, and 2.05-fold), CASP3 (1.84-, 2.01-, and 2.02-fold), CASP8 (1.93-, 2.10-, and 1.96-fold), CASP9 (2.00-, 2.21-, and 2.20-fold), and TP53 (2.05-, 2.02-, and 2.07-fold). In Caco-2 cells, BCL2 expression was similarly suppressed to 30.74%, 22.62%, and 7.98%, for 4d, 4h and 4j, respectively, while BAX (2.09-, 2.43-, and 3.43-fold), CASP3 (1.99-, 2.16-, and 2.64-fold), CASP8 (2.04-, 2.18-, and 2.54-fold), CASP9 (1.84-, 2.32-, and 2.28-fold), and TP53 (3.56-, 4.46-, and 4.65-fold) were significantly upregulated (Fig. 6). In LNCaP cells, exposure to 4d, 4h and 4j induced a reduction in BCL2 expression to 3.07%, 8.29%, and 12.94%, respectively, alongside pronounced increases in BAX (1.96-, 1.61-, and 4.74-fold), CASP3 (2.80-, 2.10-, and 7.21-fold), CASP8 (2.43-, 1.77-, and 6.91-fold), CASP9 (2.33-, 2.15-, and 5.73-fold), and TP53 (2.48-, 2.21-, and 5.63-fold). In MDA-MB-231 cells, compounds 4h and 4j significantly downregulated BCL2 to 6.37% and 6.04%, respectively, and concomitantly increased the expression of BAX (1.88- and 2.05-fold), CASP3 (1.67- and 3.61-fold), CASP8 (1.89- and 1.87-fold), CASP9 (2.04- and 1.74-fold), and TP53 (2.00- and 2.65-fold) (Fig. 6).

**Fig. 6:**
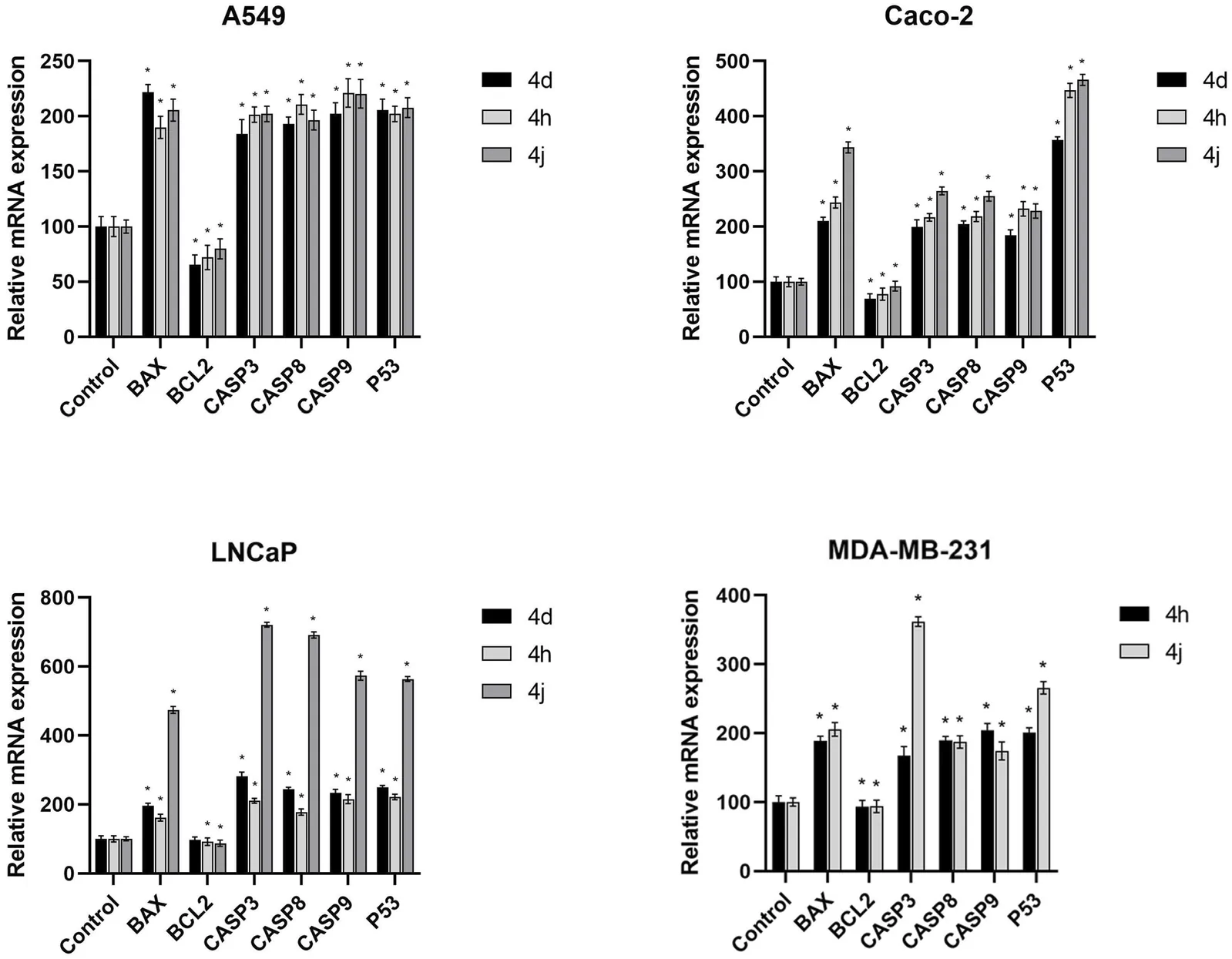
Relative mRNA expression of apoptosis-related genes in A549, Caco-2, LNCaP, and MDA-MB-231 cells following 24 h treatment with benzofuran-furan-pyrano-pyrazole hybrids (4d, 4h and 4j) at EC_50_ concentrations, as determined by qRT-PCR. Gene expression was normalized to GAPDH and expressed relative to untreated controls. *p < 0.05 indicates statistical significance.

Flow cytometric apoptosis analysis demonstrated that benzofuran-furan-pyrano[2,3-*c*]pyrazole hybrids 4d, 4h and 4j significantly increased apoptotic cell populations compared with the negative controls, although their effects relative to paclitaxel varied depending on the compound and cell line (Fig. 5). The increase in total apoptosis was predominantly associated with accumulation in the late apoptotic population. These findings are consistent with the reduction in cell viability observed in the MTT assay, suggesting that apoptosis contributed to the cytotoxic effects of the compounds. These findings were consistent with the reduced cell viability observed in the MTT assay, although their quantitative relationship requires further investigation.

At the molecular level, qRT-PCR analyses revealed coordinated changes in the expression of genes associated with both intrinsic and extrinsic apoptotic pathways (Fig. 6). Downregulation of the anti-apoptotic gene BCL2, together with upregulation of BAX, CASP3, CASP8, CASP9, and TP53, is consistent with the possible involvement of death receptor-mediated signaling and mitochondrial apoptosis. The consistent induction of TP53 may suggest the involvement of tumor suppressor-mediated stress response pathways, potentially amplifying apoptotic signaling and reinforcing cell cycle regulatory mechanisms. Similarly, benzofuran derivatives have been shown to disrupt mitochondrial membrane integrity, leading to cytochrome c release and subsequent caspase-3/7 activation, which are hallmarks of classical intrinsic apoptosis.^51^ Benzofuran-2-acetic ester derivatives have also been reported to induce apoptosis in both ER-positive and triple-negative breast cancer cells, supported by caspase activation, increased Bax/Bcl-2 ratio, and DNA fragmentation.^52,53^ Benzofuran-based chalcone analogues were shown to induce apoptosis in HCC1806, HeLa, and A549 cells, with compound 4g additionally inhibiting the VEGFR-2 signaling pathway.^54^ Furthermore, newly synthesized benzofuran derivatives were reported to activate both intrinsic and extrinsic apoptotic pathways in leukemia cell lines through upregulation of pro-apoptotic genes.^55^ Similar increases in p53, p21, and Bax gene expression have also been documented.^56^ Pyrazole-benzofuran compounds were further shown to enhance apoptotic signaling through caspase activation and modulation of PI3K/AKT/mTOR pathways.^47^ As a result, these transcriptional profiles are consistent with the possible involvement of both intrinsic and extrinsic apoptotic pathways in cells treated with derivatives 4d, 4h and 4j, as evidenced by coordinated suppression of BCL2 and induction of BAX, caspases, and TP53, thereby supporting the cytotoxicity and flow cytometry findings. However, protein-level validation is required to confirm activation of these pathways.

#### 3.3.4. ROS analysis

The effects of benzofuran-furan-pyrano[2,3-*c*]pyrazole hybrids (4d, 4h and 4j) on intracellular reactive oxygen species (ROS) levels were investigated in A549, Caco-2, LNCaP, and MDA-MB-231 cancer cell lines following 24 h incubation at their respective EC_50_ concentrations. A 100 µM concentration of TBHP was used as the positive control.

Flow cytometric analysis revealed that treatment of A549 cells with compounds 4d, 4h and 4j induced substantial ROS generation, resulting in ROS-positive cell populations of 99.86%, 100%, and 97.29%, respectively. Relative to the untreated control (24.60%), these values corresponded to approximately 4.05-, 4.06-, and 3.95-fold increases (Fig. 7). Similarly, in Caco-2 cells, compounds 4d, 4h and 4j elicited near-maximal ROS production, with ROS-positive fractions of 99.69%, 99.96%, and 99.98%, respectively. These responses represented approximately 5.56–5.57-fold elevations compared with the untreated control (17.92%) (Fig. 7).

**Fig. 7:**
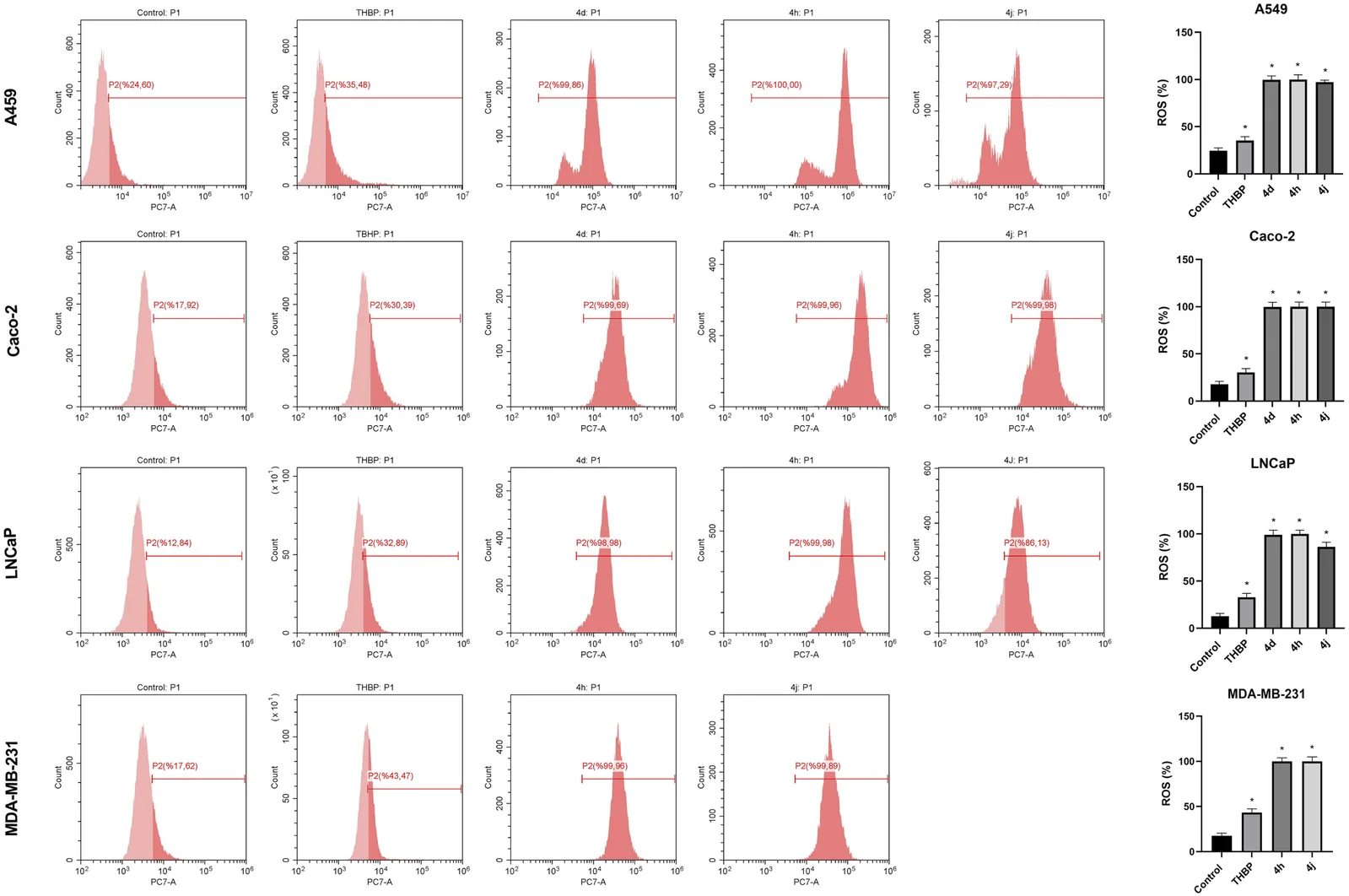
Effects of 4d, 4h and 4j hybrids on intracellular ROS production in A549, Caco-2, LNCaP, and MDA-MB-231 cancer cell lines following 24 h treatment at EC_50_ concentrations, as determined by flow cytometry. TBHP (100 µM) was used as the positive control. Significant difference from negative control (*p < 0.05).

In LNCaP cells, exposure to derivatives 4d, 4h and 4j markedly enhanced ROS levels (98.98%, 99.98%, and 86.13%), corresponding to approximately 7.70-, 7.78-, and 6.70-fold increases over the control (12.84%). In the MDA-MB-231 cell line, compounds 4h and 4j strongly promoted ROS formation, with ROS-positive populations of 99.96% and 99.89%, corresponding to approximately 5.66- and 5.67-fold increases relative to the control (17.62%) (Fig. 7).

Overall, the pronounced ROS induction by benzofuran-furan-pyrano[2,3-*c*]pyrazole hybrids (4d, 4h and 4j) suggests the activation of oxidative stress-mediated apoptotic pathways in the treated cancer cell lines.

#### 3.3.5. Mitochondrial membrane potential

The effects of benzofuran-furan-pyrano[2,3-*c*]pyrazole hybrids (4d, 4h and 4j) on mitochondrial membrane potential (MMP) were evaluated in A549, Caco-2, LNCaP, and MDA-MB-231 cell lines. 150 µM CCCP was used as the positive control.

In A549 cells, the negative control exhibited an MMP of 90.88%, whereas CCCP treatment reduced MMP to 76.46%. Treatment with compounds 4j, 4d and 4h resulted in MMP values of 68.47%, 81.08%, and 80.24%, corresponding to MMP losses of 31.52%, 18.91%, and 19.76%, respectively, indicating marked mitochondrial depolarization relative to the control (Fig. 8).

**Fig. 8:**
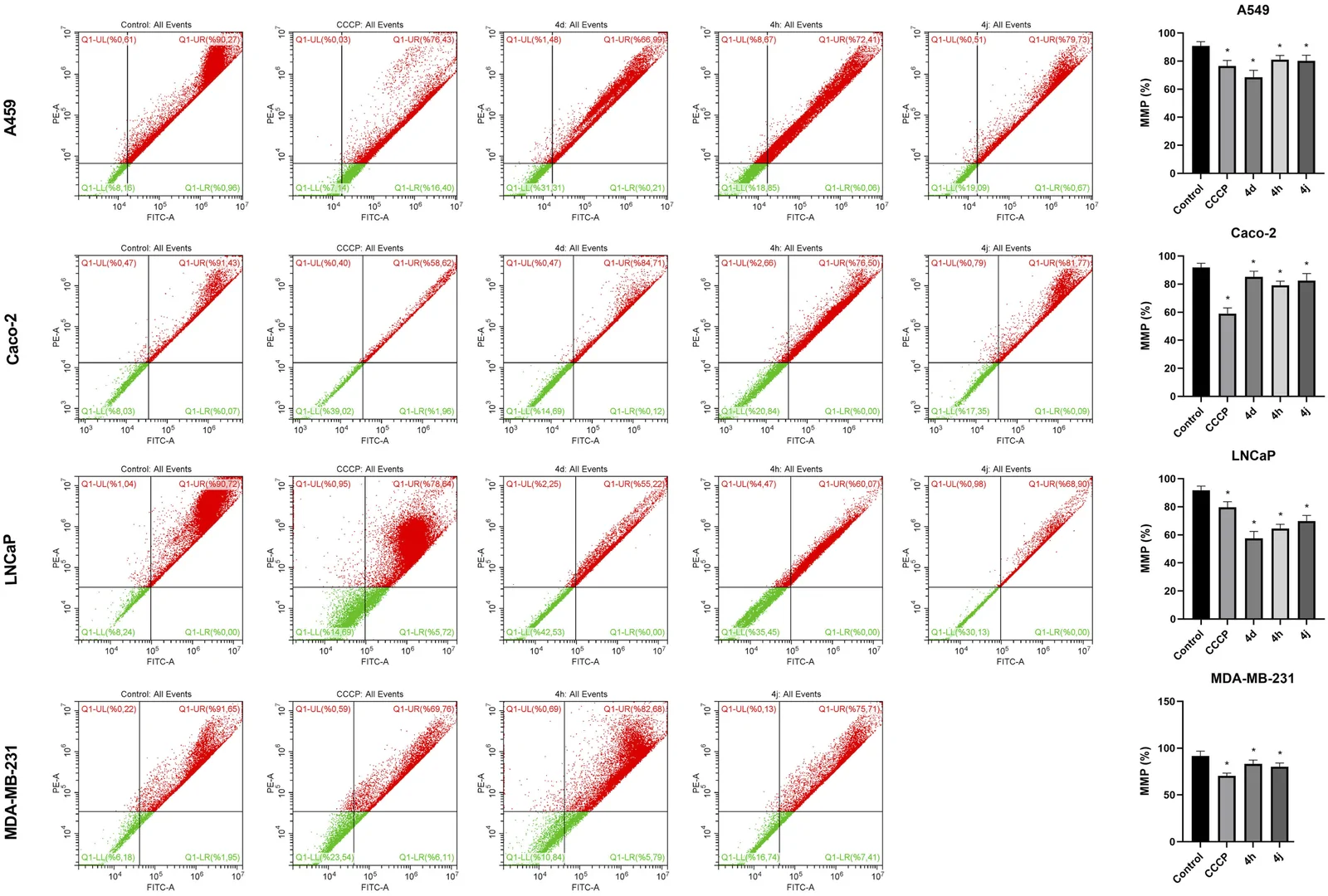
Effects of 4d, 4h and 4j hybrids on mitochondrial membrane potential (MMP) in A549, Caco-2, LNCaP, and MDA-MB-231 cancer cell lines following 24 h treatment at EC_50_ concentrations. CCCP was used as the positive control, significant difference from negative control (*p < 0.05).

In Caco-2 cells, the control group showed an MMP of 91.90%, which decreased to 59.02% following CCCP exposure. Treatment with compounds 4d, 4h and 4j yielded MMP values of 85.18%, 79.16%, and 82.56%, corresponding to losses of 14.81%, 20.84%, and 17.44%, respectively (Fig. 8). In LNCaP cells, the control group exhibited an MMP of 91.76%, while CCCP reduced MMP to 79.59%. Exposure to compounds 4d, 4h and 4j significantly decreased MMP to 57.47%, 64.54%, and 69.88%, resulting in losses of 42.53%, 35.45%, and 30.13%, respectively (Fig. 8). In MDA-MB-231 cells, the negative control showed an MMP of 91.87%, which decreased to 70.35% following CCCP treatment. Compounds 4h and 4j reduced MMP to 83.37% and 75.84%, corresponding to losses of 16.63% and 24.15%, respectively (Fig. 8).

These findings indicate that benzofuran-furan-pyrano[2,3-*c*]pyrazole hybrids (4d, 4h and 4j) induce pronounced mitochondrial dysfunction in multiple cancer cell lines.

The observed increase in intracellular ROS generation across all tested cancer cell lines was associated with the cellular responses induced by the compounds. The pronounced ROS accumulation induced by compounds 4d, 4h and 4j, together with the significant loss of mitochondrial membrane potential (MMP), is consistent with the involvement of oxidative stress and mitochondrial dysfunction (Fig. 7 and 8). Mitochondrial depolarization is commonly associated with intrinsic apoptosis, oxidative stress, caspase activation, and downstream apoptotic execution. These findings indicate an association between ROS accumulation, mitochondrial dysfunction, and apoptosis but do not establish a causal relationship. Similarly, benzofuran derivatives have been shown to increase ROS production in human chondrosarcoma cells, accompanied by decreased MMP, indicating activation of mitochondria-mediated apoptotic pathways.^57^ Pyrazole derivatives have also been reported to elevate intracellular ROS levels in MDA-MB-468, A-549, H226, and H460 cancer cells.^58,59^ Moreover, pyrazole-based benzofuran derivatives were shown to disrupt mitochondrial integrity and activate intrinsic apoptosis in MCF-7 cells.^60^

#### 3.3.6. Cell cycle analysis

The effects of hybrids 4d, 4h and 4j on cell cycle progression were analyzed by flow cytometry following 24 h treatment at their respective EC_50_ concentrations in cancer cell lines.

In A549 cells, the control group exhibited 17.08% of cells in the G1 phase, 4.99% in the S phase, and 61.75% in the G2 phase. Treatment with compound 4d resulted in a distribution of 84.84% in G1, 12.35% in S, and 3.12% in G2. Compound 4h yielded 43.15% in G1, 11.68% in S, and 39.82% in G2, whereas compound 4j produced 72.16% in G1, 22.61% in S, and 5.83% in G2. These results indicate that compounds 4d, 4h and 4j predominantly induced cell cycle arrest in the G1 and S phases relative to the control (Fig. 9).

**Fig. 9:**
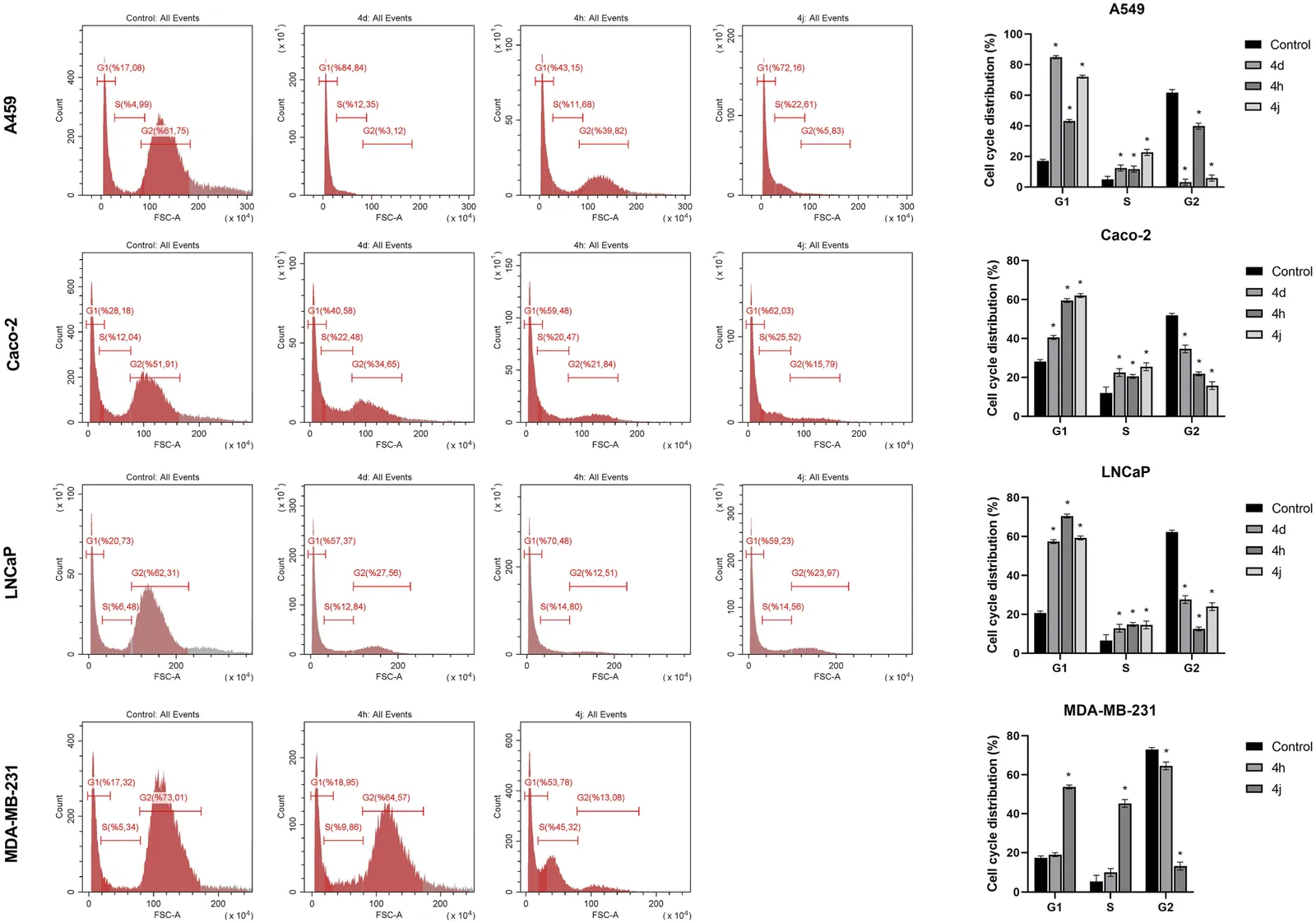
Effects of 4d, 4h and 4j hybrids on cell cycle distribution in A549, Caco-2, LNCaP, and MDA-MB-231 cancer cell lines following 24 h treatment at EC_50_ concentrations, as determined by flow cytometry. Cell populations in the G1, S, and G2 phases were quantified and normalized to the untreated control. Significant difference from negative control (*p < 0.05).

In Caco-2 cells, the control group displayed 28.18% of cells in G1, 12.04% in S, and 51.91% in G2. Following treatment with compound 4d, the distribution shifted to 40.58% in G1, 22.48% in S, and 34.65% in G2. Compound 4h resulted in 59.48% in G1, 20.47% in S, and 21.84% in G2, while compound 4j produced 62.03% in G1, 25.52% in S, and 15.79% in G2. These findings demonstrate an accumulation of cells in the G1 and S phases following treatment (Fig. 9).

In LNCaP cells, the control group exhibited 20.73% of cells in G1, 6.48% in S, and 62.31% in G2. Treatment with compound 4d yielded 57.37% in G1, 12.84% in S, and 27.56% in G2. Compound 4h resulted in 70.48% in G1, 14.80% in S, and 12.51% in G2, whereas compound 4j produced 59.23% in G1, 14.56% in S, and 23.97% in G2. These data indicate that the compounds induced cell cycle arrest primarily in the G1 and S phases (Fig. 9).

In the MDA-MB-231 cell line, the control group showed 17.32% of cells in G1, 5.34% in S, and 73.01% in G2. Treatment with compound 4h resulted in 18.95% in G1, 9.86% in S, and 64.57% in G2, indicating no significant deviation from the control. In contrast, compound 4j markedly altered the cell cycle distribution, yielding 53.78% in G1, 45.32% in S, and 13.08% in G2, consistent with pronounced arrest in the G1 and S phases (Fig. 9).

Cell cycle analysis revealed that treatment with compounds 4d, 4h and 4j induced accumulation of cancer cell populations at the G1/S checkpoint (Fig. 9), consistent with disruption of cell-cycle progression at the G1/S transition. However, because cell-cycle regulatory proteins were not examined, the molecular mechanisms underlying this effect remain to be determined. Previous reports have shown that certain benzofuran derivatives exert anticancer effects through disruption of cell cycle progression. These compounds have been shown to interfere with mitotic spindle formation, resulting in G2/M arrest and subsequent inhibition of proliferation. For instance, benzofuran hybrid derivatives were reported to induce G2/M arrest in A549, MCF-7, Caco-2, Ishikawa, and HepG2 cells by inhibiting polymerization, concurrently triggering apoptosis.^8,17^ Pyrazole derivatives have similarly been shown to arrest the cell cycle at S and G2 phases in MDA-MB-468, MCF-7, and HeLa cells while simultaneously inducing apoptosis.^58,61,62^ Likewise, pyran derivatives have demonstrated significant arrest at G0/G1, S, and G2/M phases in MCF-7, A549, and HCT116 cells.^63^

#### 3.3.7. Anti-invasive effects

The effects of benzofuran-furan-pyrano[2,3-*c*]pyrazole hybrids (4d, 4h and 4j) on the invasive potential of cancer cells were evaluated using a Matrigel-based invasion circle assay. Human lung (A549), colon (Caco-2), prostate (LNCaP), and breast (MDA-MB-231) cancer cell lines were treated with compounds 4d, 4h and 4j at their respective EC_50_ concentrations for 24 h, and the proportion of invading cells was quantitatively determined. Invasion in the control groups was defined as 100%.

In A549 cells, invasion rates were reduced to 20.59%, 34.72%, and 1.01% following treatment with compounds 4d, 4h and 4j, respectively. In Caco-2 cells, invasion levels decreased to 46.98%, 17.33%, and 29.77% for 4d, 4h and 4j, respectively. Similarly, in LNCaP cells, invasion rates were reduced to 26.90%, 15.28%, and 19.47%, respectively, indicating significant inhibition of cellular invasion. In MDA-MB-231 cells, compounds 4h and 4j decreased invasion to 54.56% and 13.12%, respectively, relative to the control (Fig. 10).

**Fig. 10:**
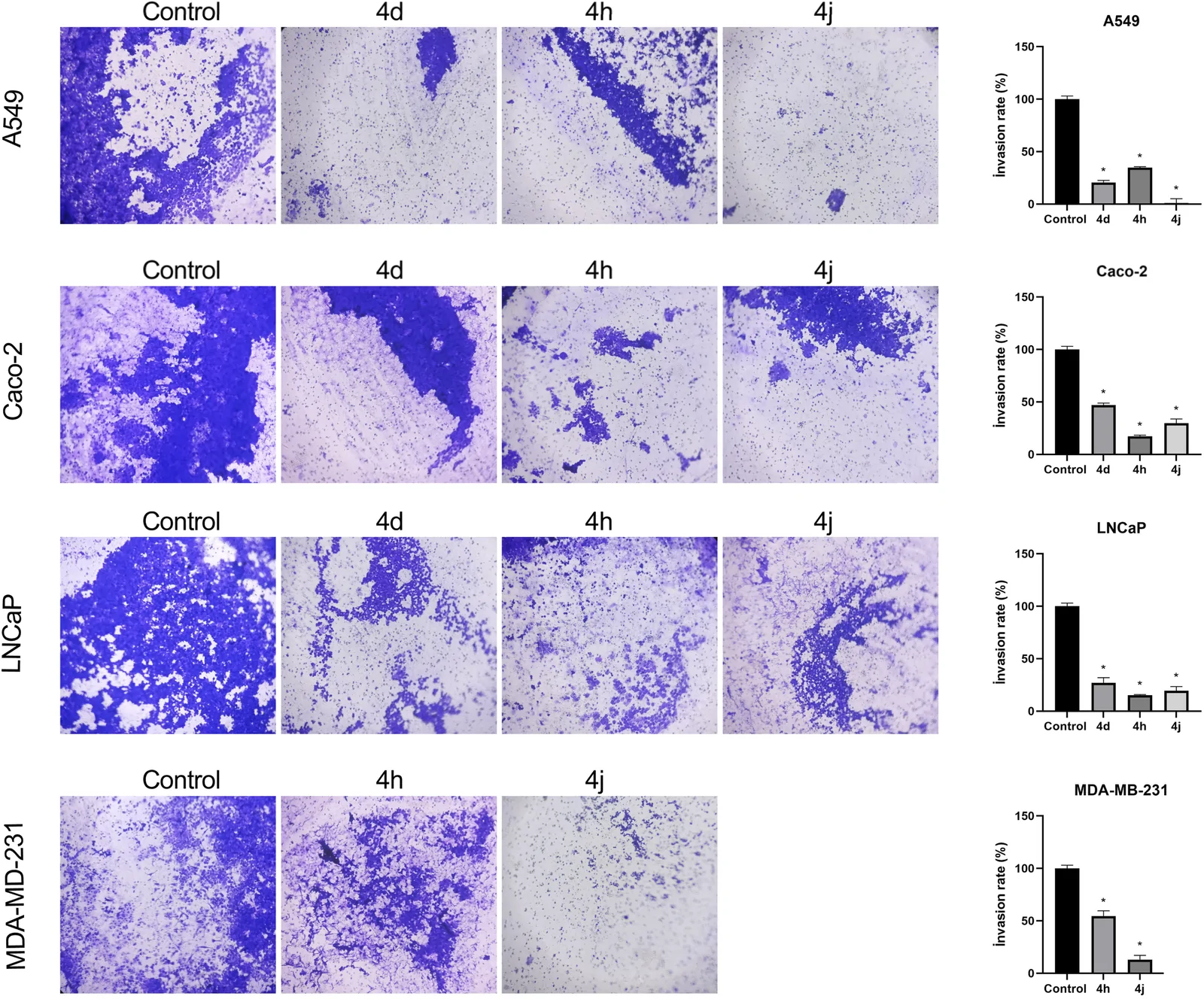
Invasion of A549, Caco-2, LNCaP, and MDA-MB-231 cells following 24 h treatment with 4d, 4h and 4j at EC_50_ concentrations, as assessed by a Matrigel-based invasion assay. Significant difference from negative control (*p < 0.05).

Collectively, these findings demonstrate that benzofuran-furan-pyrano-pyrazole hybrids (4d, 4h and 4j) markedly suppress the invasive behavior of multiple cancer cell lines, highlighting their potential anti-metastatic properties.

#### 3.3.8. Cell migration

The effects of hybrids 4d, 4h and 4j on cancer cell migration were evaluated using a wound-healing assay. A549, Caco-2, LNCaP, and MDA-MB-231 cells were monitored at 0, 24, and 48 h following treatment with the selected compounds. Wound areas were quantified using ImageJ software, and wound closure was calculated relative to the initial wound area measured at 0 h.

In A549 cells, untreated control cultures exhibited progressive wound closure, reaching approximately 76.61% at 48 h. In contrast, treatment with compounds 4d, 4h and 4j reduced wound closure to approximately 22.37%, 25.56%, and 9.55%, respectively (Fig. 11). Among the tested compounds, 4j exhibited the strongest inhibitory effect on wound closure.

**Fig. 11:**
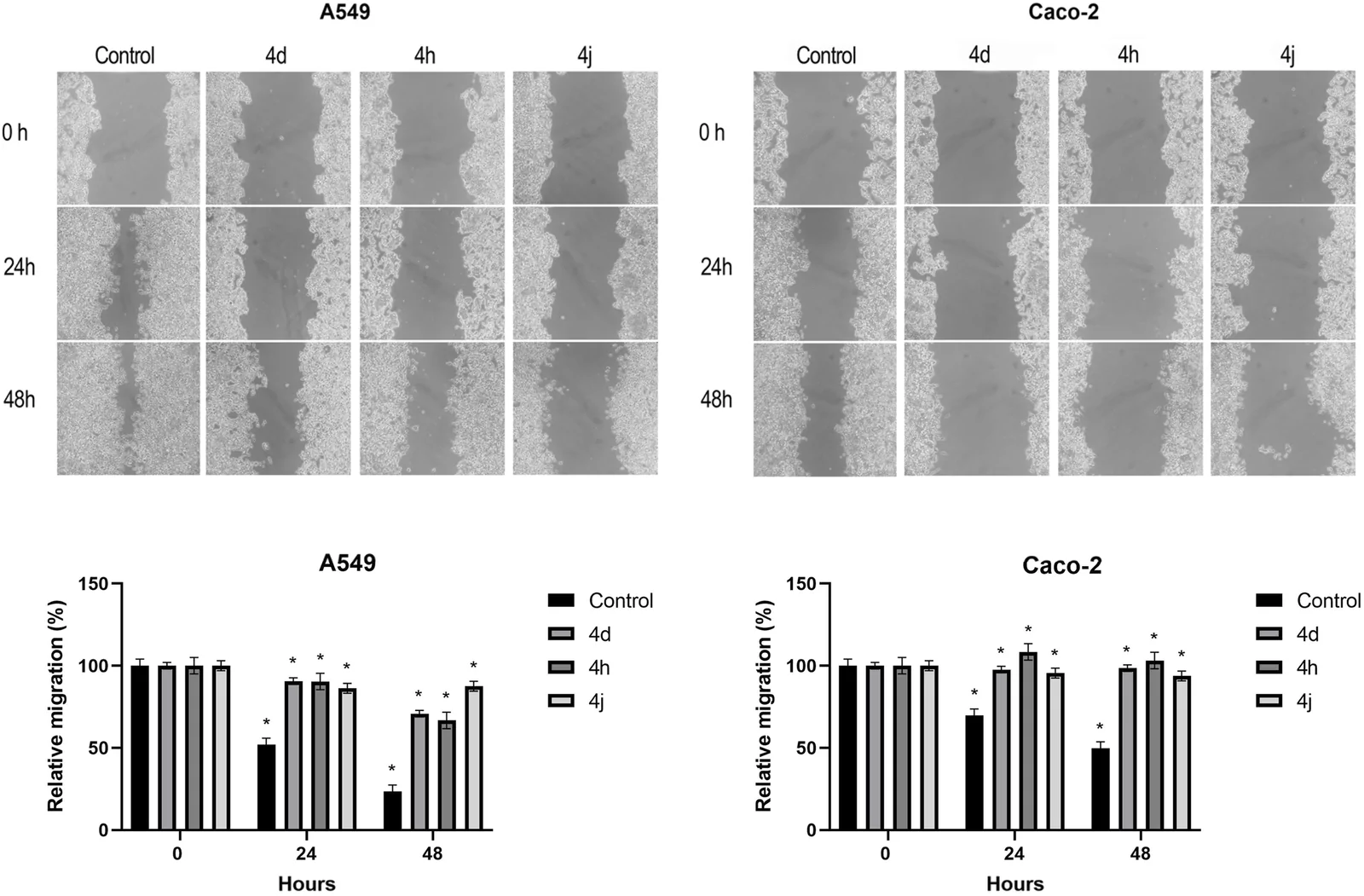
Effects of 4d, 4h and 4j hybrids on cancer cell migration in A549 and Caco-2 cell lines as determined by a wound healing assay at 0, 24, and 48 h. Wound closure was quantified using ImageJ software and normalized to the untreated control. *p < 0.05, indicating a statistically significant difference compared to the 0-h time point of the control group.

In Caco-2 cells, untreated control cultures exhibited approximately 50.31% wound closure at 48 h, whereas treatment with compounds 4d, 4h and 4j reduced wound closure to approximately 0.72%, −1.63%, and 3.14%, respectively (Fig. 11).

Similarly, untreated LNCaP cells exhibited approximately 60.46% wound closure at 48 h, while the corresponding values following treatment with compounds 4d, 4h and 4j were approximately 16.00%, 12.01%, and −4.61%, respectively (Fig. 12). The negative wound-closure values observed for 4h in Caco-2 cells and 4j in LNCaP cells indicate a slight increase in wound area relative to the initial measurement rather than migration inhibition exceeding 100%.

**Fig. 12:**
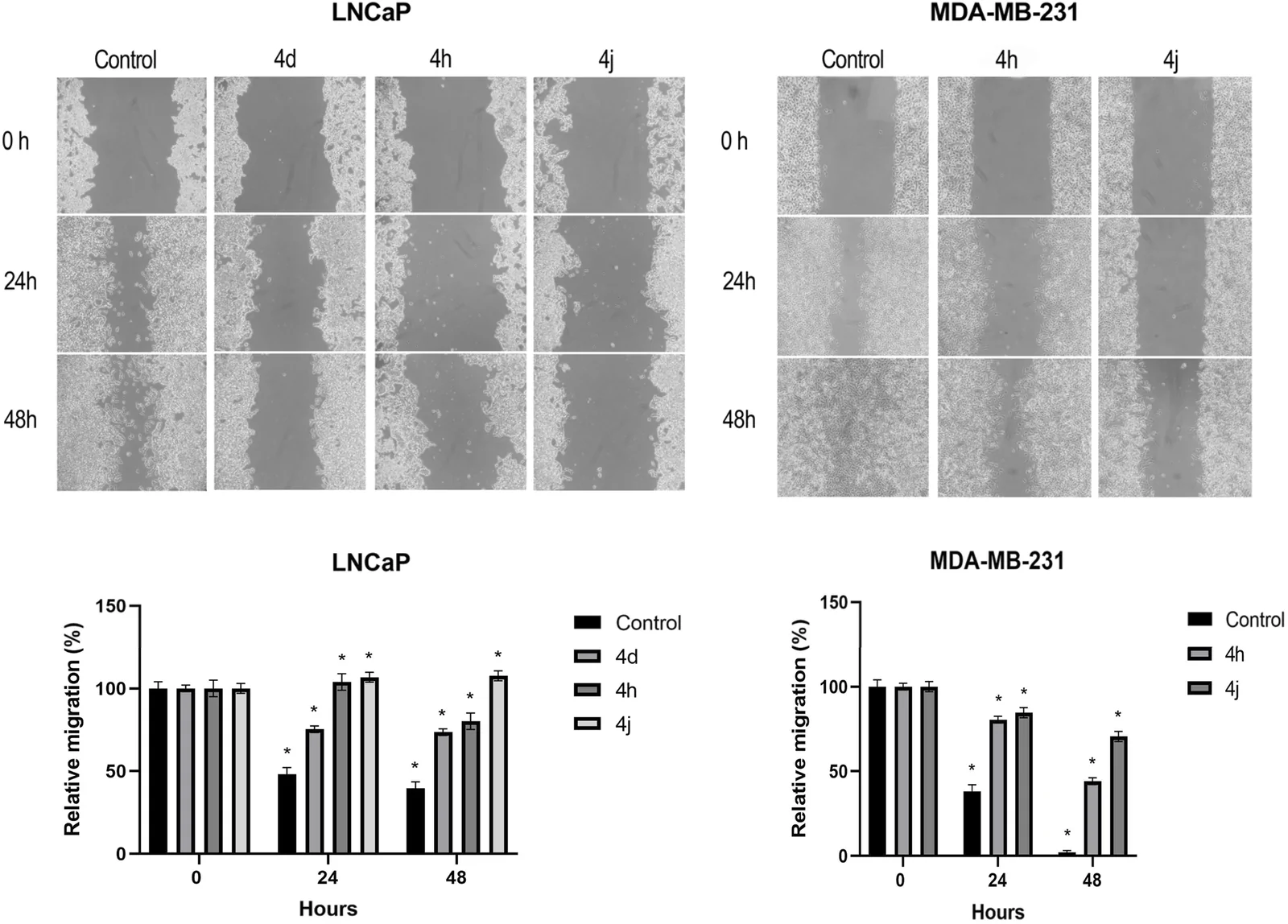
Effects of 4d, 4h and 4j hybrids on cancer cell migration in LNCaP, and MDA-MB-231 cell lines as determined by a wound healing assay at 0, 24, and 48 h. Wound closure was quantified using ImageJ software and normalized to the untreated control. *p < 0.05, indicating a statistically significant difference compared to the 0-h time point of the control group.

In MDA-MB-231 cells, untreated control cultures demonstrated rapid wound closure, reaching approximately 97.77% at 48 h. In contrast, treatment with compounds 4h and 4j reduced wound closure to approximately 54.55% and 28.81%, respectively (Fig. 12), with compound 4j exhibiting the stronger inhibitory effect.

These findings demonstrate that the benzofuran-furan-pyrano[2,3-*c*]pyrazole hybrids (4d, 4h and 4j), markedly suppressed wound closure in multiple cancer cell lines, indicating their potential anti-migratory activity.

#### 3.3.9. Colony formation

The effects of benzofuran-furan-pyrano[2,3-*c*]pyrazole hybrids (4d, 4h and 4j) on clonogenic survival were assessed using a colony formation assay following 24 h of exposure at their respective EC_50_ concentrations. Colony numbers were normalized to the untreated control, which was defined as 100%.

In A549 cells, compounds 4d, 4h and 4j resulted in near-complete suppression of clonogenic growth, reducing colony formation by 99.89%, 98.98%, and 99.70%, respectively. Similarly, in Caco-2 cells, substantial inhibition was observed, with reductions of 97.35% (4d), 98.20% (4h), and 89.84% (4j). In LNCaP cells, treatment with 4d, 4h and 4j decreased colony formation by 97.24%, 91.48%, and 86.21%, respectively (Fig. 13).

**Fig. 13:**
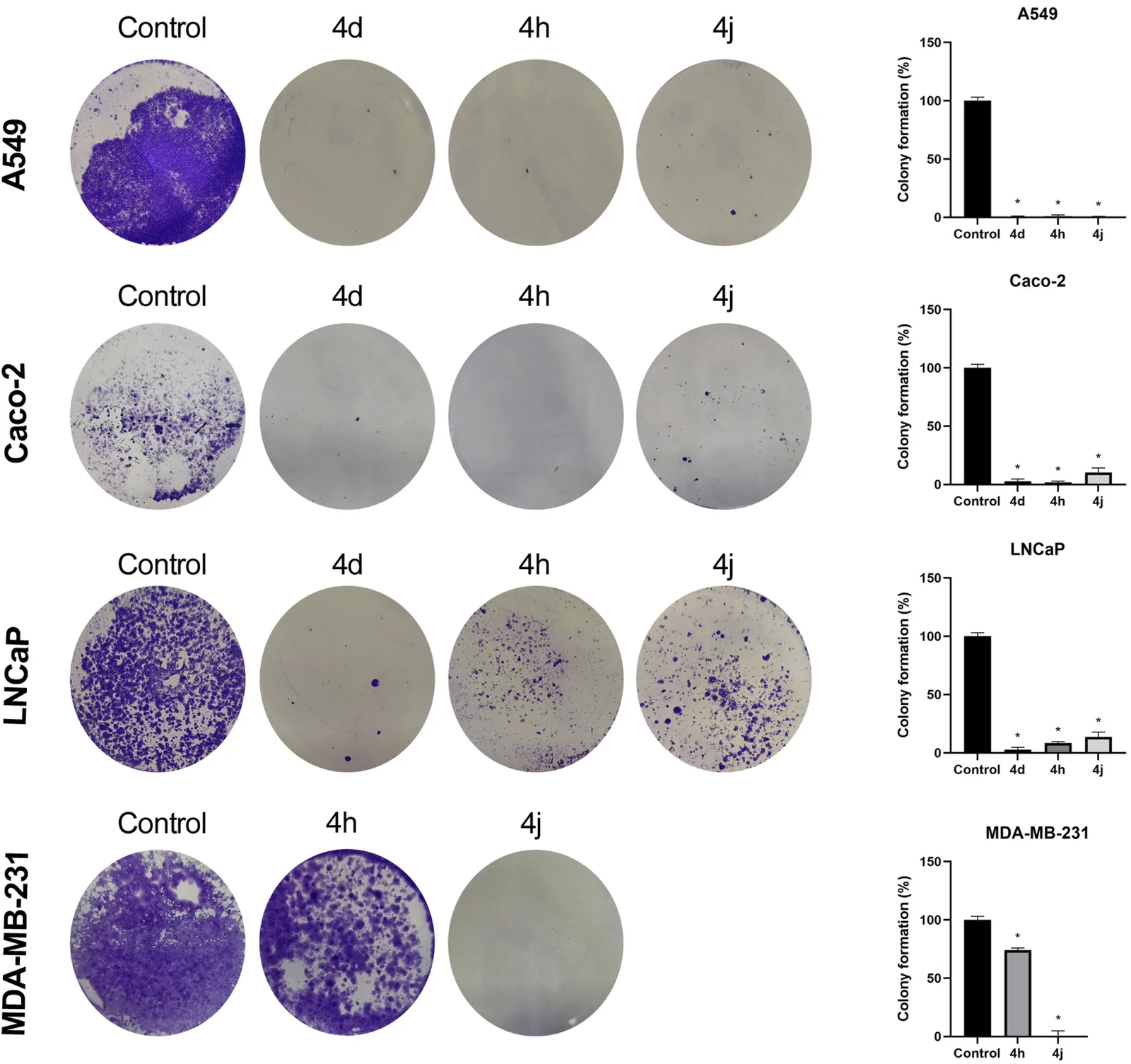
Effects of 4d, 4h and 4j derivatives on clonogenic survival of A549, Caco-2, LNCaP, and MDA-MB-231 cancer cell lines following 24 h exposure at their respective EC_50_ concentrations. Significant difference from negative control (*p < 0.05).

In the MDA-MB-231 breast cancer cell line, compounds 4h and 4j exhibited differential effects: 4h induced a modest reduction in clonogenic survival (22.36%), whereas 4j produced a pronounced inhibitory effect (99.20%) (Fig. 13).

In summary, these results indicate that derivatives 4d, 4h and 4j significantly inhibited the clonogenic potential of multiple cancer cell lines. Although the mechanistic analyses were limited to 24 h, the marked reduction in colony formation following a single 24 h exposure suggests a longer-lasting impairment of clonogenic capacity. However, further time-course and recovery studies are required to determine whether the observed cellular and molecular effects are sustained or reversible.

Treatment of cancer cells with EC_50_ concentrations of benzofuran-furan-pyrano[2,3-*c*]pyrazole hybrids (4d, 4h and 4j) significantly suppressed cell invasion, inhibited migration, and markedly reduced colony formation (Fig. 10–13). However, because the migration and invasion assays were performed at EC_50_ concentrations, a contribution from reduced cell viability or proliferation cannot be excluded. The effects of the benzofuran derivative 2-(4-benzyloxy-3-methoxyphenyl)-5-(carbethoxyethylene)-7-methoxy-benzofuran (BMBF) on hepatocellular carcinoma cells have previously been investigated, demonstrating a significant reduction in invasion capacity in p53-mutant (Y220C) Huh7 cells under non-cytotoxic concentrations. This anti-invasive activity was associated with suppression of the epithelial–mesenchymal transition (EMT), characterized by increased E-cadherin expression and decreased expression of vimentin, Slug, and MMP-9. Furthermore, BMBF was shown to inhibit the FAK/AKT signaling pathway *via* downregulation of integrin α7 and to suppress invasion through p53-dependent mechanisms.^64^ In another study, two 2-benzofuran-substituted chalcone derivatives combined with tamoxifen significantly inhibited colony formation, cell migration, and Matrigel invasion in MCF-7 cells.^65^ Benzofuran-isatin hybrid compounds were reported to reduce invasion in HT29 and SW620 cells in a dose-dependent manner,^66^ while benzofuran-indole hybrids suppressed EGFR signaling and reduced migration in PC9 and A549 cells.^67^ Similarly, pyran derivatives significantly reduced colony-forming capacity in MCF-7, HCT116, and A549 cells.^63^ Benzofuran-artesunate hybrid derivatives were also shown to inhibit migration and colony formation in Caco-2, Ishikawa, and HepG2 cells.^17^

## 4. Conclusion

In summary, the synthesized benzofuran-furan-pyrano[2,3-*c*]pyrazole hybrids exhibited significant anticancer activity against multiple human cancer cell lines while showing lower toxicity toward non-malignant cells. These compounds effectively inhibited cancer cell proliferation, migration, invasion, and colony formation. Mechanistic studies demonstrated that their anticancer effects were associated with G1/S phase cell cycle arrest, ROS-mediated mitochondrial dysfunction, loss of mitochondrial membrane potential, and induction of apoptosis through the coordinated regulation of intrinsic and extrinsic apoptotic pathways. In addition, molecular docking suggested potential interactions with multiple cancer-related targets, while ADMET analyses supported favorable drug-like properties. Collectively, these findings identify benzofuran-furan-pyrano[2,3-*c*]pyrazole hybrids as promising candidates with multifaceted *in vitro* anticancer activity and provide a foundation for further investigation. However, the predicted target interactions require confirmation through direct biochemical validation assays.

## Author contributions

The manuscript was written through the contributions of all authors. Elena Daniela Hutanu: writing – original draft, visualization, methodology, investigation, data curation; Bassam A. Najri: writing – original draft, methodology, investigation, data curation, review and editing draft, software; Amine Hafis Abdelsalam: writing – original draft, data curation, visualization, methodology, investigation; Katia Mohand Saidi: formal analysis; Sevki Arslan: writing – review and editing, supervision, investigation; Arif Kivrak: project administration, supervision, review and editing draft, investigation. All authors read and approved the final manuscript.

## Conflicts of interest

The authors declare that they have no known competing financial interests or personal relationships that could have appeared to influence the work reported in this paper.

## Supplementary Material

RA-016-D6RA05827B-s001

## Data Availability

The data supporting the findings of this study are provided within the article and its supplementary information (SI). Additional raw experimental data are available from the corresponding author upon reasonable request. Supplementary information: Scheme S1, Table S1, and Fig. S1–S50. See DOI: https://doi.org/10.1039/d6ra05827b.
