# Supplementary material for: Novel benzofuran-furan-pyrano[2,3-c]pyrazole hybrids as potential anticancer agents: design, synthesis, in silico profiling, and multi-mechanistic biological evaluation
Source: RSC Adv. 2026 Aug 28;16(43):52491–517. doi: 10.1039/d6ra05827b (PMC13524203; doi:10.1039/d6ra05827b)
Supplement: Supplementary file 1 [file RA-016-D6RA05827B-s001.pdf]

Supplementary Information (SI) for RSC Advances.
This journal is © The Royal Society of Chemistry 2026

Supplementary Material

Novel Benzofuran-Furan-Pyrano[2,3-c]Pyrazole Hybrids as Potential Anticancer Agents: Design, Synthesis, *In Silico* Profiling, and Multi-Mechanistic Biological Evaluation

Elena Daniela Hutanu[a], Bassam A. Najri[b,c], Amine Hafis Abdelsalam[a], Katia Mohand Saidi[d], Sevki Arslan[a], Arif Kivrak[b,c]

[a]Department of Biology, Faculty of Science, Pamukkale University, Denizli, 20160, Turkey

[b]Department of Chemistry, Faculty of Science, Eskisehir Osmangazi University, Eskisehir 26040, Turkey

[c]Advanced Organic Synthesis Laboratory, Technology and Innovation Center, Eskisehir Osmangazi University, Eskisehir, 26040, Turkey

[d]Pharmaceutical Chemistry Team, Laboratory of Phytochemistry and Pharmacology, University of Mohamed Seddik Ben Yahia - Jijel, Ouled Aissa, BP 98, 18000 Jijel, Algeria

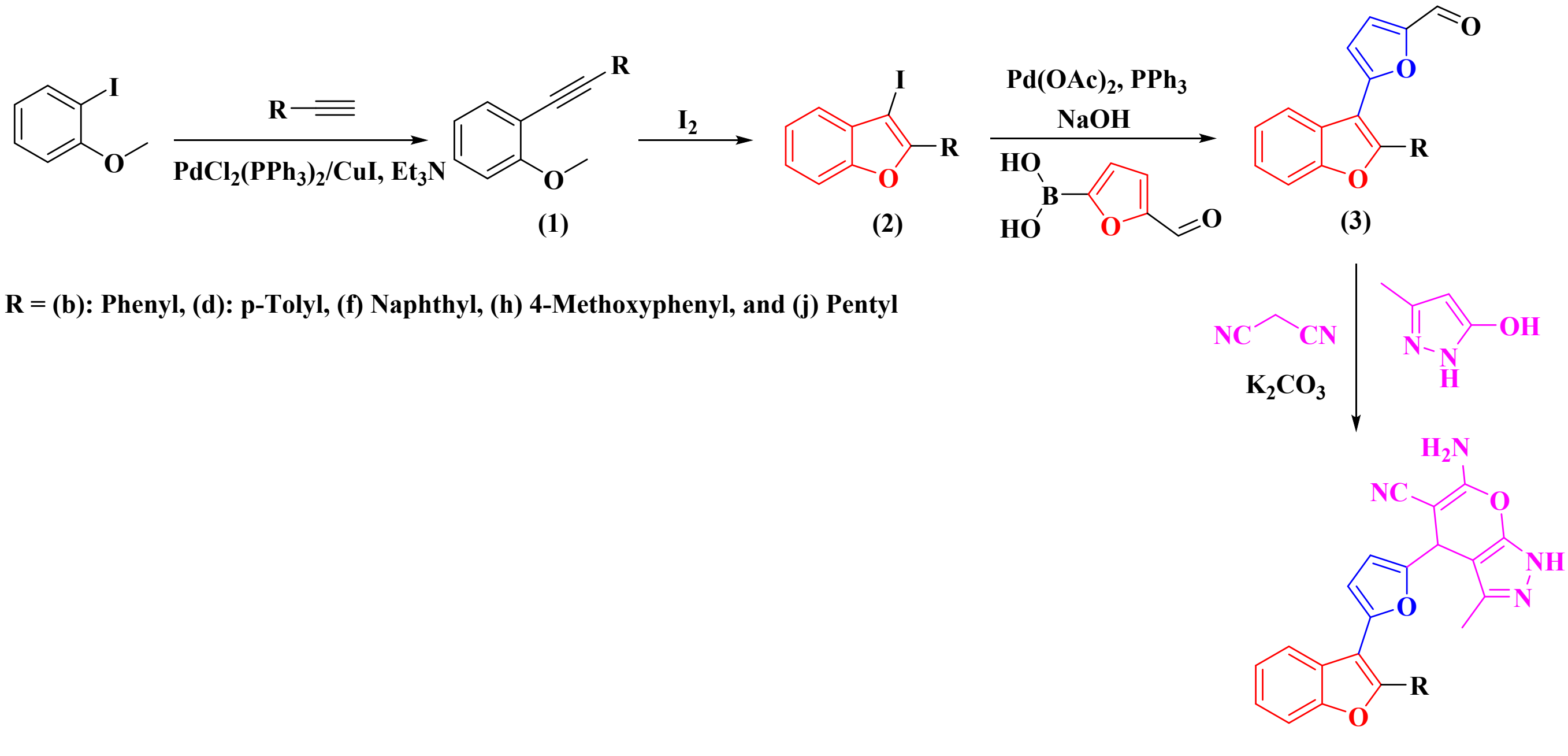

(4)

Scheme S1. Synthetic route for the preparation of benzofuran-furan-pyrano[2,3-c]pyrazole hybrids (**4b, 4d, 4f, 4h, and 4j**).

General procedure for the synthesis of 1-methoxy-2-(aryl/alkyl-ethynyl)benzene (1b, 1d, 1f, 1h, and 1j) via Sonogashira coupling.

To a solution of substituted 2-iodoanisole (1 equiv.) in tetrahydrofuran (THF) (10 mL) was added alkyne (1.2 equiv.), triethylamine (Et_3N) (15 mL), bis(triphenylphosphine)palladium(II) dichloride ($PdCl_2(PPh_3)_2$) (2.5 mol%) and copper(I) iodide (CuI) (1 mol%) which were stirred well under an argon atmosphere at room temperature overnight. The reaction mixture was diluted with ethyl acetate (EtOAc) (3×25 mL) and washed with brine solution. The organic extract was dried over anhydrous magnesium sulfate ($MgSO_4$). The crude product obtained was purified by silica gel column chromatography using hexane as the eluent to afford the 1-methoxy-2-(aryl/alkyl-ethynyl)benzene (1b, 1d, 1f, 1h, and 1j) compounds.

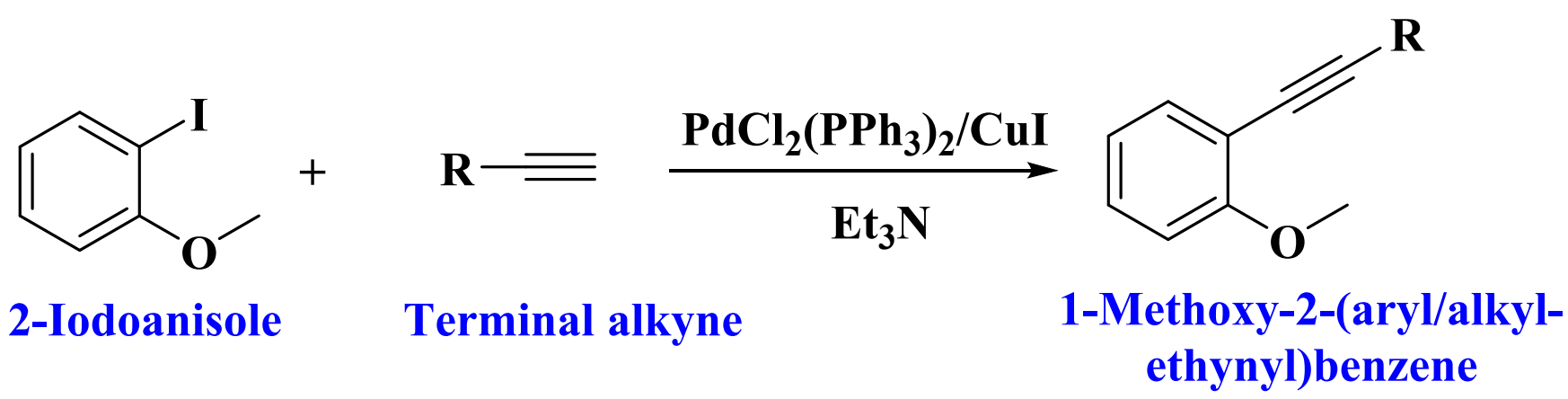

(1)

R= (b): phenyl, (d): p-tolyl, (f): naphthyl, (h): 4-methoxyphenyl, (j): pentyl

1-Methoxy-2-(phenylethynyl)benzene (1b): 2-iodoanisole (4.27 mmol, 1000 mg), phenylacetylene (5.13 mmol, 524 mg), $PdCl_2(PPh_3)_2$ (0.11 mmol, 75 mg), CuI (0.04 mmol, 8 mg), THF (10 mL) and Et_3N (15 mL) were employed to afford 889 mg of product **1b** (99% yield) as a pale yellow solid; Eluent: Hexane; ^{1}H NMR (500 MHz, $CDCl_3$) δ 7.55 (dd, J = 8.1, 1.9 Hz, 2H), 7.49 (dd, J = 7.5, 1.7 Hz, 1H), 7.36 – 7.27 (m, 4H), 6.95 – 6.88 (m, 2H), 3.91 (s, 3H); ^{13}C NMR (125 MHz, $CDCl_3$) δ 160.0, 133.7, 131.8, 129.9, 128.4, 128.2, 123.6, 120.6, 112.5, 110.8, 93.5, 85.8, 56.0.

1-Methoxy-2-(p-tolylethynyl)benzene (1d): 2-iodoanisole (4.27 mmol, 1000 mg), p-tolylacetylene (5.13 mmol, 596 mg), $PdCl_2(PPh_3)_2$ (0.11 mmol, 75 mg), CuI (0.04 mmol, 8 mg), THF (10 mL) and Et_3N (15 mL) were employed to afford 944 mg of product **1d** (99% yield) as a pale yellow solid; Eluent: Hexane; ^{1}H NMR (500 MHz, $CDCl_3$) δ 7.50 (dd, J = 7.5, 1.8 Hz, 1H), 7.46 (d, J = 8.2 Hz, 2H), 7.32 – 7.26 (m, 1H), 7.14 (d, J = 8.5 Hz, 2H), 6.93 (td, J = 7.5, 1.0 Hz, 1H), 6.89 (d, J = 8.4 Hz, 1H), 3.90 (s, 3H), 2.36 (s, 3H); ^{13}C NMR (125 MHz, $CDCl_3$) δ 160.0, 138.3, 133.6, 131.7, 129.7, 129.1, 120.6, 120.5, 112.7, 110.7, 93.7, 85.1, 55.9, 21.6.

1-((2-Methoxyphenyl)ethynyl)naphthalene (1f): 2-iodoanisole (4.27 mmol, 1000 mg), 1-ethynylnaphthalene (5.13 mmol, 780 mg), $PdCl_2(PPh_3)_2$ (0.11 mmol, 75 mg), CuI (0.04 mmol, 8 mg), THF (10 mL) and Et_3N (15 mL) were employed to afford 1097 mg of product **1f** (99% yield) as a viscous yellow oil; Eluent: Hexane; ^{1}H NMR (500 MHz, $CDCl_3$) δ 8.58 (d, J = 8.4 Hz, 1H), 7.87 – 7.81 (m, 2H), 7.78 (dd, J = 7.2, 1.1 Hz, 1H), 7.60 (td, J = 8.0, 1.6 Hz, 2H), 7.60 (td, J = 6.9, 1.2 Hz, 1H), 7.45 (t, J = 8.3, 1H), 7.38 – 7.31 (m, 1H), 6.99 (td, J = 7.5, 1.0 Hz, 1H), 6.95 (d, J = 8.4 Hz, 1H), 3.98 (s, 3H); ^{13}C NMR (125 MHz, $CDCl_3$) δ 160.2, 133.5, 133.4, 133.3, 130.2, 130.0, 128.7, 128.3, 126.8, 126.7, 126.5, 125.4, 121.4, 120.6, 112.7, 110.8, 91.9, 90.9, 56.0.

1-Methoxy-2-((4-methoxyphenyl)ethynyl)benzene (1h): 2-iodoanisole (4.27 mmol, 1000 mg), 4-ethynylanisole (5.13 mmol, 678 mg), $PdCl_2(PPh_3)_2$ (0.11 mmol, 75 mg), CuI (0.04 mmol, 8 mg), THF (10 mL) and Et_3N (15 mL) were employed to afford 1010 mg of product **1h** (99% yield) as a white solid; Eluent: Hexane; ^{1}H NMR (500 MHz, $CDCl_3$) δ 7.53 – 7.43 (m, 3H), 7.30 – 7.23 (m, 1H), 6.92 (td, J = 7.5, 1.0 Hz, 1H), 6.90 – 6.84 (m, 3H), 3.90 (s, 3H), 3.80 (s, 3H); ^{13}C NMR (125 MHz, $CDCl_3$) δ 159.8, 159.6, 133.5, 133.2, 129.6, 120.6, 115.7, 114.0, 112.8, 110.7, 93.5, 84.4, 55.9, 55.4.

1-(Hept-1-yn-1-yl)-2-methoxybenzene (1j): 2-iodoanisole (4.27 mmol, 1000 mg), 1-heptyne (5.13 mmol, 493 mg), $PdCl_2(PPh_3)_2$ (0.11 mmol, 75 mg), CuI (0.04 mmol, 8 mg), THF (10 mL) and Et_3N (15 mL) were employed to afford 1030 mg of product **1j** (99% yield) as a light orange oil; Eluent: Hexane; ^{1}H NMR (500 MHz, $CDCl_3$) δ 7.36 (dd, *J* = 7.5, 1.8 Hz, 1H), 7.25 – 7.20 (m, 1H), 6.86 (td, *J* = 7.4, 1.0 Hz, 1H), 6.83 (d, *J* = 8.4 Hz, 1H), 3.86 (s, 3H), 2.45 (t, *J* = 7.2 Hz, 2H), 1.66 – 1.58 (m, 2H), 1.48 – 1.40 (m, 2H), 1.38 – 1.30 (m, 2H), 0.91 (t, *J* = 7.2 Hz, 3H); ^{13}C NMR (125 MHz, $CDCl_3$) δ 159.9, 133.7, 129.0, 120.5, 113.2, 110.5, 94.9, 76.7, 55.9, 31.2, 28.7, 22.4, 19.9, 14.2.

General procedure for the synthesis of 3-iodo-2-aryl-benzofuran (2b, 2d, 2f, and 2h) via iodocyclization.

To a solution of (1b, 1d, 1f, and 1h**)** (1 equiv.) in dichloromethane (DCM) (25 mL) was added iodine (I_2) (1 equiv.), and stirring was continued at room temperature for 4 h under an argon atmosphere. The reaction mixture was quenched with saturated sodium thiosulfate pentahydrate ($Na_2S_2O_3.5H_2O$) solution, and extracted with chloroform ($CHCl_3$) (3×25 mL). The combined organic extracts were dried over anhydrous $MgSO_4$. The crude product was chromatographed on silica gel using hexane/EtOAc as the eluent to afford the 3-iodo-2-aryl-benzofuran (2b, 2d, 2f, and 2h).

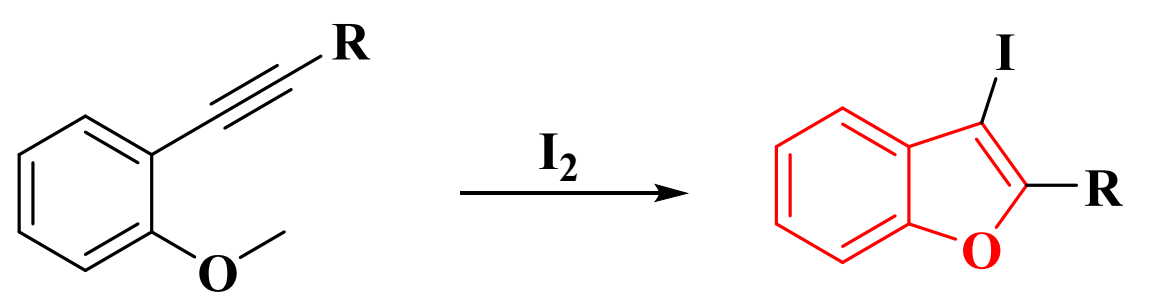

1-Methoxy-2-(aryl/alkyl-ethynyl)benzene (1)

3-Iodo-2-aryl/alkyl-benzofuran (2)

R= (b): phenyl, (d): p-tolyl, (f): naphthyl, (h): 4-methoxyphenyl, (j): pentyl

3-Iodo-2-phenylbenzofuran (2b): 1-methoxy-2-(phenylethynyl)benzene (1b) (3.84 mmol, 800 mg), I_2 (7.68 mmol, 1950 mg) and DCM (25 mL) were employed to afford 1210 mg of product **2b** (98% yield) as a pale yellow solid; Eluent: Hexane; ^{1}H NMR (500 MHz, $CDCl_3$) δ 8.21 – 8.15 (m, 2H), 7.52 – 7.40 (m, 5H), 7.39 – 7.29 (m, 2H); ^{13}C NMR (100 MHz, $CDCl_3$) δ 154.0, 153.1, 132.6, 130.1, 129.4, 128.6, 127.6, 125.8, 123.6, 122.0, 111.3, 61.3.

3-Iodo-2-(p-tolyl)benzofuran (2d): 1-methoxy-2-(p-tolylethynyl)benzene (1d) (3.88 mmol, 800 mg), I_2 (7.76 mmol, 1969 mg) and DCM (25 mL) were employed to afford 1260 mg of product **2d** (97% yield) as a yellow solid; Eluent: Hexane; ^{1}H NMR (400 MHz, $CDCl_3$) δ 8.08 – 8.05 (m, 2H), 7.48 – 7.42 (m, 2H), 7.37 – 7.28 (m, 4H), 2.42 (s, 3H); ^{13}C NMR (125 MHz,

$CDCl_3$) δ 153.9, 153.4, 139.5, 132.6, 129.3, 127.4, 127.3, 125.6, 123.5, 121.8, 111.2, 60.5, 21.6.

3-Iodo-2-(naphthalen-1-yl)benzofuran (2f): 1-((2-methoxyphenyl)ethynyl)naphthalene (1f) (3.10 mmol, 800 mg), I_2 (6.19 mmol, 1572 mg) and DCM (25 mL) were employed to afford 1090 mg of product **2f** (95% yield) as a white solid; Eluent: Hexane; ^{1}H NMR (500 MHz, $CDCl_3$) δ 8.00 (d, *J* = 8.3 Hz, 1H), 7.97 – 7.90 (m, 2H), 7.82 (dd, *J* = 7.1, 1.1 Hz, 1H), 7.62 – 7.49 (m, 5H), 7.45 – 7.36 (m, 2H); ^{13}C NMR (125 MHz, $CDCl_3$) δ 155.5, 154.8, 133.8, 131.7 (2C),130.7, 130.1, 128.6, 127.4, 127.0, 126.5, 126.2, 125.8, 125.1, 123.7, 121.9, 111.6, 66.3.

3-Iodo-2-(4-methoxyphenyl)benzofuran (2h): 1-methoxy-2-((4-methoxyphenyl)ethynyl) benzene (1h) (3.36 mmol, 800 mg), I_2 (6.71 mmol, 1704 mg) and DCM (25 mL) were employed to afford 1153 mg of product **2h** (98% yield) as a pale yellow solid; Eluent: Hexane; ^{1}H NMR (500 MHz, $CDCl_3$) δ 8.14 – 8.09 (m, 2H), 7.47 – 7.40 (m, 2H), 7.35 – 7.27 (m, 2H), 7.04 – 6.99 (m, 2H), 3.87 (s, 3H); ^{13}C NMR (125 MHz, $CDCl_3$) δ 160.4, 153.8, 153.3, 132.7, 129.1, 125.3, 123.5, 122.7, 121.6, 114.0, 111.1, 59.6, 55.5.

General procedure for the synthesis of 3-iodo-2-alkyl-benzofuran (2j) via iodocyclization.

To a solution of **(1j)** (1 equiv.) in 1,2-dichloroethane (25 mL) was added sodium bicarbonate (3 equiv.) and iodine (3 equiv.). The reaction mixture was refluxed at 70 °C overnight under an argon atmosphere. The reaction mixture was quenched with saturated sodium thiosulfate solution, and extracted with chloroform (3×25 mL). The combined organic extracts were dried over anhydrous $MgSO_4$. The crude product was chromatographed on silica gel using hexane/ethyl acetate as the eluent to afford the 3-iodo-2-alkylbenzofuran (2j).

3-Iodo-2-pentylbenzofuran (2j): 1-(hept-1-yn-1-yl)-2-methoxybenzene (1j) (3.95 mmol, 800 mg), sodium bicarbonate (11.86 mmol, 997 mg), iodine (11.86 mmol, 3011 mg) and 1,2-dichloroethane (25 mL) were employed to afford 1213 mg of product **2j** (98% yield) as a white oil; Eluent: Hexane; ^{1}H NMR (500 MHz, $CDCl_3$) δ 7.40 – 7.36 (m, 1H), 7.33 – 7.30 (m, 1H), 7.28 – 7.24 (m, 2H), 2.85 (t, *J* = 7.49 Hz, 2H), 1.77 – 1.70 (m, 2H), 1.37 – 1.33 (m, 4H), 0.90 (t, *J* = 7.15 Hz, 3H); ^{13}C NMR (125 MHz, $CDCl_3$) δ 159.3, 154.3, 131.2, 124.6, 123.2, 120.9, 111.0, 62.6, 31.3, 28.0, 27.6, 22.5, 14.1.

General procedure for the synthesis of 5-(2-aryl/alkyl-benzofuran-3-yl)furan-2-carbaldehyde (3b, 3d, 3f, 3h, and 3j) via Suzuki-Miyaura coupling reaction.

Into a microwave vial, to a solution of (2b, 2d, 2f, 2h, and 2j), in THF (5 mL), palladium(II) acetate ($Pd(OAc)_2$) (10 mol%), triphenylphosphine (PPh_3) (20 mol%), 5-formylfuran-2-boronic acid (1.5 equiv.) and sodium hydroxide (NaOH) (3 equiv.) were added at room temperature under an argon atmosphere, followed by an addition of H_2O (1 mL). The vial was submerged in the microwave cavity for 45 min at 70 °C. The post-reaction mixture was cooled to room temperature. The reaction mixture was diluted with EtOAc (3×25 mL) and washed with brine solution. The organic phases were combined and dried with $MgSO_4$. The drying agent was filtered off and the crude product purified by silica gel column chromatography using hexane/EtOAc as the eluent to afford 5-(2-aryl/alkylbenzofuran-3-yl)furan-2-carbaldehydes (3b, 3d, 3f, 3h, and 3j).

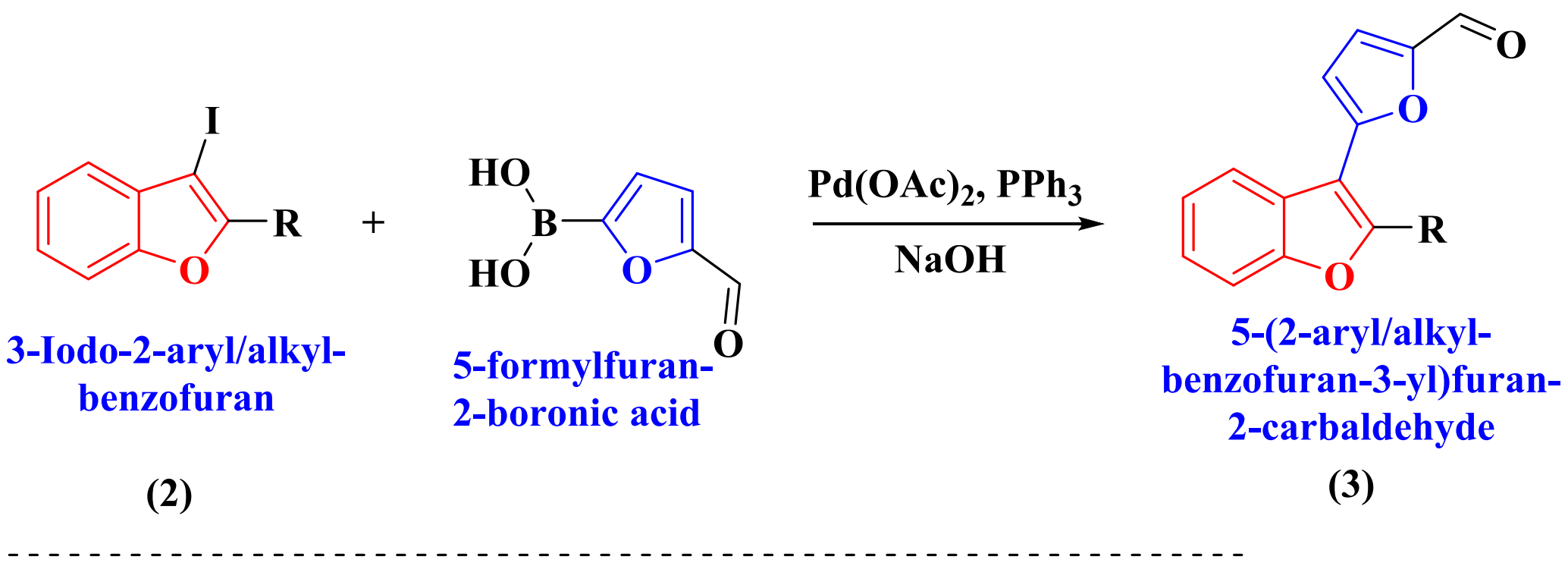

R= (b): phenyl, (d): p-tolyl, (f): naphthyl, (h): 4-methoxyphenyl, (j): pentyl

5-(2-Phenylbenzofuran-3-yl)furan-2-carbaldehyde (3b): 3-iodo-2-phenylbenzofuran (2b) (0.62 mmol, 200 mg), $Pd(OAc)_2$ (0.06 mmol, 14 mg), PPh_3 (0.12 mmol, 33 mg), 5-formylfuran-2-boronic acid (0.94 mmol, 131 mg), NaOH (1.87 mmol, 75 mg), THF (5 mL) and water (1 mL) were employed to afford 144 mg of product **3b** (80% yield) as a red solid; Eluent: hexane/EtOAc (3/1); ^{1}H NMR (500 MHz, $CDCl_3$) δ 9.66 (s, 1H), 8.05 – 7.97 (m, 1H), 7.82 – 7.80 (m, 2H), 7.54 (dd, J = 7.0, 1.2 Hz, 1H), 7.49 – 7.43 (m, 3H), 7.40 – 7.33 (m, 2H), 7.32 (d, J = 3.7 Hz, 1H), 6.72 (d, J = 3.7 Hz, 1H); ^{13}C NMR (125 MHz, $CDCl_3$) δ 177.2, 154.4, 154.2, 154.0, 152.0, 130.1, 130.0, 128.9, 128.3, 127.1, 125.6, 125.4, 124.0, 121.6, 111.4, 110.5, 107.3.

5-(2-(p-Tolyl)benzofuran-3-yl)furan-2-carbaldehyde (3d): 3-iodo-2-(p-tolyl)benzofuran (2d) (0.60 mmol, 200 mg), $Pd(OAc)_2$ (0.06 mmol, 13 mg), PPh_3 (0.12 mmol, 31 mg), 5-formylfuran-2-boronic acid (0.90 mmol, 126 mg), NaOH (1.80 mmol, 72 mg), THF (5 mL) and water (1 mL) were employed to afford 147 mg of product **3d** (81% yield) as a red solid;

Eluent: hexane/EtOAc (3/1); ^{1}H NMR (500 MHz, $CDCl_3$) 9.66 (s, 1H), 8.04 – 7.96 (m, 1H), 7.70 (d, J = 8.3 Hz, 2H), 7.54 – 7.51 (m, 1H), 7.38 – 7.32 (m, 2H), 7.32 – 7.26 (m, 3H), 6.72 (d, J = 3.7 Hz, 1H), 2.42 (s, 3H); ^{13}C NMR (125 MHz, $CDCl_3$) δ 177.1, 154.8, 154.3, 154.1, 151.9, 140.8, 129.6, 128.2, 127.2, 127.1, 125.4 (2C), 123.9, 121.5, 111.4, 110.3, 106.8, 21.7.

5-(2-(Naphthalen-1-yl)benzofuran-3-yl)furan-2-carbaldehyde (3f): 3-iodo-2-(naphthalen-1-yl)-benzofuran (2f) (0.54 mmol, 200 mg), $Pd(OAc)_2$ (0.05 mmol, 12 mg), PPh_3 (0.11 mmol, 28 mg), 5-formylfuran-2-boronic acid (0.81 mmol, 113 mg), NaOH (1.62 mmol, 65 mg), THF (5 mL) and water (1 mL) were employed to afford 152 mg of product **3f** (83% yield) as a red solid; Eluent: hexane/EtOAc (3/1); ^{1}H NMR (500 MHz, $CDCl_3$) δ 9.57 (s, 1H), 8.35 – 8.31 (m, 1H), 8.05 (d, J = 8.3 Hz, 1H), 7.96 (d, J = 8.2 Hz, 1H), 7.78 – 7.74 (m, 2H), 7.62 – 7.58 (m, 2H), 7.56 – 7.52 (m, 1H), 7.47 – 7.43 (m, 3H), 7.07 (d, J = 3.8 Hz, 1H), 5.95 (d, J = 3.8 Hz, 1H); ^{13}C NMR (125 MHz, $CDCl_3$) δ 177.0, 154.8, 154.4, 153.8, 151.6, 133.8, 131.5, 131.1, 129.4, 128.7, 127.6, 127.4, 126.7, 125.9, 125.7, 125.5, 125.4, 124.2, 122.4, 112.0, 111.6, 110.4, 109.7.

5-(2-(4-Methoxyphenyl)benzofuran-3-yl)furan-2-carbaldehyde (3h): 3-iodo-2-(4-methoxyphenyl)benzofuran (2h) (0.57 mmol, 200 mg), $Pd(OAc)_2$ (0.06 mmol, 13 mg), PPh_3 (0.11 mmol, 30 mg), 5-formylfuran-2-boronic acid (0.86 mmol, 120 mg), NaOH (1.71 mmol, 69 mg), THF (5 mL) and water (1 mL) were employed to afford 165 mg of product **3h** (91% yield) as a red solid; Eluent: hexane/EtOAc (3/1); ^{1}H NMR (500 MHz, $CDCl_3$) δ 9.66 (s, 1H), 7.99 – 7.97 (m, 1H), 7.78 – 7.74 (m, 2H), 7.53 – 7.50 (m, 1H), 7.38 – 7.31 (m, 3H), 7.00 – 6.97 (m, 2H), 6.72 (d, J = 3.7 Hz, 1H), 3.87 (s, 3H); ^{13}C NMR (125 MHz, $CDCl_3$) δ 177.1, 161.0, 154.7, 154.4, 154.0, 151.8, 129.8, 127.3, 125.2, 123.9, 122.4, 121.3, 114.3, 111.3 (2C), 110.2, 106.2, 55.5.

5-(2-Pentylbenzofuran-3-yl)furan-2-carbaldehyde (3j): 3-iodo-2-pentylbenzofuran (2j) (0.68 mmol, 200 mg), $Pd(OAc)_2$ (0.07 mmol, 15 mg), PPh_3 (0.14 mmol, 36 mg), 5-formylfuran-2-boronic acid (1.03 mmol, 144 mg), NaOH (2.05 mmol, 82 mg), THF (5 mL) and water (1 mL) were employed to afford 174 mg of product **3j** (90% yield) as a light orange oil; Eluent: hexane/EtOAc (3/1); ^{1}H NMR (500 MHz, $CDCl_3$) δ 9.66 (s, 1H), 7.90 – 7.87 (m, 1H), 7.48 – 7.44 (m, 1H), 7.37 (d, J = 3.7 Hz, 1H), 7.33 – 7.29 (m, 2H), 6.74 (d, J = 3.7 Hz, 1H), 3.09 (t, J = 7.58 Hz, 2H), 1.85 – 1.79 (m, 2H), 1.42 – 1.33 (m, 4H), 0.89 (t, J = 7.2 Hz, 3H); ^{13}C NMR (125 MHz, $CDCl_3$) δ 176.9, 159.5, 154.6, 154.0, 151.5, 126.0, 124.6, 123.6, 120.5, 111.2 (2C), 108.8, 107.1, 31.6, 28.3, 27.7, 22.5, 14.1.

General procedure for the synthesis of benzofuran-furan-pyrano[2,3-c]pyrazole hybrids (4b, 4d, 4f, 4h, and 4j).

To a stirred ethanolic solution of the appropriate benzofuran-furan aldehyde derivative (3b, 3d, 3f, 3h, or 3j) (1.0 equiv.) were added malononitrile (2.0 equiv.) and 3-methyl-1H-pyrazol-5-ol (1.5 equiv.). Potassium carbonate (K_2CO_3, 10 mol%) was subsequently added, and the reaction mixture was stirred at room temperature. Rapid formation of a solid product was observed. The reaction progress was monitored by TLC, and after 2 min the spot corresponding to the starting aldehyde was no longer detectable. Stirring was then stopped, and the precipitated product was immediately isolated by filtration and washed with cold ethanol (EtOH). No separate chemical quenching step was required because the product was directly separated from the reaction medium by filtration. The isolated solid was dried and recrystallized from EtOH to furnish the desired benzofuran-furan-pyrano[2,3-c]pyrazole hybrids (**4b**, **4d**, **4f**, **4h**, and **4j**).

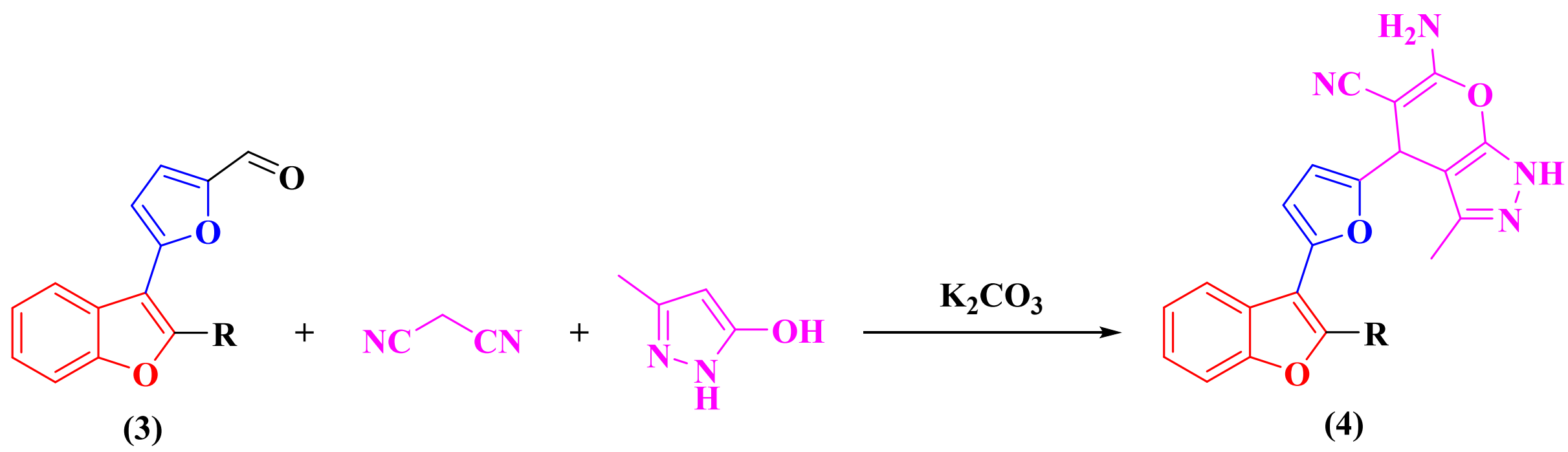

R = (b): Phenyl, (d): p-Tolyl, (f) Naphthyl, (h) 4-Methoxyphenyl, and (j) Pentyl

6-Amino-3-methyl-4-(5-(2-phenylbenzofuran-3-yl)furan-2-yl)-1,4-dihydropyrano[2,3-c]pyrazole-5-carbonitrile (4b): 5-(2-phenylbenzofuran-3-yl)furan-2-carbaldehyde (3b) (0.35 mmol, 100 mg), K_2CO_3 (0.04 mmol, 5.53 mg), malononitrile (0.70 mmol, 46.24 mg), 3-methyl-1H-pyrazol-5-ol (0.53 mmol, 51.51 mg), and EtOH (10 mL) were employed to afford 145 mg of product **4b** (96% yield) as an orange solid; ^{1}H NMR (500 MHz, $CDCl_3$) δ 12.15 (s, 1H), 7.71 (t, J = 7.0 Hz, 3H), 7.62 (d, J = 8.2 Hz, 1H), 7.43 – 7.34 (m, 4H), 7.29 (t, J = 7.5 Hz, 1H), 6.98 (s, 2H), 6.73 (d, J = 3.3 Hz, 1H), 6.45 (d, J = 3.3 Hz, 1H), 4.83 (s, 1H), 1.91 (s, 3H); ^{13}C NMR (125 MHz, $CDCl_3$) δ 162.1, 156.0, 155.4, 153.8, 151.2, 145.1, 136.3, 130.1, 129.8, 129.2, 127.8, 127.6, 126.0, 124.2, 121.2, 111.8, 110.4, 108.6, 108.1, 95.5, 54.5, 30.6, 10.0; HRMS (ESI$^+$) m/z: calcd for $C_{26}H_{19}N_4O_3$ $[M+H]^+$ 435.14517; found 435.14582.

6-Amino-3-methyl-4-(5-(2-(p-tolyl)benzofuran-3-yl)furan-2-yl)-1,4-dihydropyrano[2,3-c]pyrazole-5-carbonitrile (4d): 5-(2-(p-tolyl)benzofuran-3-yl)furan-2-carbaldehyde (3d) (0.33 mmol, 100 mg), K_2CO_3 (0.03 mmol, 4.56 mg), malononitrile (0.66 mmol, 43.60 mg), 3-methyl-1H-pyrazol-5-ol (0.50 mmol, 48.56 mg), and EtOH (10 mL) were employed to afford 139 mg of product **4d** (94% yield) as an orange solid; ^{1}H NMR (500 MHz, $CDCl_3$) δ 12.15 (s, 1H), 7.69 (d, *J* = 7.8 Hz, 1H), 7.59 (dd, *J* = 11.8, 8.1 Hz, 3H), 7.34 (t, *J* = 7.7 Hz, 1H), 7.27 (t, *J* = 7.6 Hz, 1H), 7.20 (d, *J* = 7.8 Hz, 2H), 6.99 (s, 2H), 6.70 (d, *J* = 3.3 Hz, 1H), 6.45 (d, *J* = 3.3 Hz, 1H), 4.83 (s, 1H), 2.31 (s, 3H), 1.92 (s, 3H); ^{13}C NMR (125 MHz, $CDCl_3$) δ 162.0, 155.9, 155.4, 153.7, 151.5, 145.3, 139.6, 136.3, 129.8, 127.9, 127.5, 127.2, 125.8, 124.1, 121.2, 121.0, 111.7, 111.7, 108.6, 107.4, 95.5, 54.5, 30.6, 21.5, 10.0; HRMS (ESI$^+$) m/z: calcd for $C_{27}H_{21}N_4O_3$ $[M+H]^+$ 449.16082; found 449.16207.

6-Amino-3-methyl-4-(5-(2-(naphthalen-1-yl)benzofuran-3-yl)furan-2-yl)-1,4-dihydropyrano[2,3-c]pyrazole-5-carbonitrile (4f): 5-(2-(naphthalen-1-yl)benzofuran-3-yl)furan-2-carbaldehyde (3f) (0.30 mmol, 100 mg), K_2CO_3 (0.03 mmol, 4.15 mg), malononitrile (0.60 mmol, 39.64 mg), 3-methyl-1H-pyrazol-5-ol (0.45 mmol, 44.15 mg), and EtOH (10 mL) were employed to afford 130 mg of product **4f** (91% yield) as an orange solid; ^{1}H NMR (500 MHz, $CDCl_3$) δ 12.07 (s, 1H), 8.09 (d, *J* = 8.3 Hz, 1H), 8.03 – 8.00 (m, 2H), 7.78 – 7.75 (m, 1H), 7.66 (d, *J* = 8.3 Hz, 1H), 7.61 (t, *J* = 7.7 Hz, 2H), 7.55 – 7.52 (m, 1H), 7.45 – 7.41 (m, 2H), 7.35 (dd, *J* = 9.3, 6.0 Hz, 1H), 6.97 (s, 2H), 6.07 (d, *J* = 3.3 Hz, 1H), 6.00 (d, *J* = 3.4 Hz, 1H), 4.67 (s, 1H), 1.74 (s, 3H); ^{13}C NMR (125 MHz, $CDCl_3$) δ 162.2, 156.1, 155.3, 154.5, 150.2, 146.2, 142.8, 136.3, 133.7, 131.5, 131.0, 130.0, 129.1, 127.8, 127.6, 126.9, 126.4, 125.9, 125.4, 124.2, 122.0, 121.3, 112.0, 111.0, 108.6, 107.6, 95.4, 54.1, 30.7, 9.9; HRMS (ESI$^+$) m/z: calcd for $C_{30}H_{21}N_4O_3$ $[M+H]^+$ 485.16082; found 485.16019.

6-Amino-4-(5-(2-(4-methoxyphenyl)benzofuran-3-yl)furan-2-yl)-3-methyl-1,4-dihydropyrano[2,3-c]pyrazole-5-carbonitrile (4h): 5-(2-(4-methoxyphenyl)benzofuran-3-yl)furan-2-carbaldehyde (3h) (0.31 mmol, 100 mg), K_2CO_3 (0.03 mmol, 4.28 mg), malononitrile (0.62 mmol, 40.96 mg), 3-methyl-1H-pyrazol-5-ol (0.47 mmol, 46.11 mg), and EtOH (10 mL) were employed to afford 136 mg of product **4h** (93% yield) as an orange solid; ^{1}H NMR (500 MHz, $CDCl_3$) δ 12.17 (s, 1H), 7.66 (dd, *J* = 18.5, 8.2 Hz, 3H), 7.59 (d, *J* = 8.2 Hz, 1H), 7.33 (t, *J* = 7.7 Hz, 1H), 7.26 (t, *J* = 7.5 Hz, 1H), 7.00 (s, 2H), 6.93 (dd, *J* = 7.1, 4.9 Hz, 2H), 6.70 (d, *J* = 3.3 Hz, 1H), 6.46 (d, *J* = 3.3 Hz, 1H), 4.84 (s, 1H), 3.79 (s, 3H), 1.92 (s, 3H); ^{13}C NMR (125 MHz, $CDCl_3$) δ 162.0, 160.5, 155.6, 153.6, 151.4, 145.4, 136.4, 132.0, 129.1, 128.0, 126.0, 124.1, 122.4, 120.9, 118.0, 114.6, 111.6, 110.0, 108.7, 106.6, 95.5, 55.8,

54.5, 30.6, 10.0; HRMS (ESI^+) m/z: calcd for $C_{27}H_{21}N_4O_4$ $[M+H]^+$ 465.15574; found 465.15567.

6-Amino-3-methyl-4-(5-(2-pentylbenzofuran-3-yl)furan-2-yl)-1,4-dihydropyrano[2,3-c]pyrazole-5-carbonitrile (4j): 5-(2-pentylbenzofuran-3-yl)furan-2-carbaldehyde (3j) (0.35 mmol, 100 mg), K_2CO_3 (0.04 mmol, 5.53 mg), malononitrile (0.70 mmol, 46.24 mg), 3-methyl-1H-pyrazol-5-ol (0.53 mmol, 52 mg), and EtOH (10 mL) were employed to afford 145 mg of product **4j** (95% yield) as an orange solid; ^1H NMR (500 MHz, $CDCl_3$) δ 12.16 (s, 1H), 7.79 – 7.72 (m, 1H), 7.54 – 7.47 (m, 1H), 7.28 – 7.20 (m, 2H), 6.98 (s, 2H), 6.68 (d, J = 3.3 Hz, 1H), 6.32 (d, J = 3.3 Hz, 1H), 4.83 (s, 1H), 2.95 – 2.84 (m, 2H), 1.97 (s, 3H), 1.59 (p, J = 7.2 Hz, 2H), 1.25 – 1.16 (m, 4H), 0.78 (t, J = 6.5 Hz, 3H); ^{13}C NMR (125 MHz, $CDCl_3$) δ 162.2, 155.7, 155.5, 155.2, 153.8, 146.5, 136.2, 126.2, 124.7, 123.7, 121.3, 120.7, 111.5, 107.9 (2C), 107.8, 95.4, 63.3, 54.4, 31.2, 27.9, 27.7, 22.4, 14.4, 10.1; HRMS (ESI^+) m/z: calcd for $C_{25}H_{25}N_4O_3$ $[M+H]^+$ 429.19212; found 429.19265.

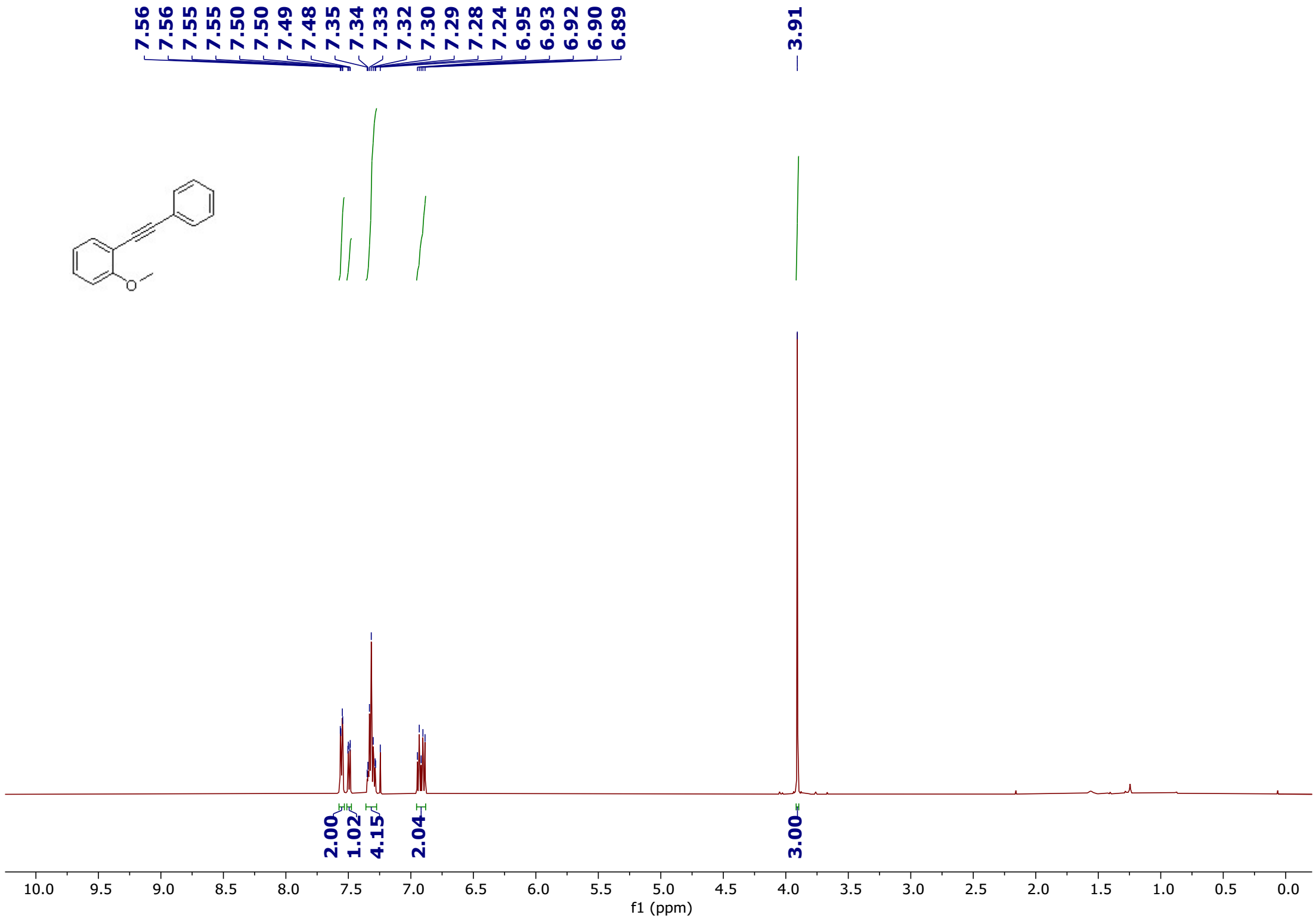

Figure S1. ^{1}H NMR spectrum of 1b.

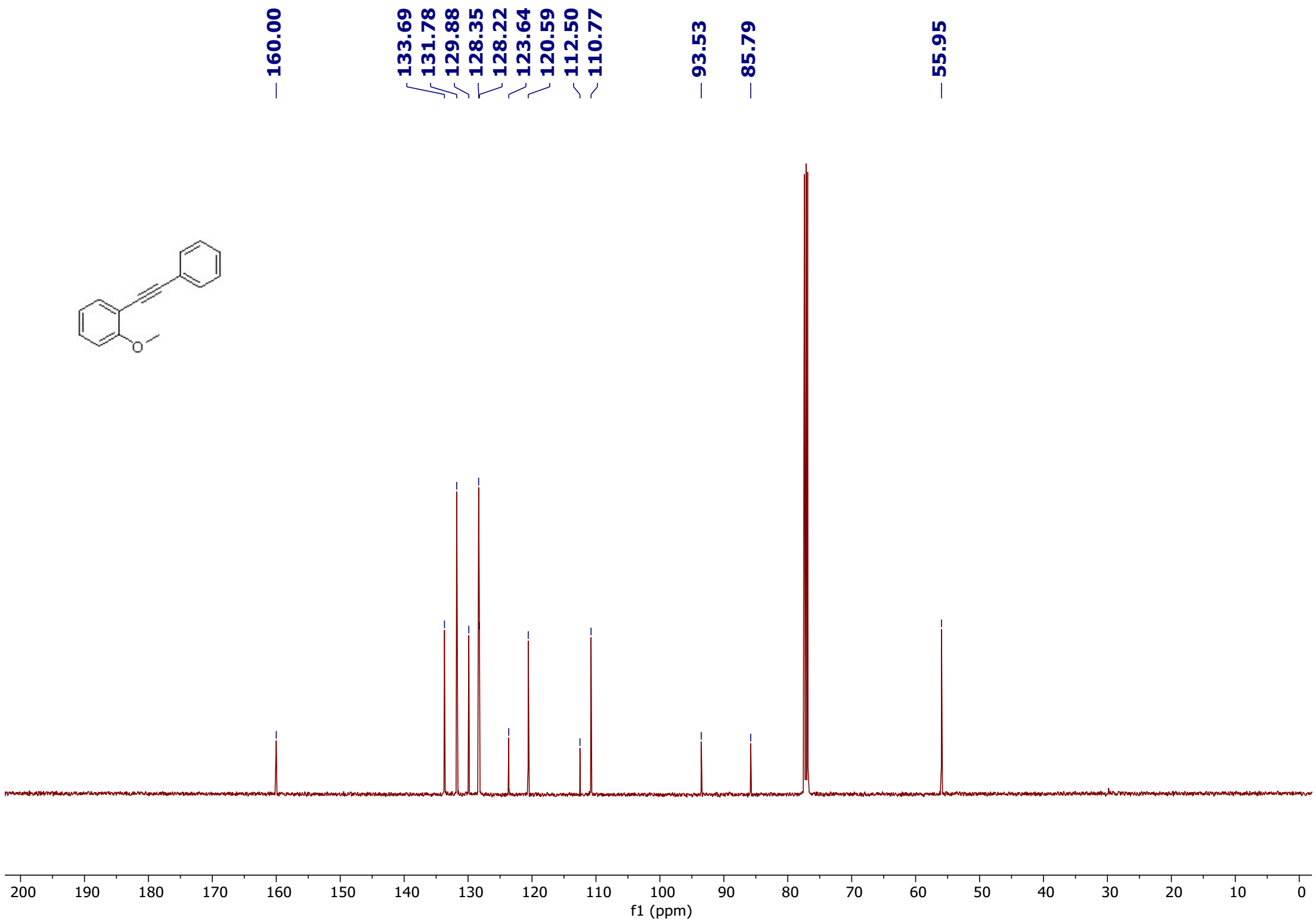

Figure S2. ^{13}C NMR spectrum of 1b.

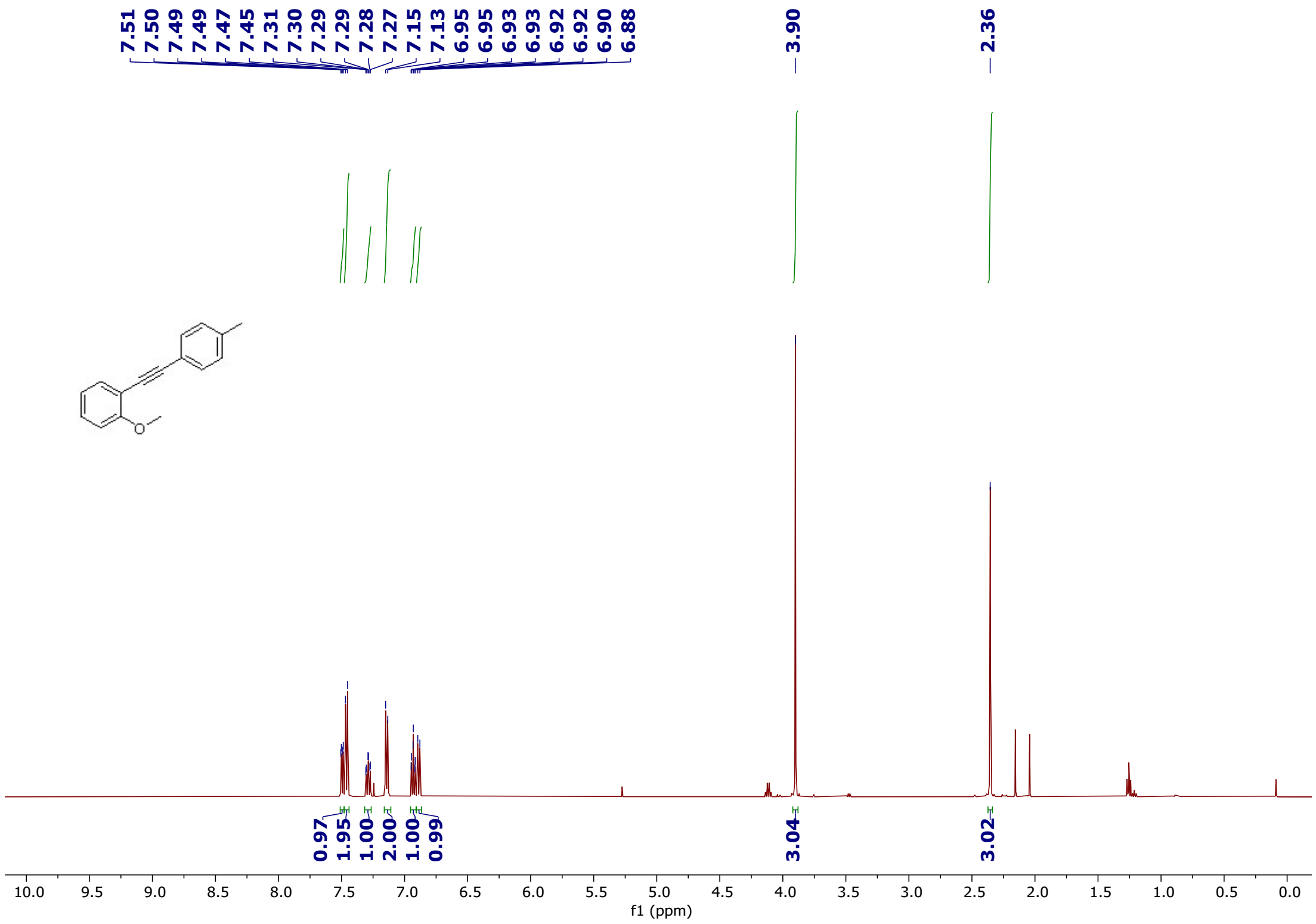

Figure S3. ^{1}H NMR spectrum of 1d.

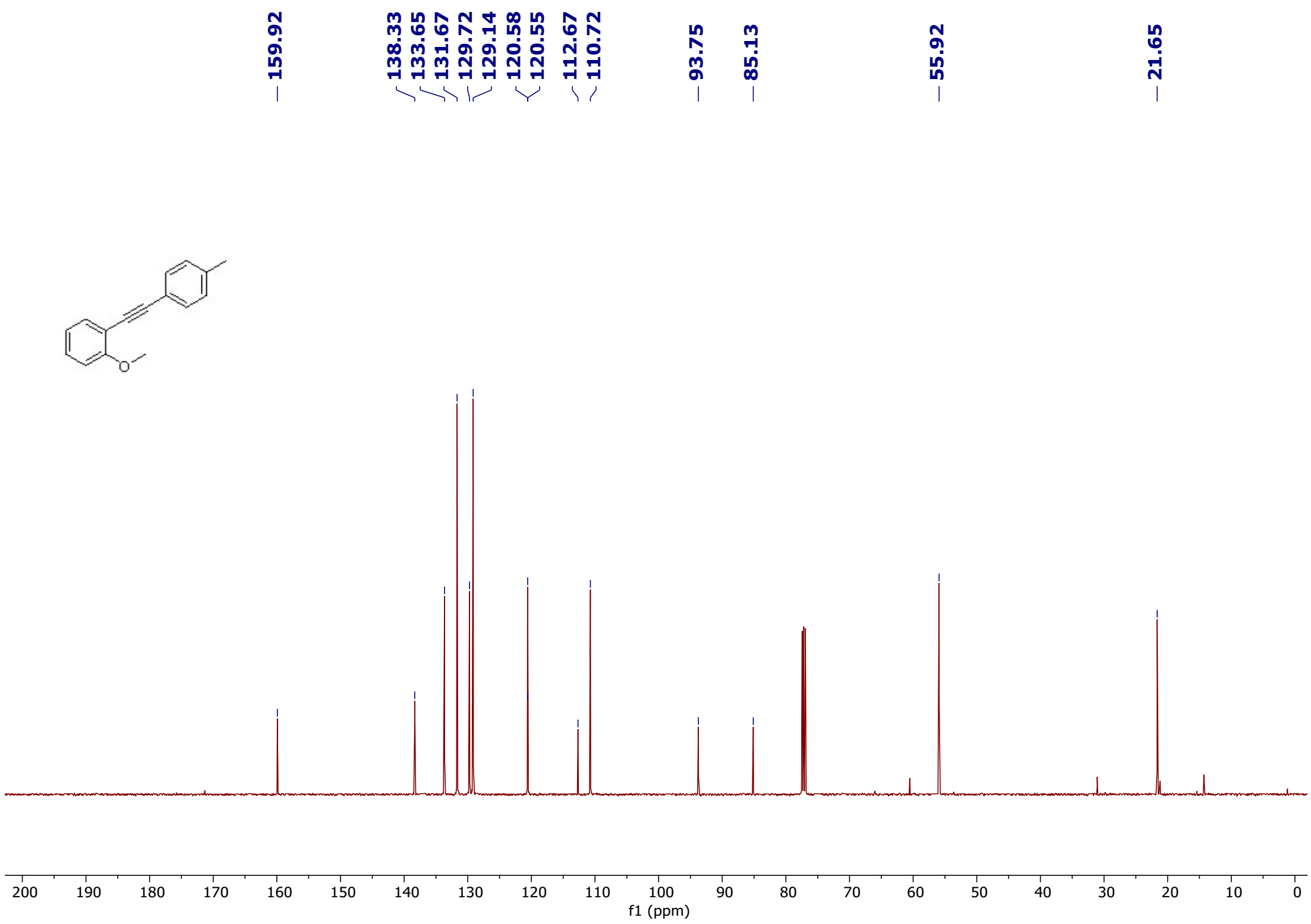

Figure S4. ^{13}C NMR spectrum of 1d.

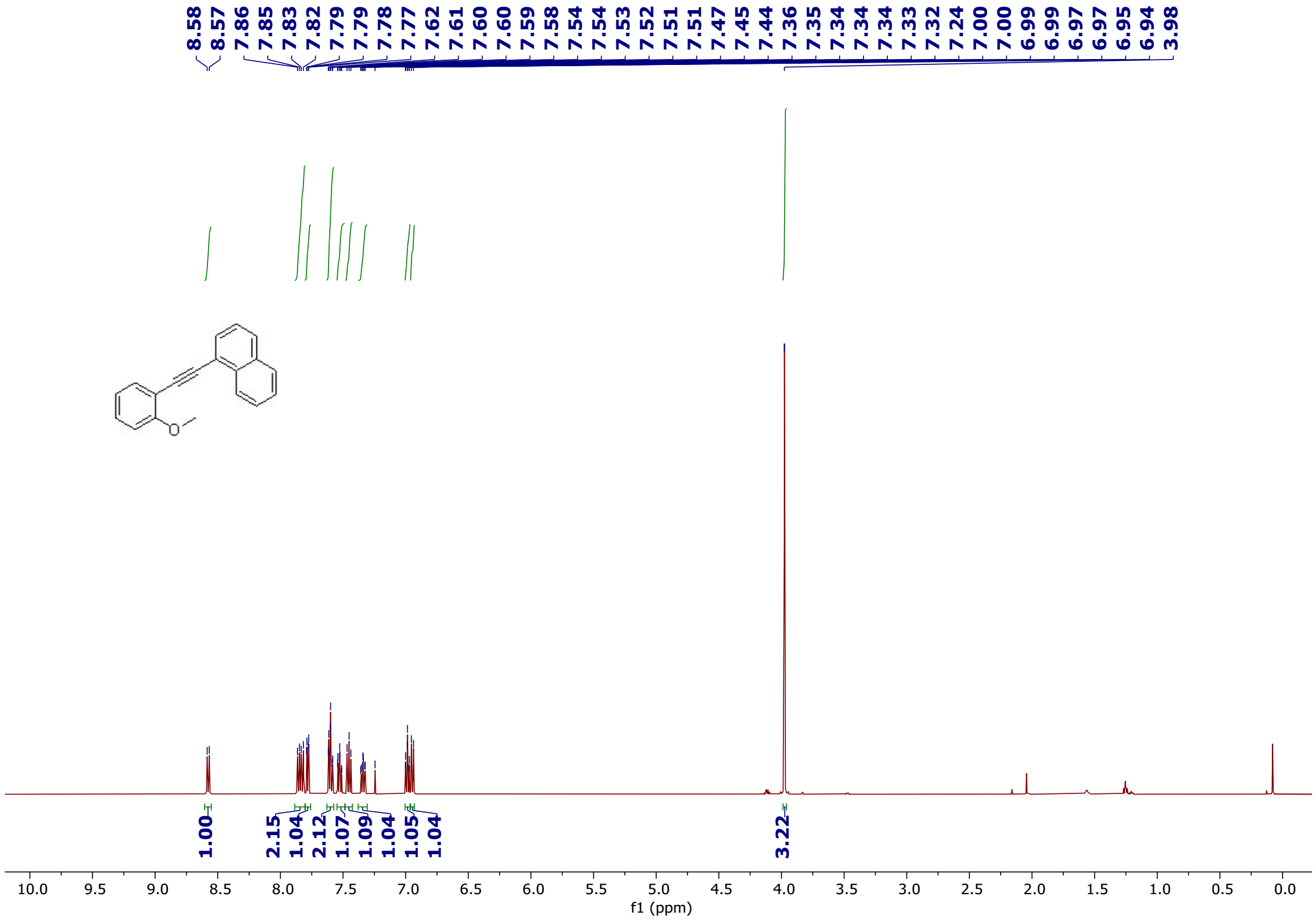

Figure S5. ^{1}H NMR spectrum of 1f.

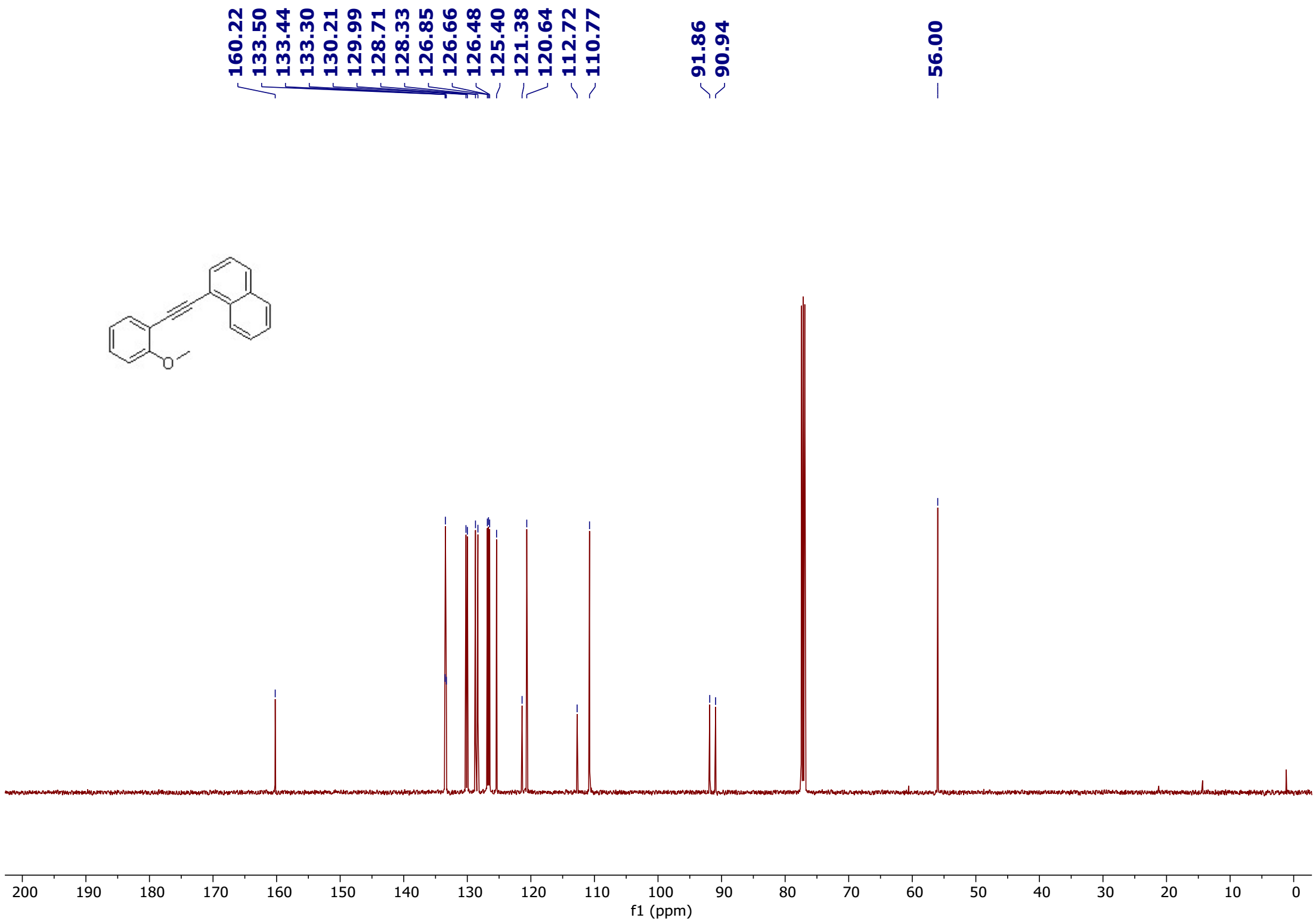

Figure S6. ^{13}C NMR spectrum of 1f.

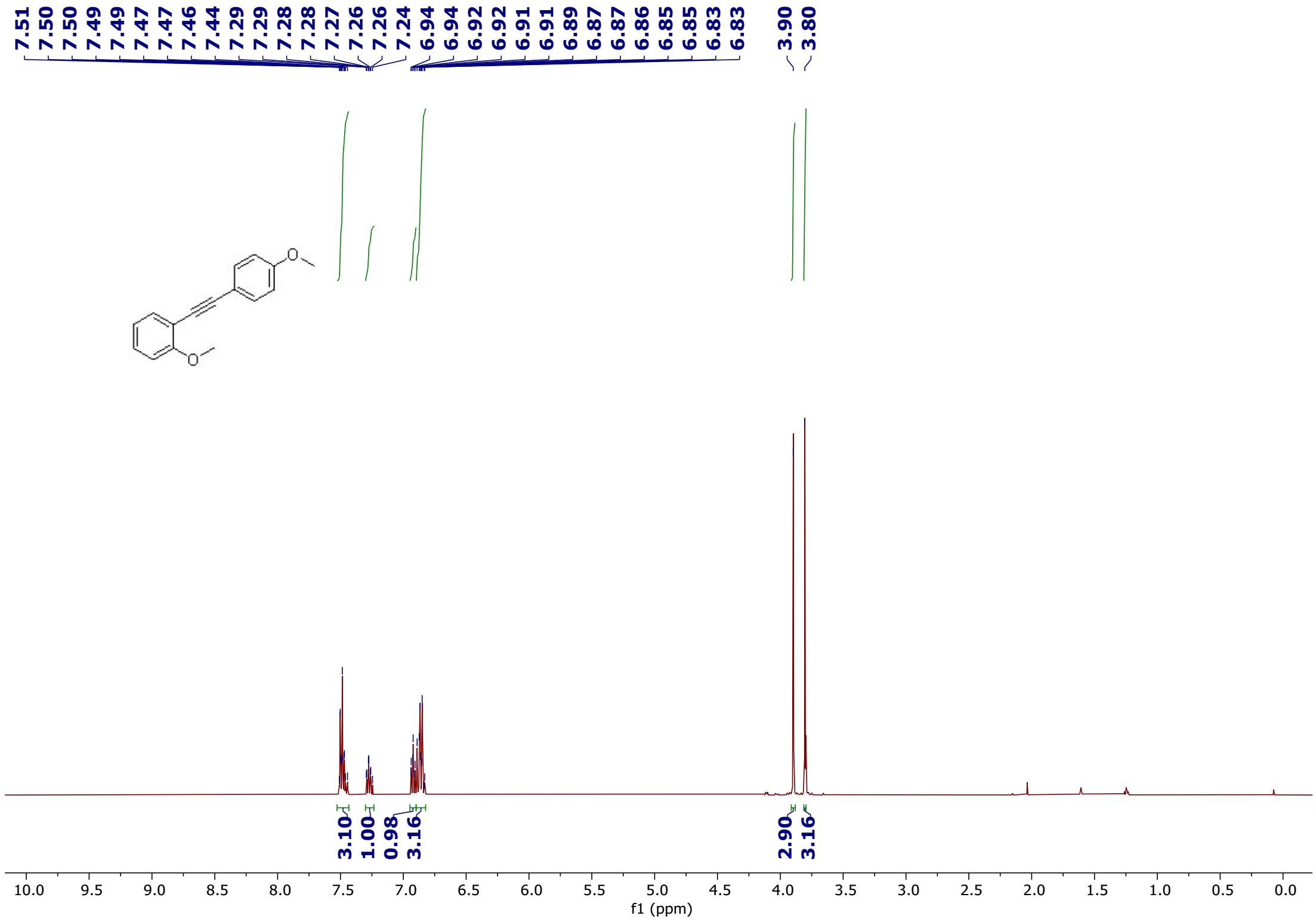

Figure S7. ^{1}H NMR spectrum of 1h.

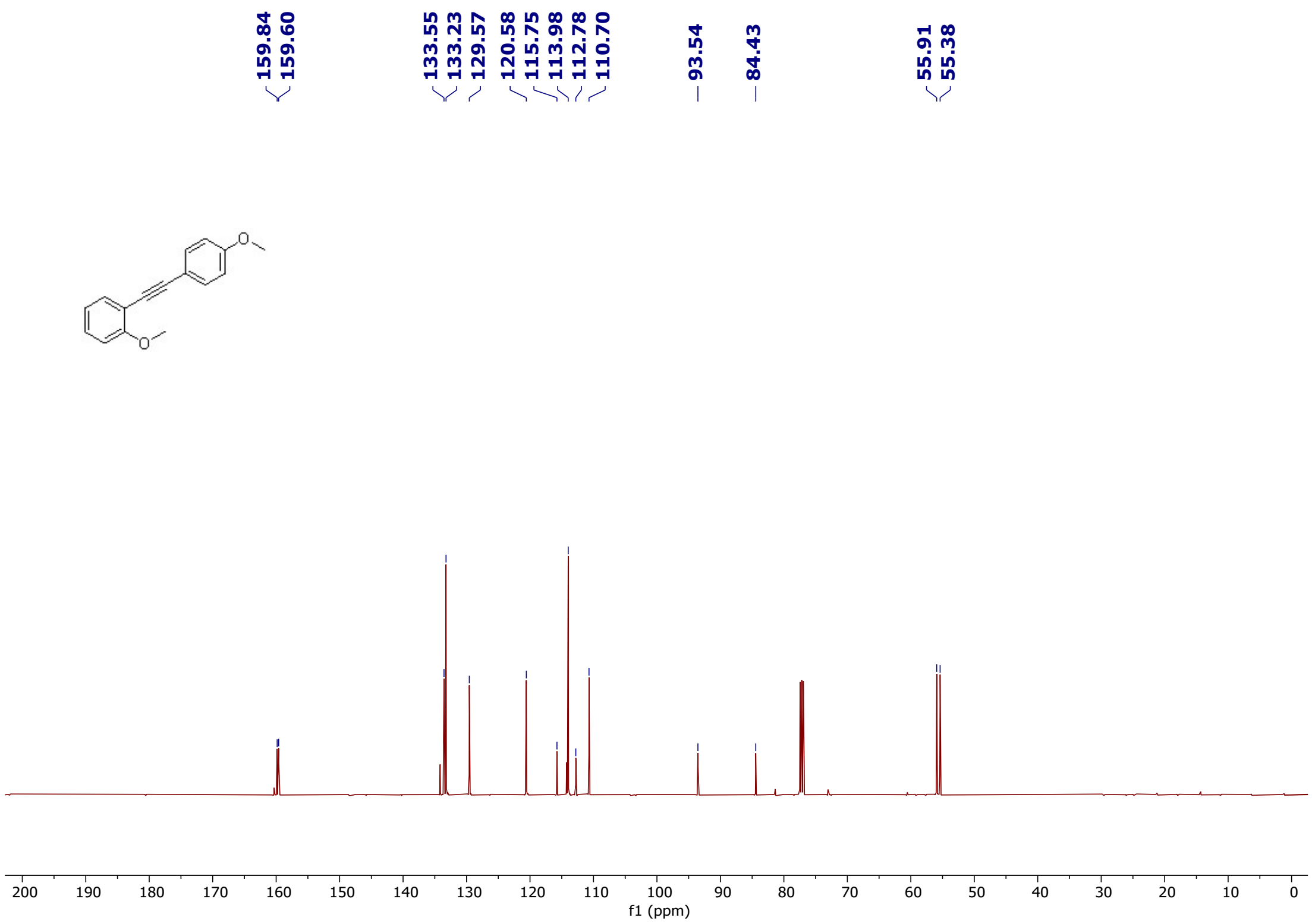

Figure S8. ^{13}C NMR spectrum of 1h.

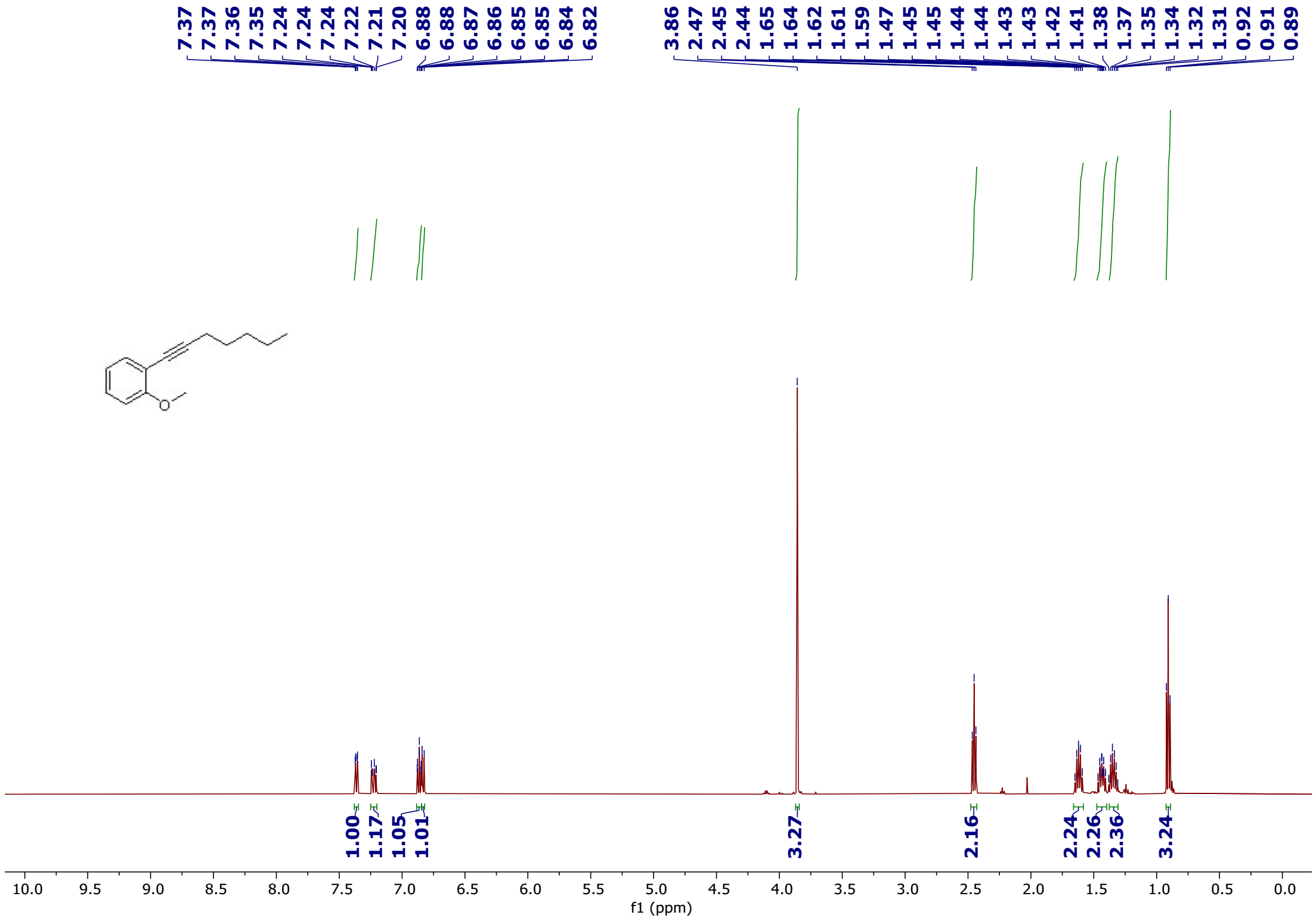

Figure S9. ^{1}H NMR spectrum of 1j.

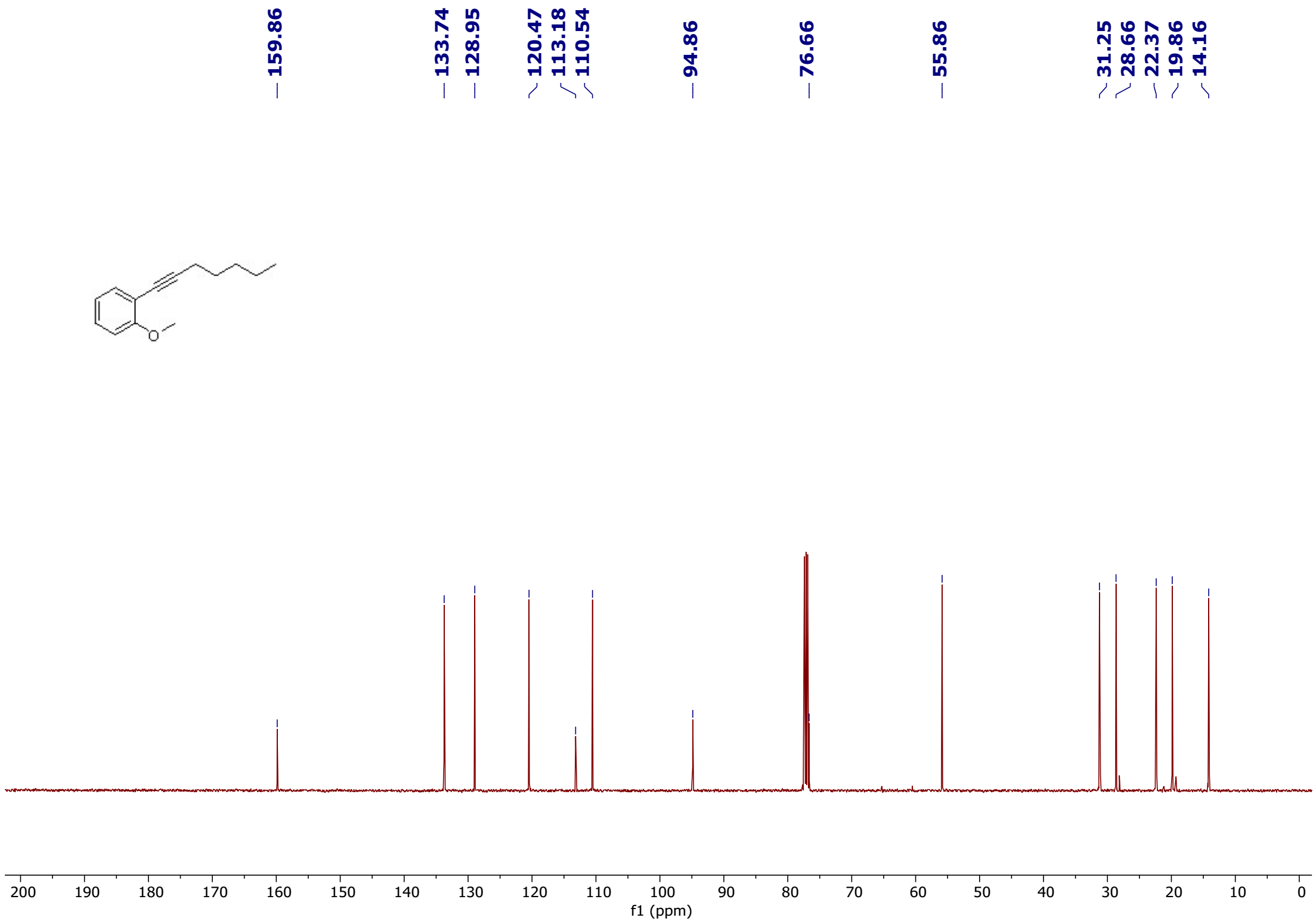

Figure S10. ^{13}C NMR spectrum of 1j.

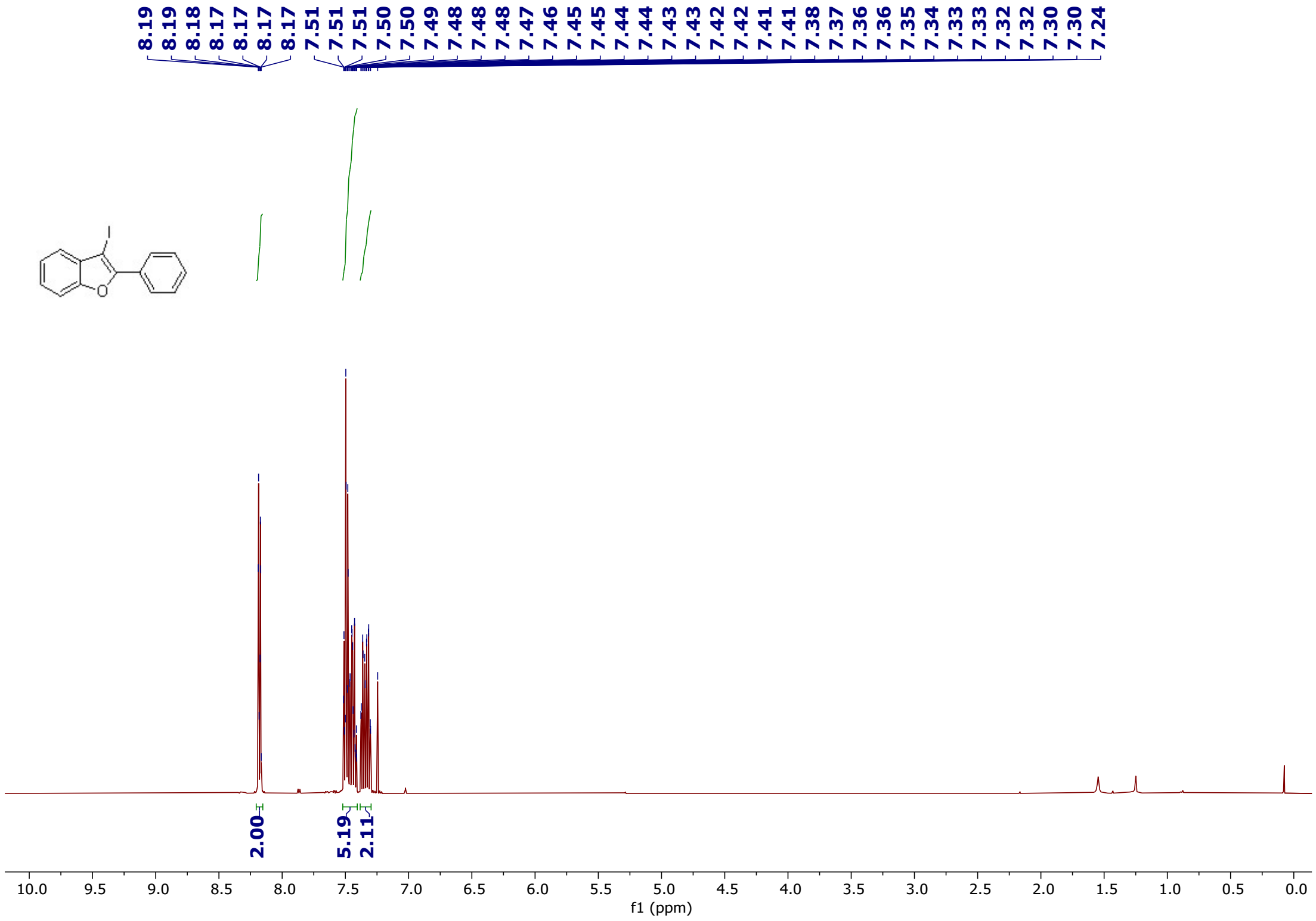

Figure S11. ^{1}H NMR spectrum of 2b.

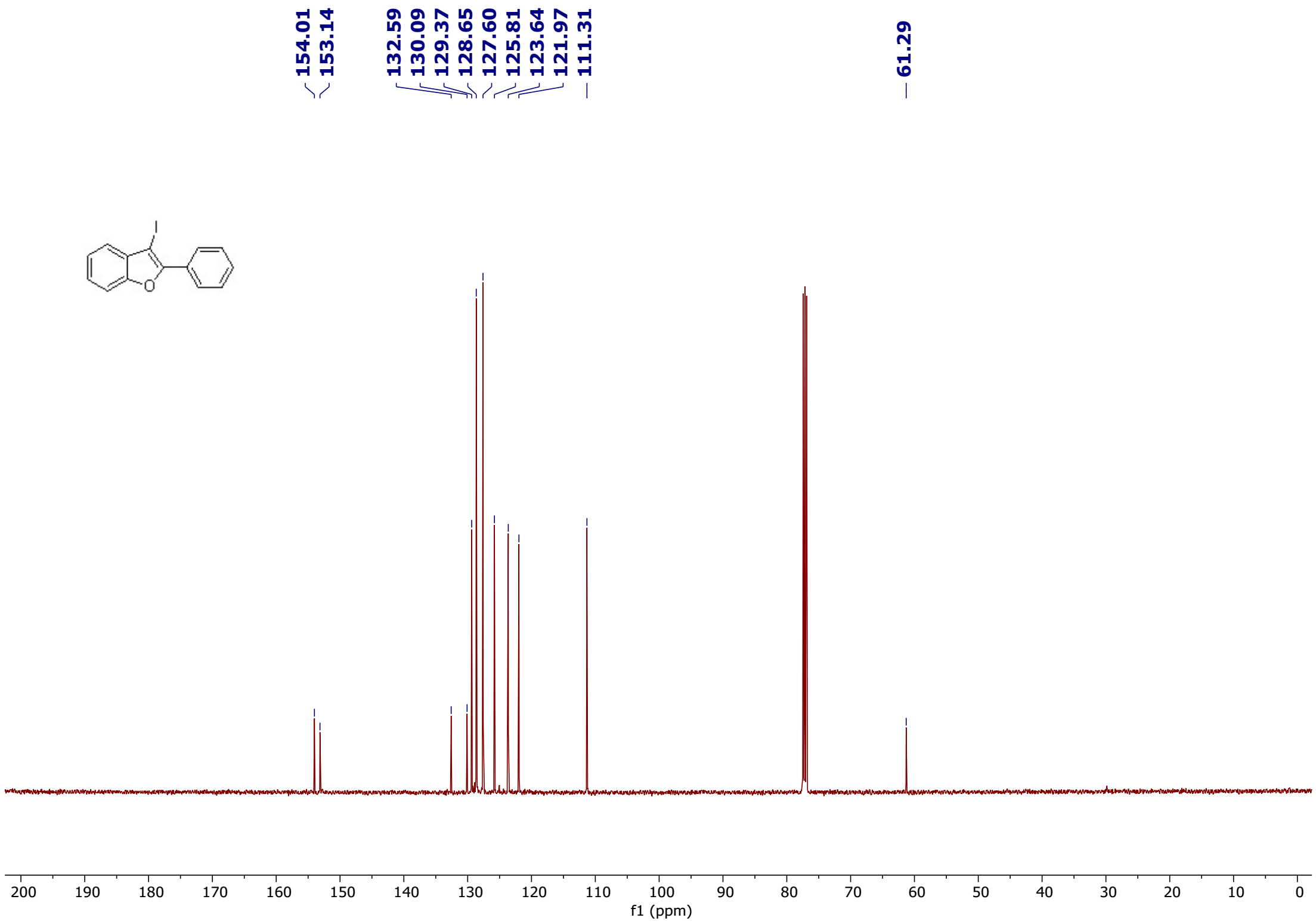

Figure S12. ^{13}C NMR spectrum of 2b.

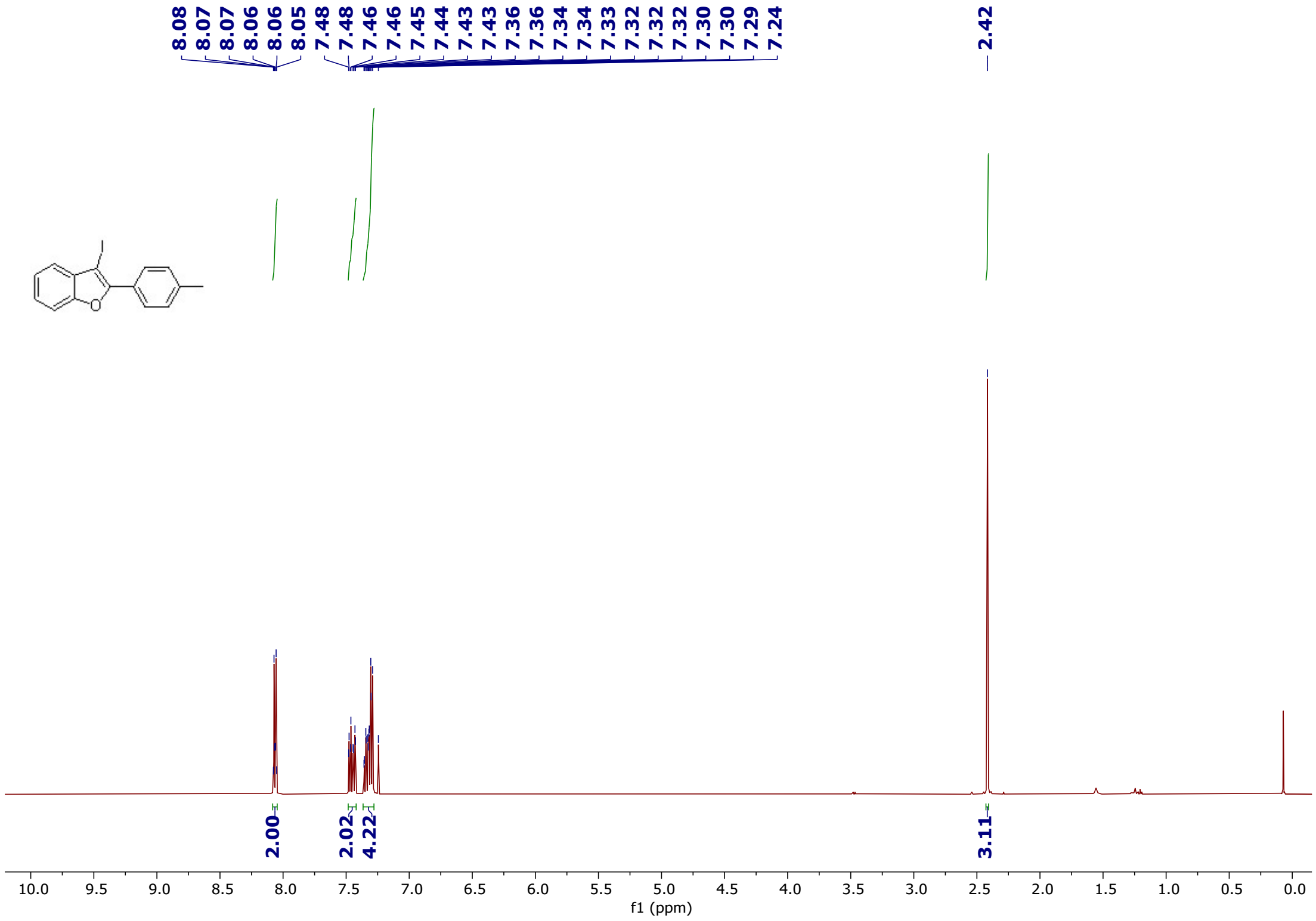

Figure S13. ^{1}H NMR spectrum of 2d.

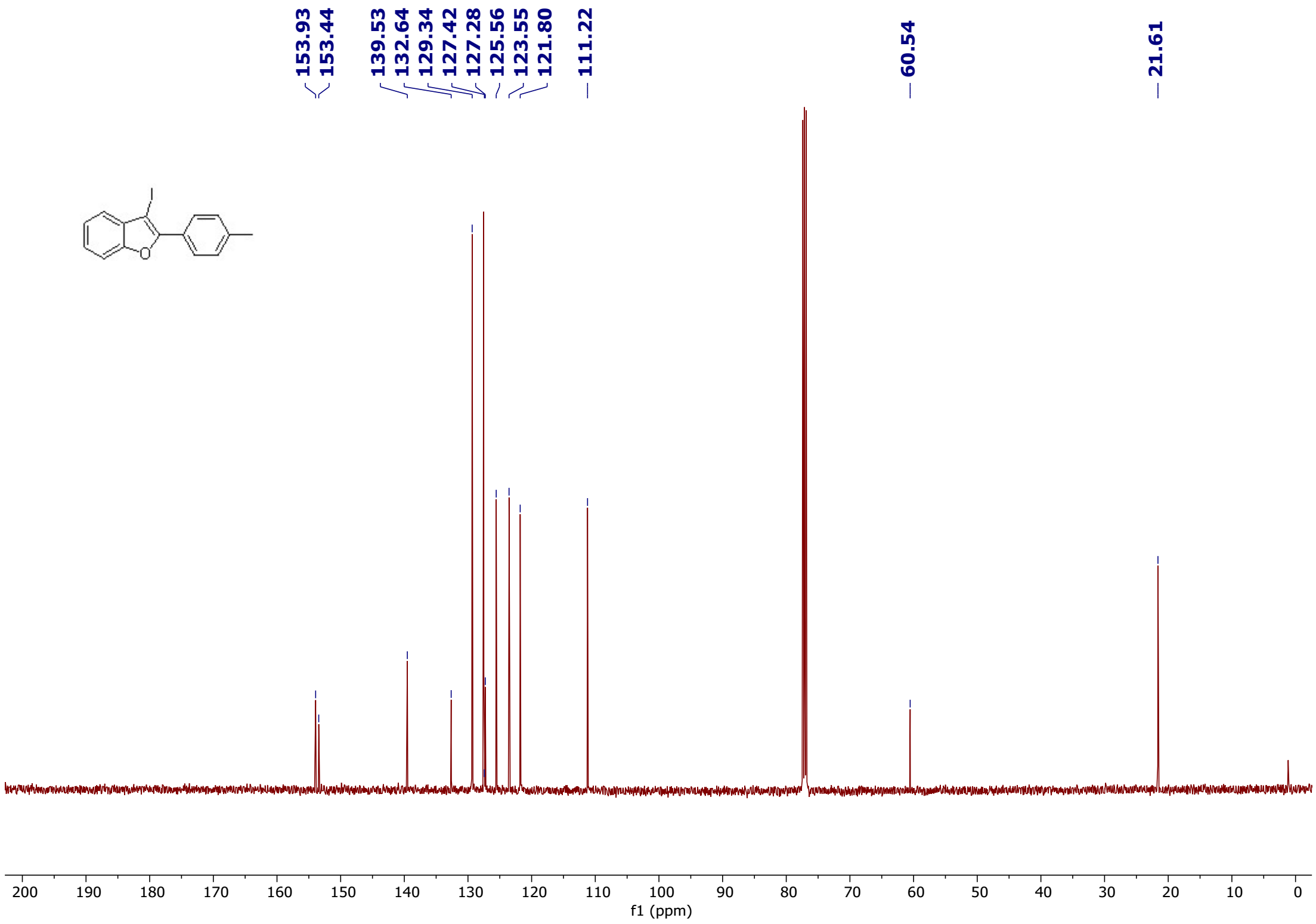

Figure S14. ^{13}C NMR spectrum of 2d.

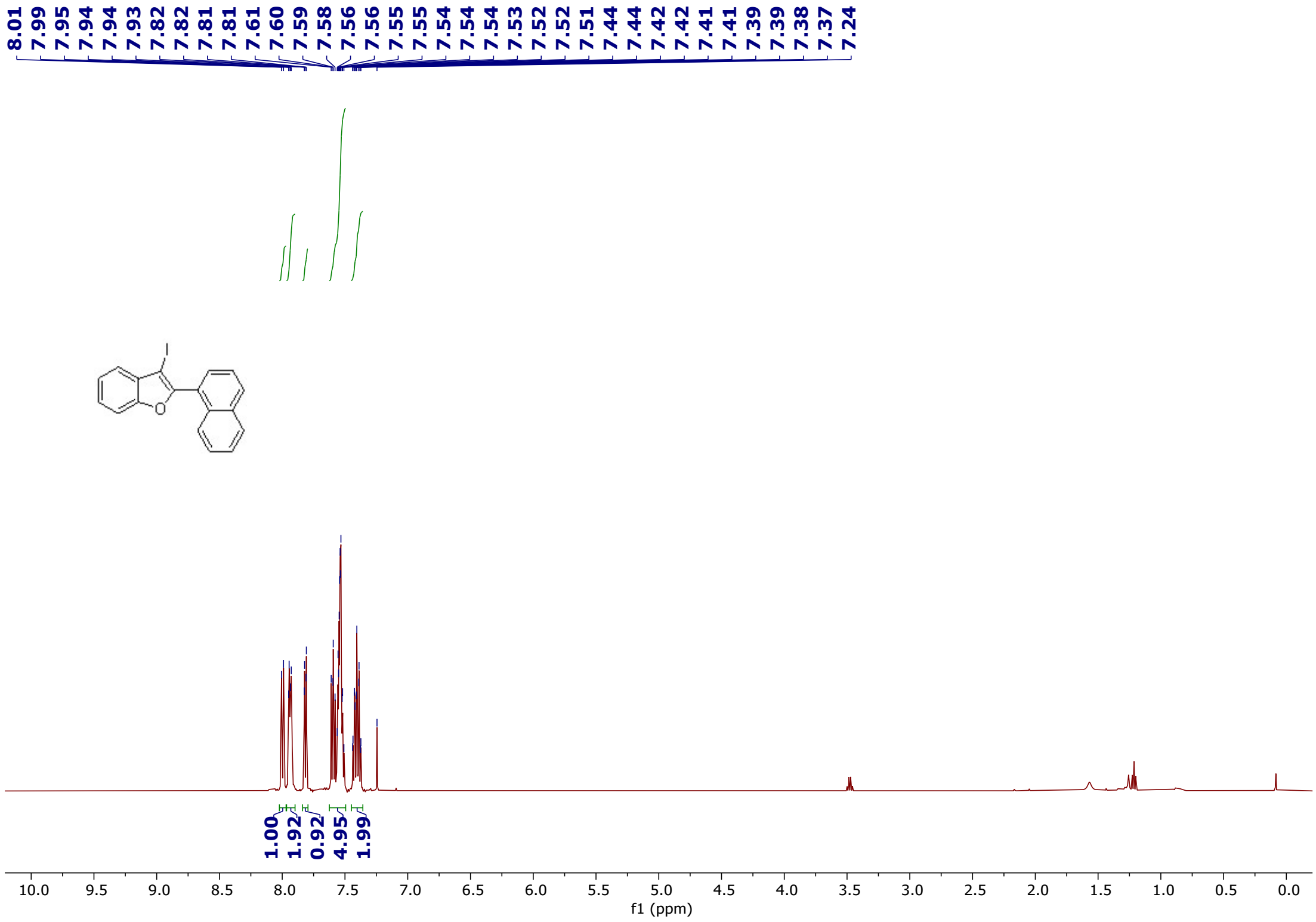

Figure S15. ^{1}H NMR spectrum of 2f.

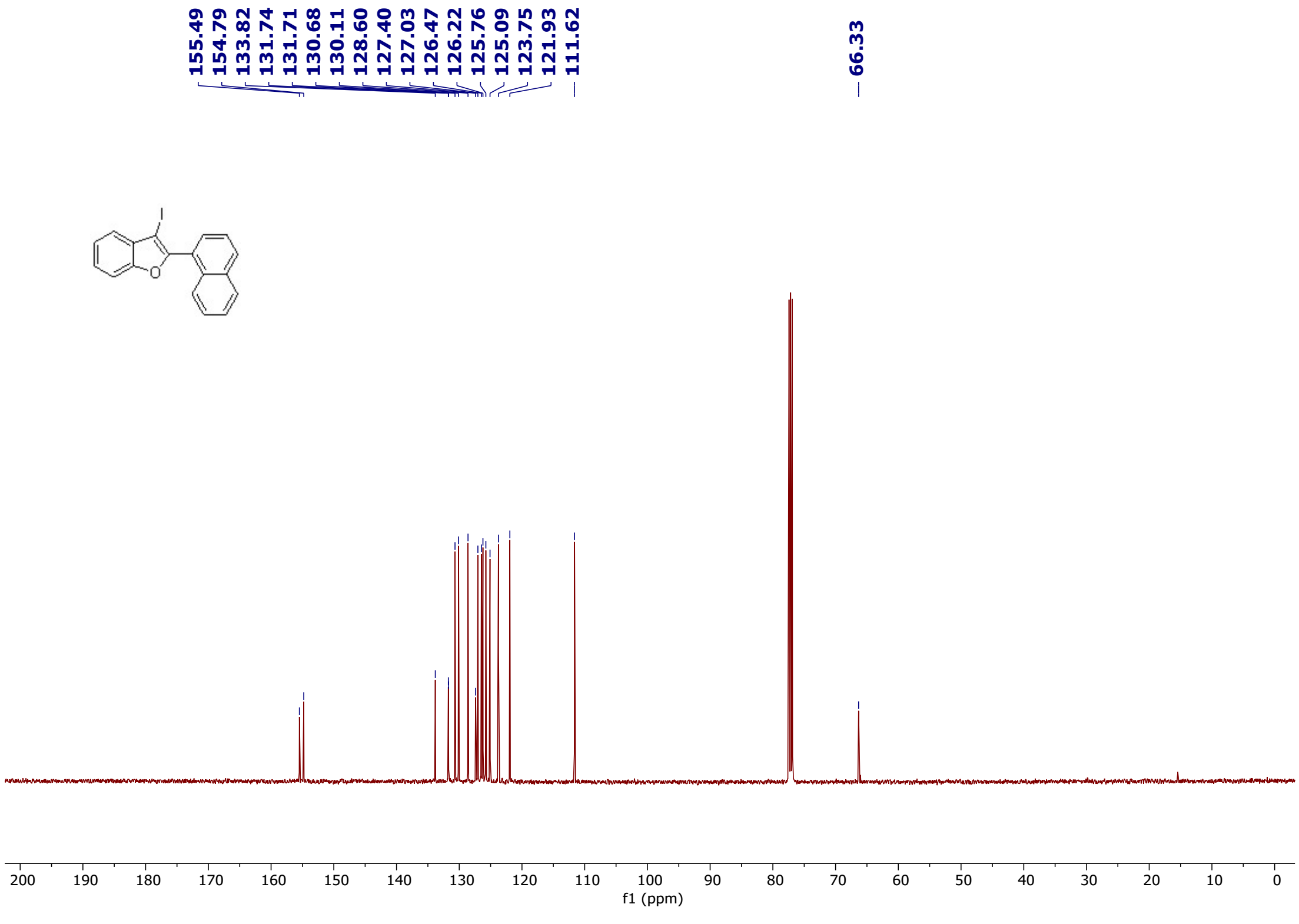

Figure S16. ^{13}C NMR spectrum of 2f.

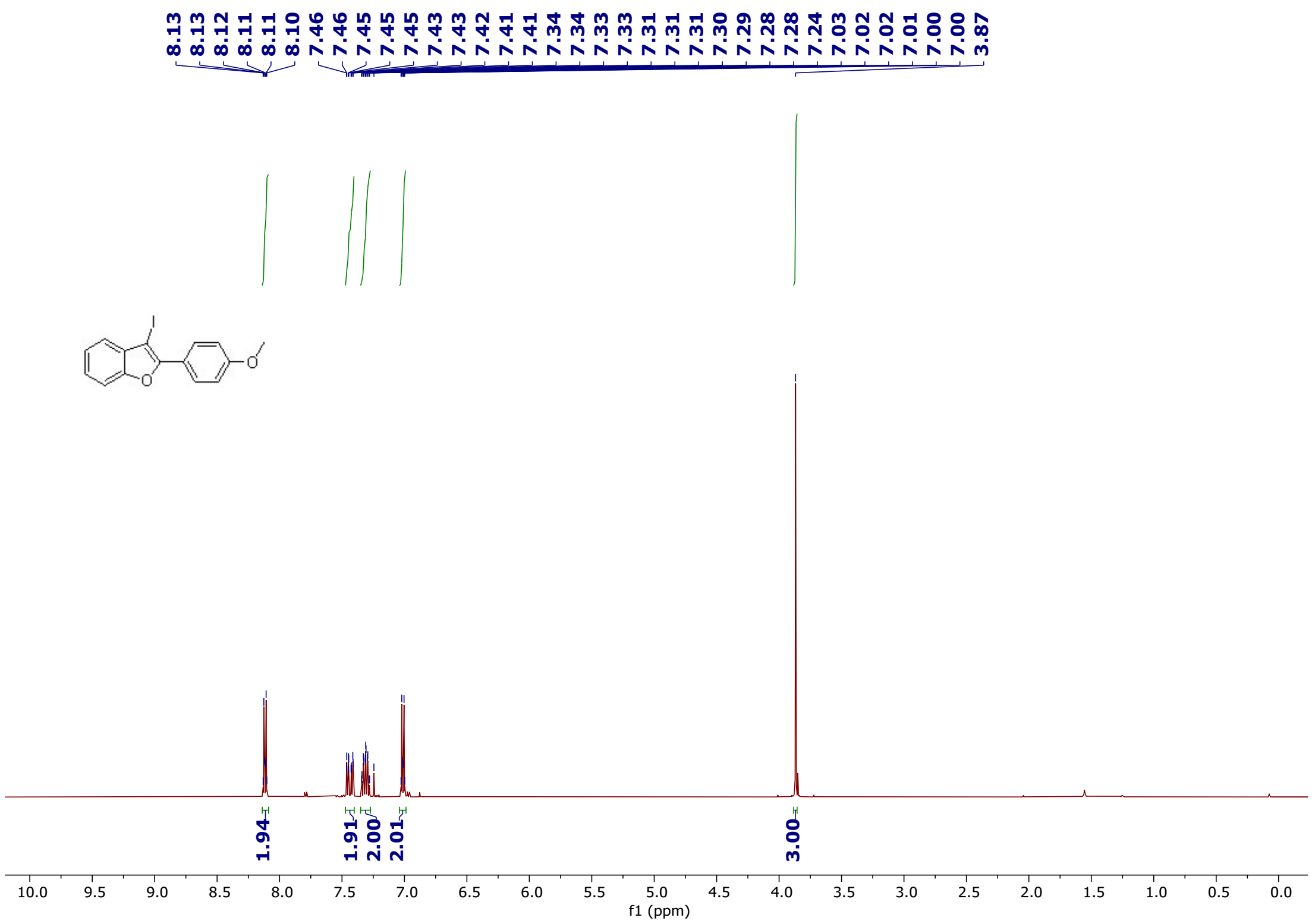

Figure S17. ^{1}H NMR spectrum of 2h.

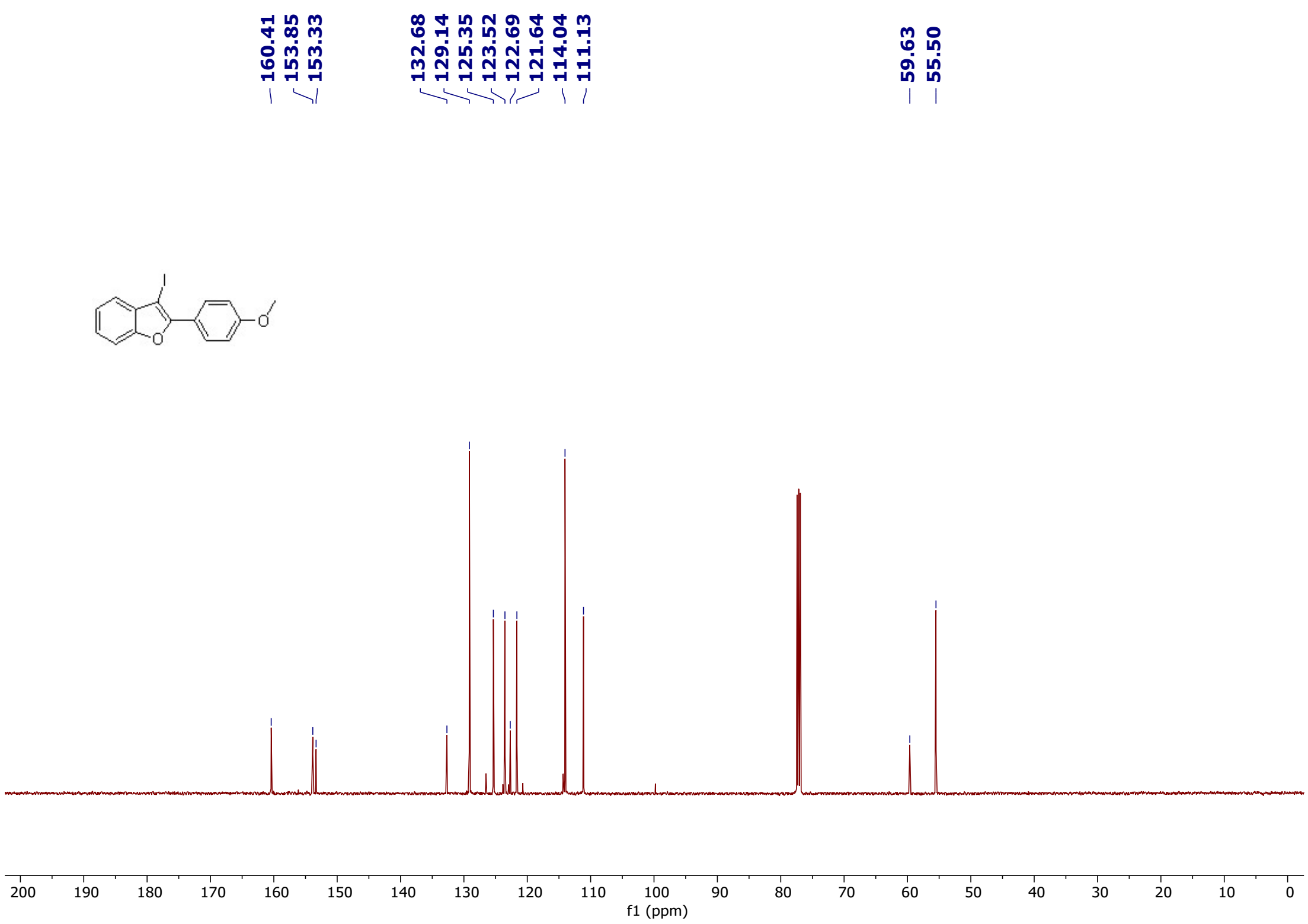

Figure S18. ^{13}C NMR spectrum of 2h.

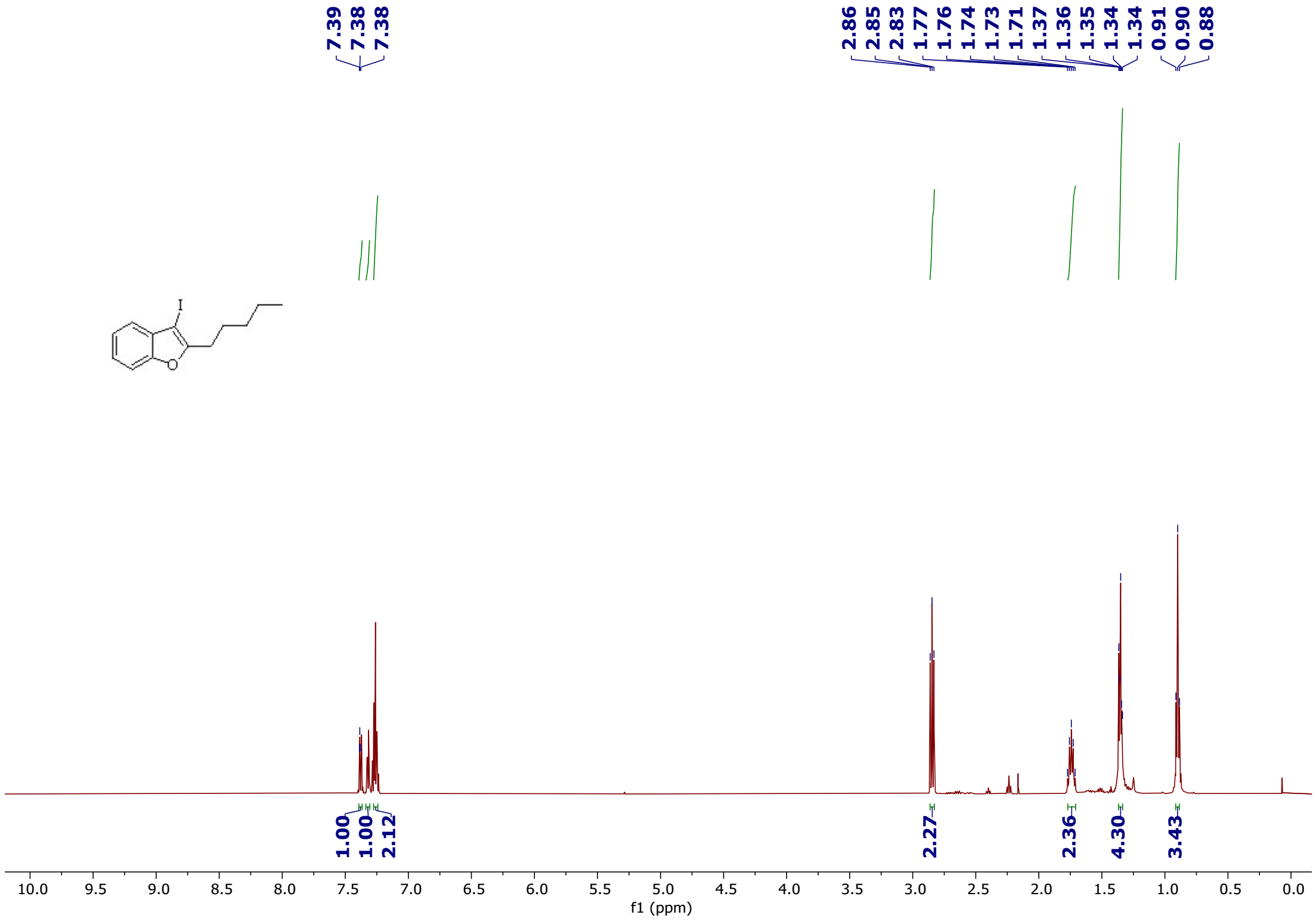

Figure S19. ^{1}H NMR spectrum of 2j.

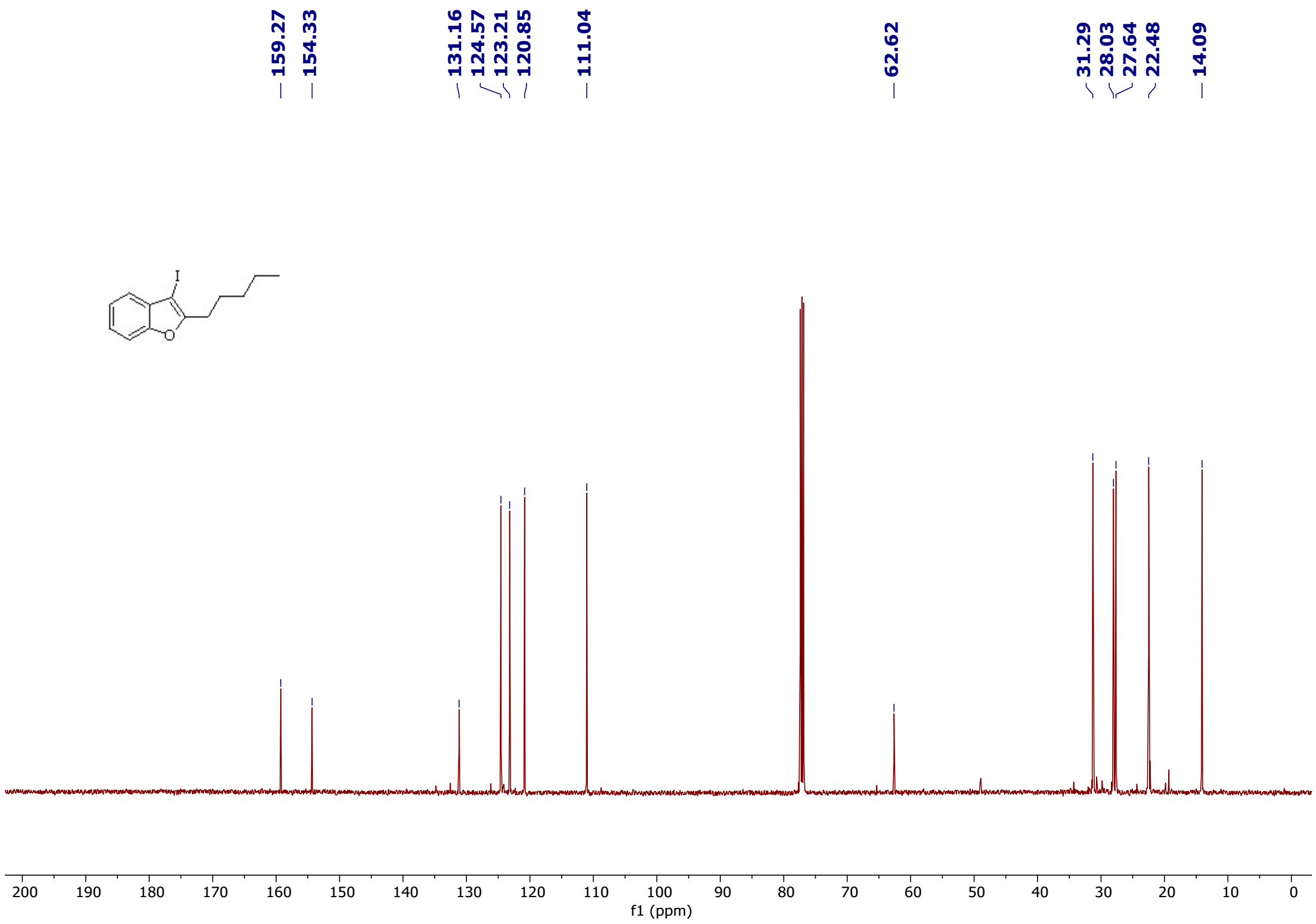

Figure S20. ^{13}C NMR spectrum of 2j.

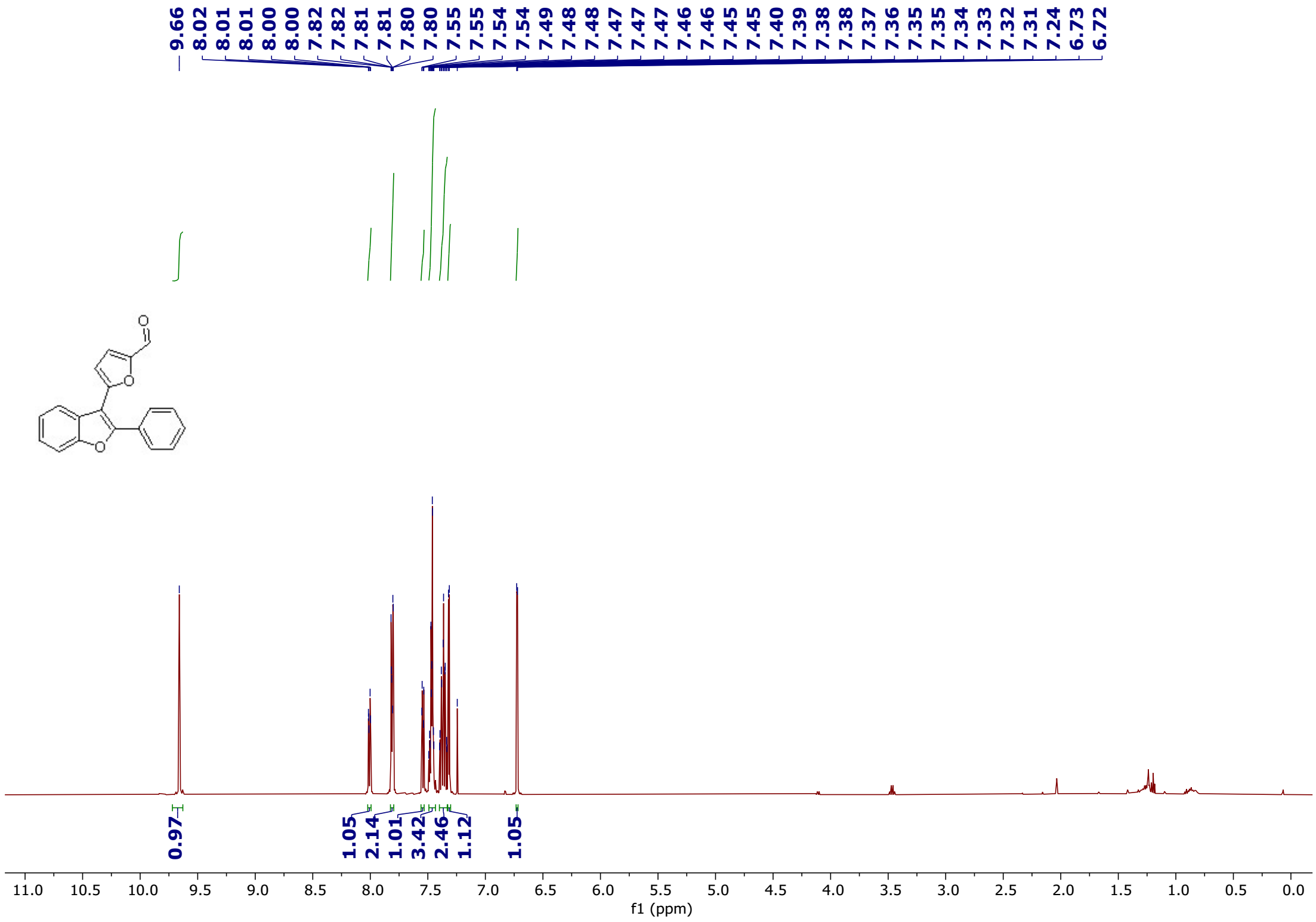

Figure S21. ^{1}H NMR spectrum of 3b.

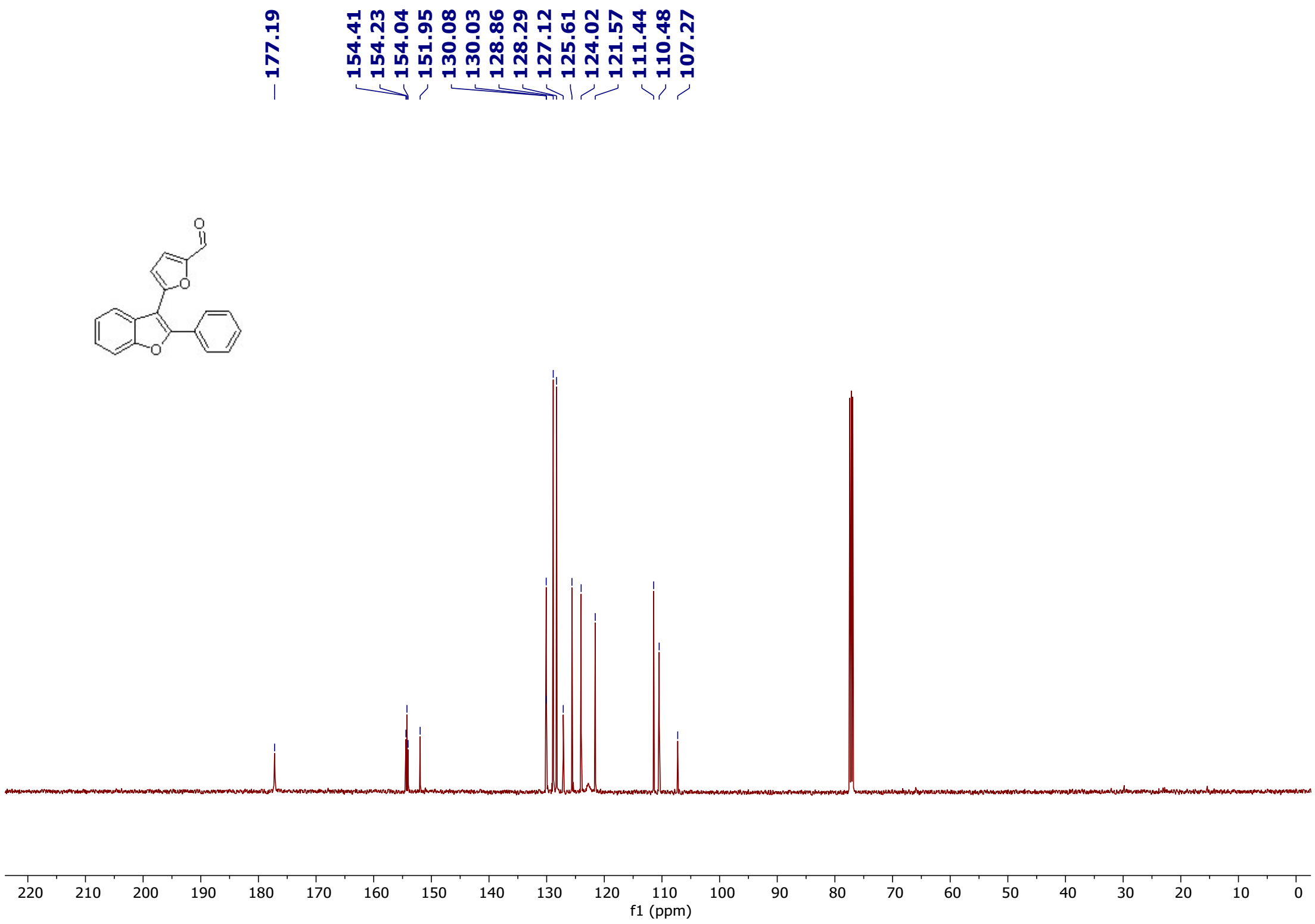

Figure S22. ^{13}C NMR spectrum of 3b.

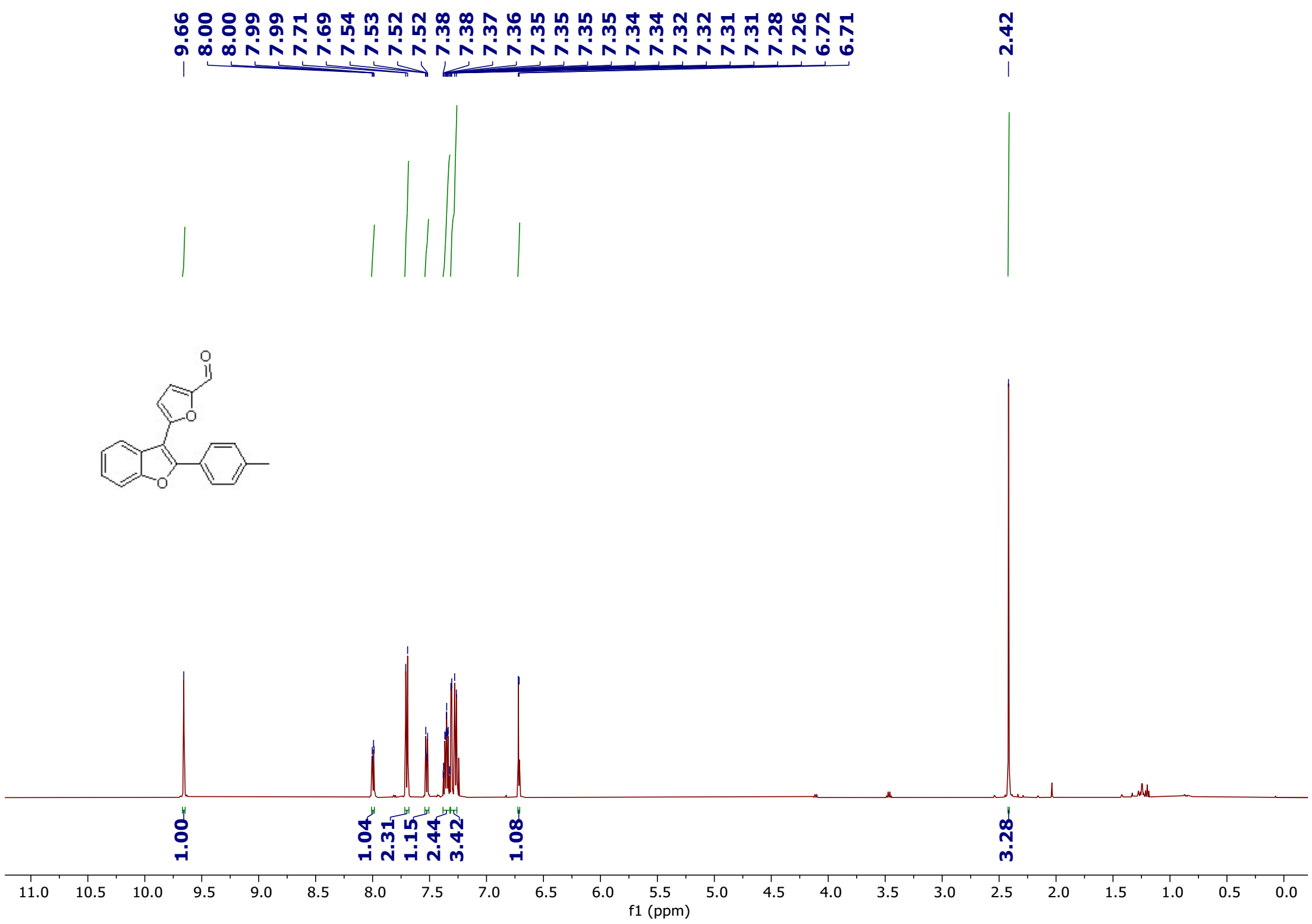

Figure S23. ^{1}H NMR spectrum of 3d.

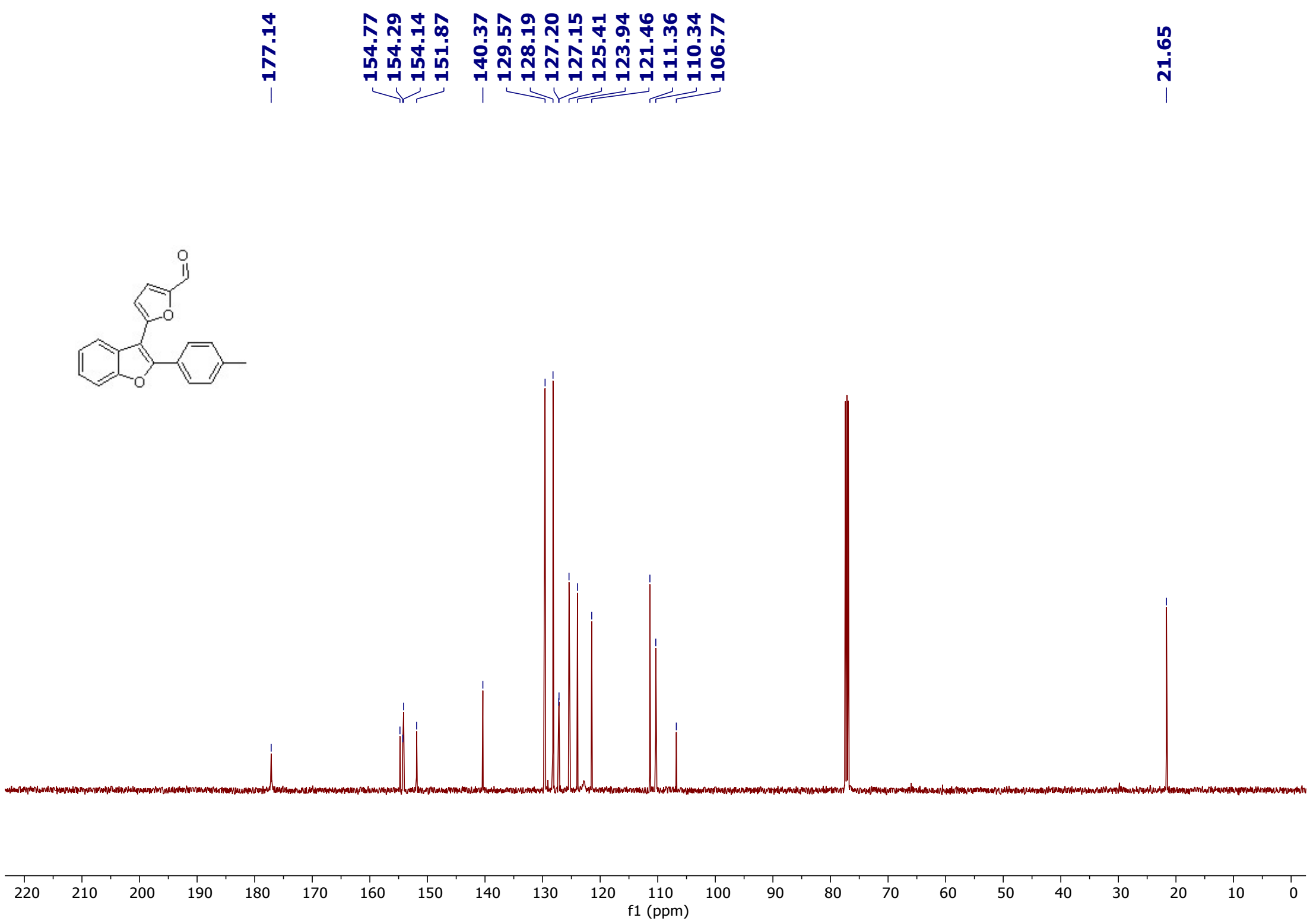

Figure S24. ^{13}C NMR spectrum of 3d.

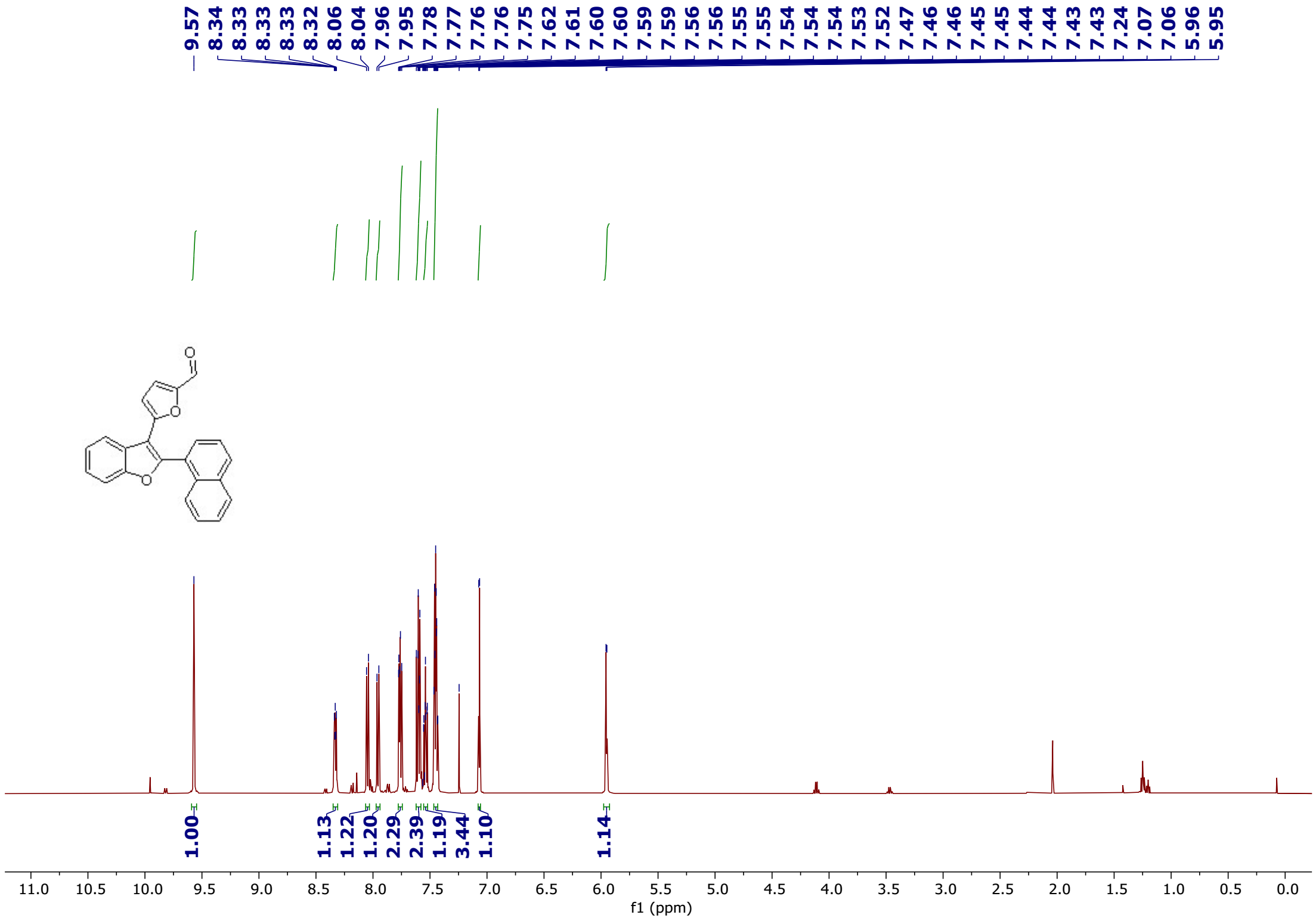

Figure S25. ^{1}H NMR spectrum of 3f.

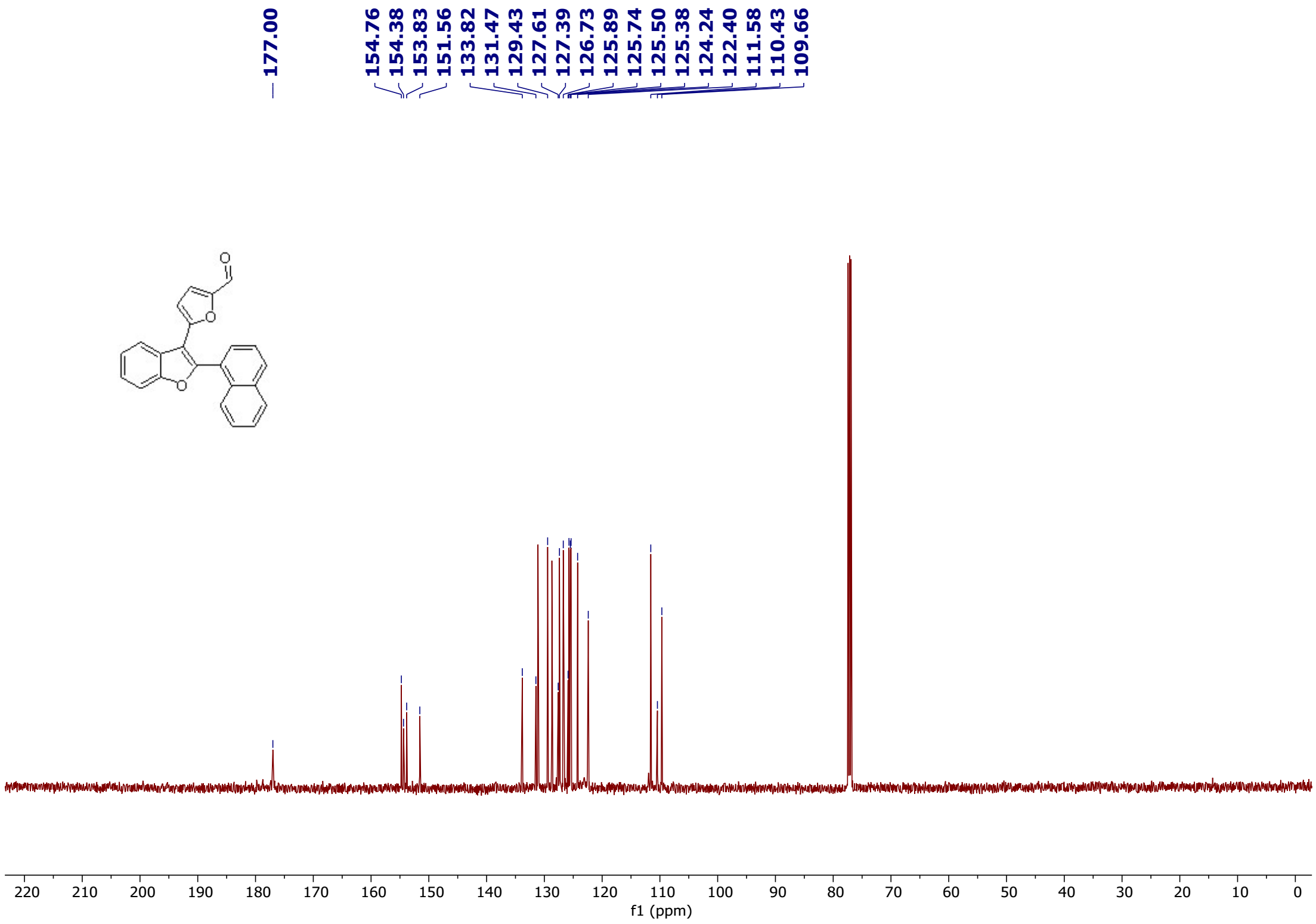

Figure S26. ^{13}C NMR spectrum of 3f.

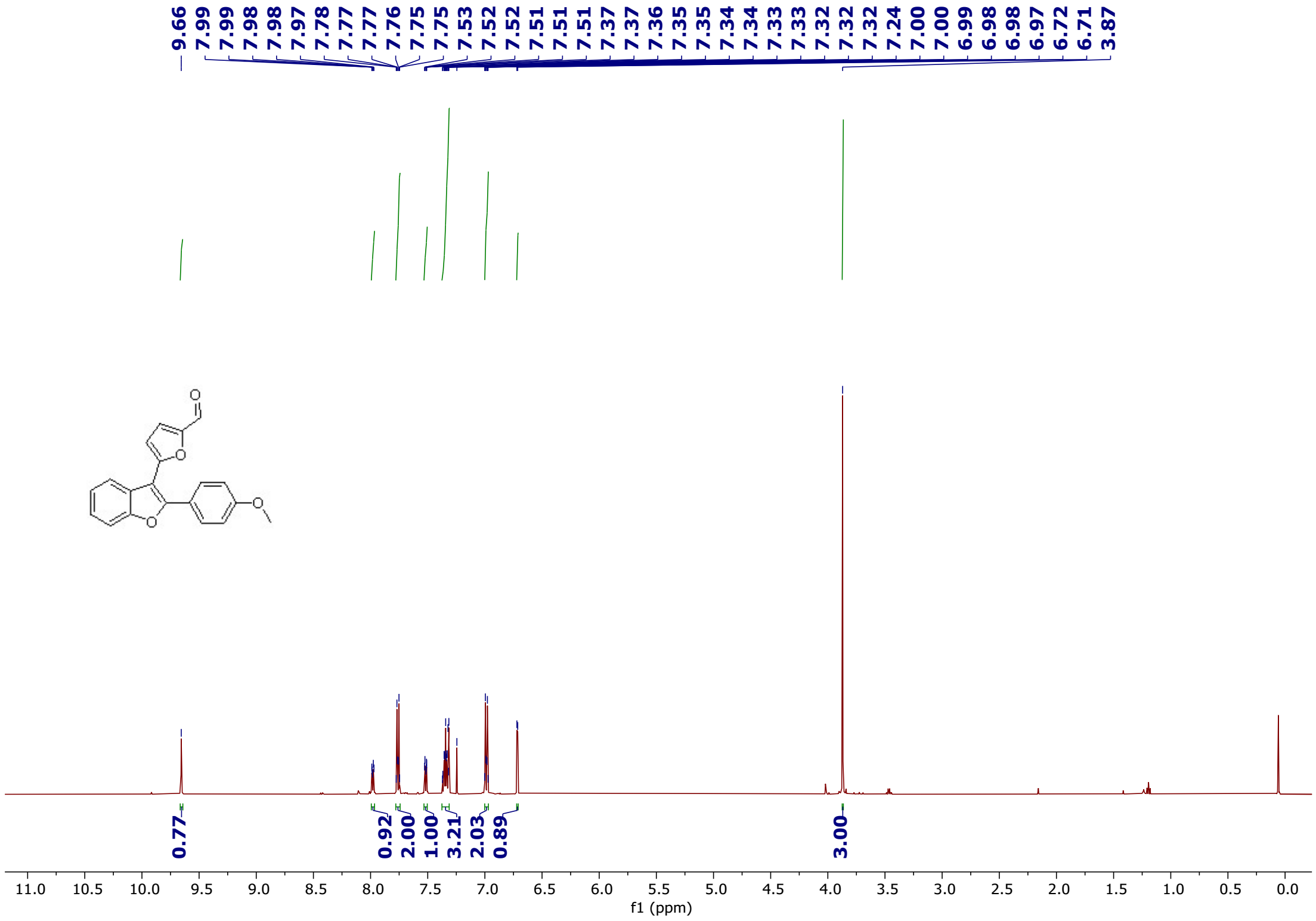

Figure S27. ^{1}H NMR spectrum of 3h.

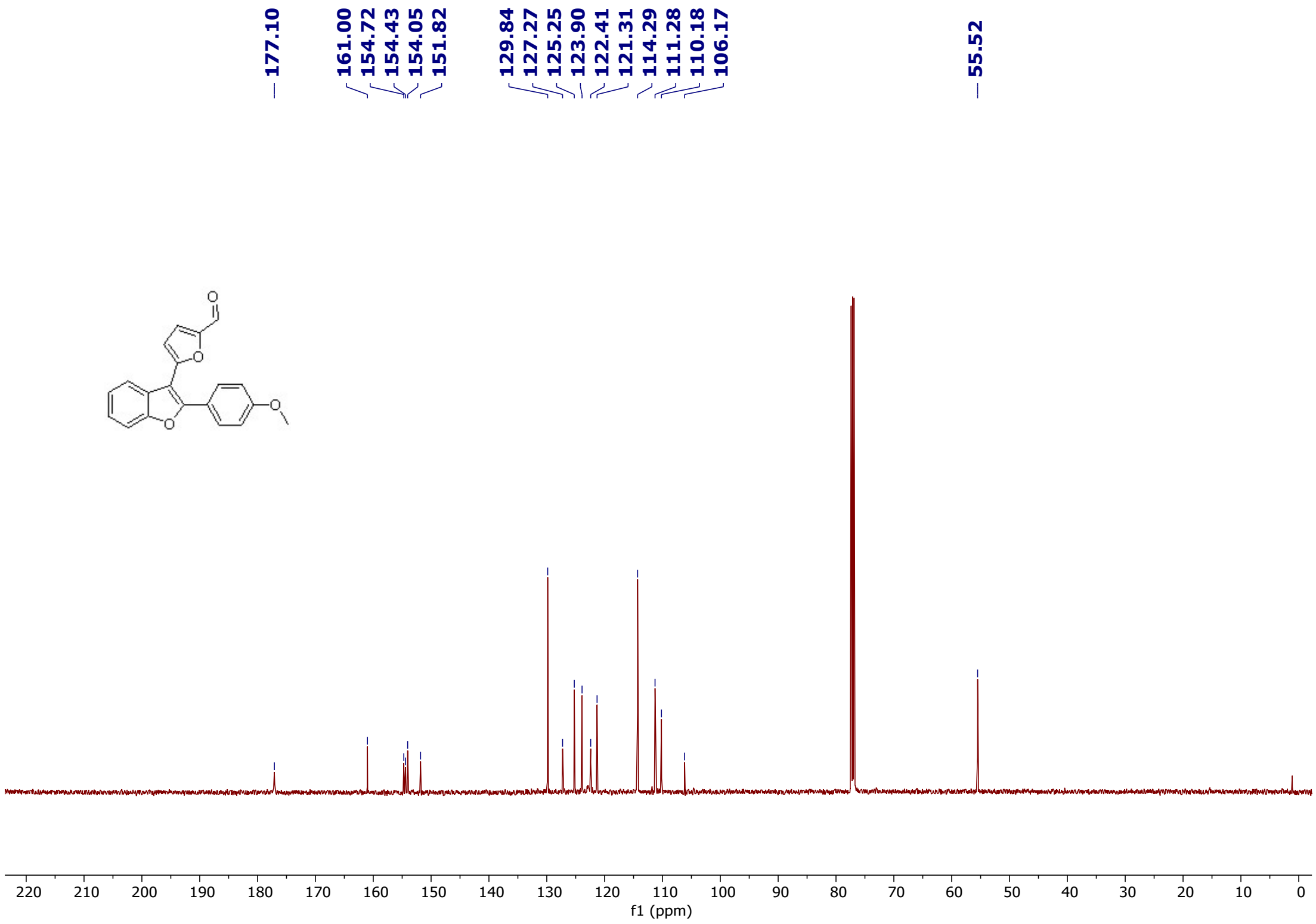

Figure S28. ^{13}C NMR spectrum of 3h.

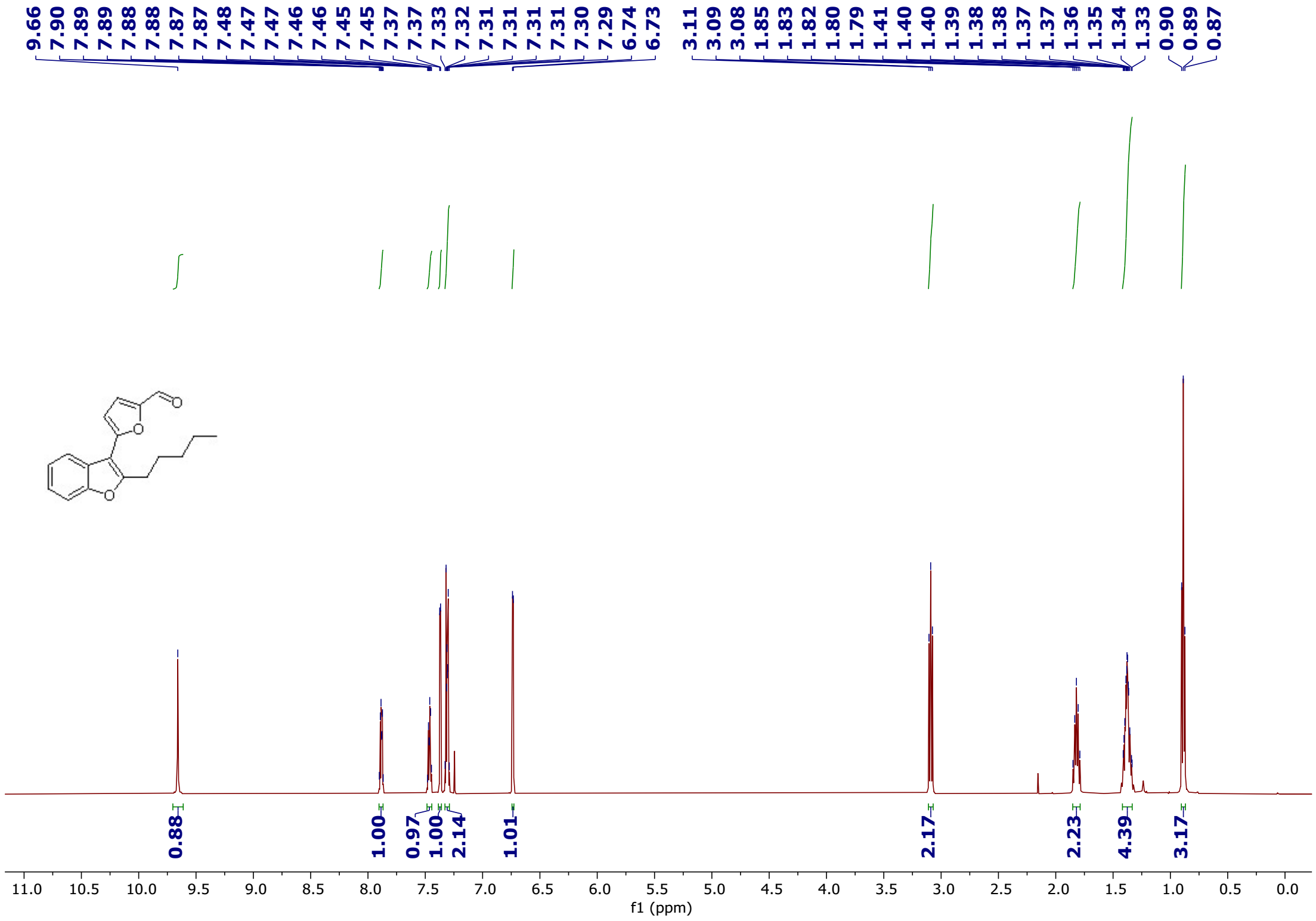

Figure S29. ^{1}H NMR spectrum of 3j.

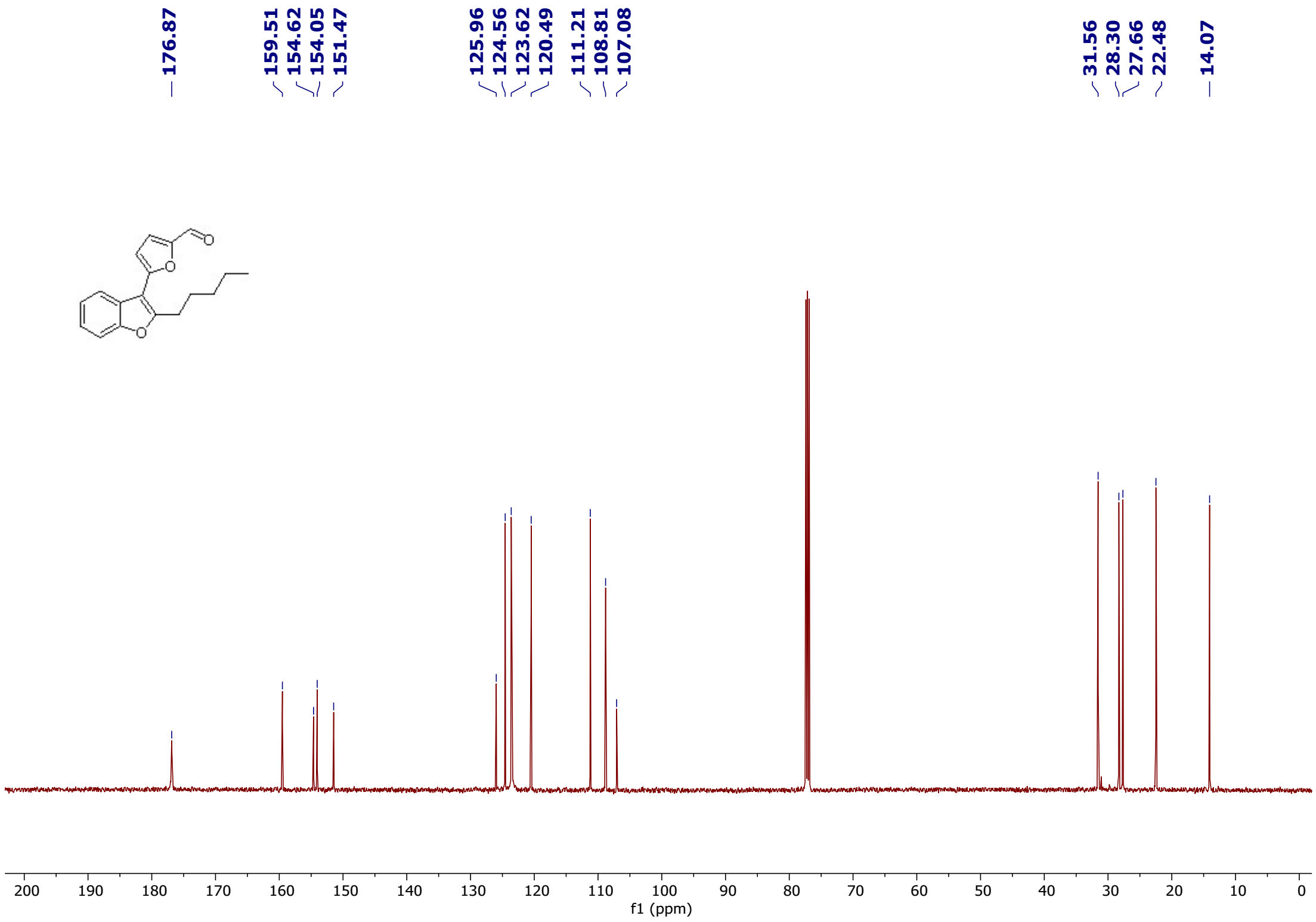

Figure S30. ^{13}C NMR spectrum of 3j.

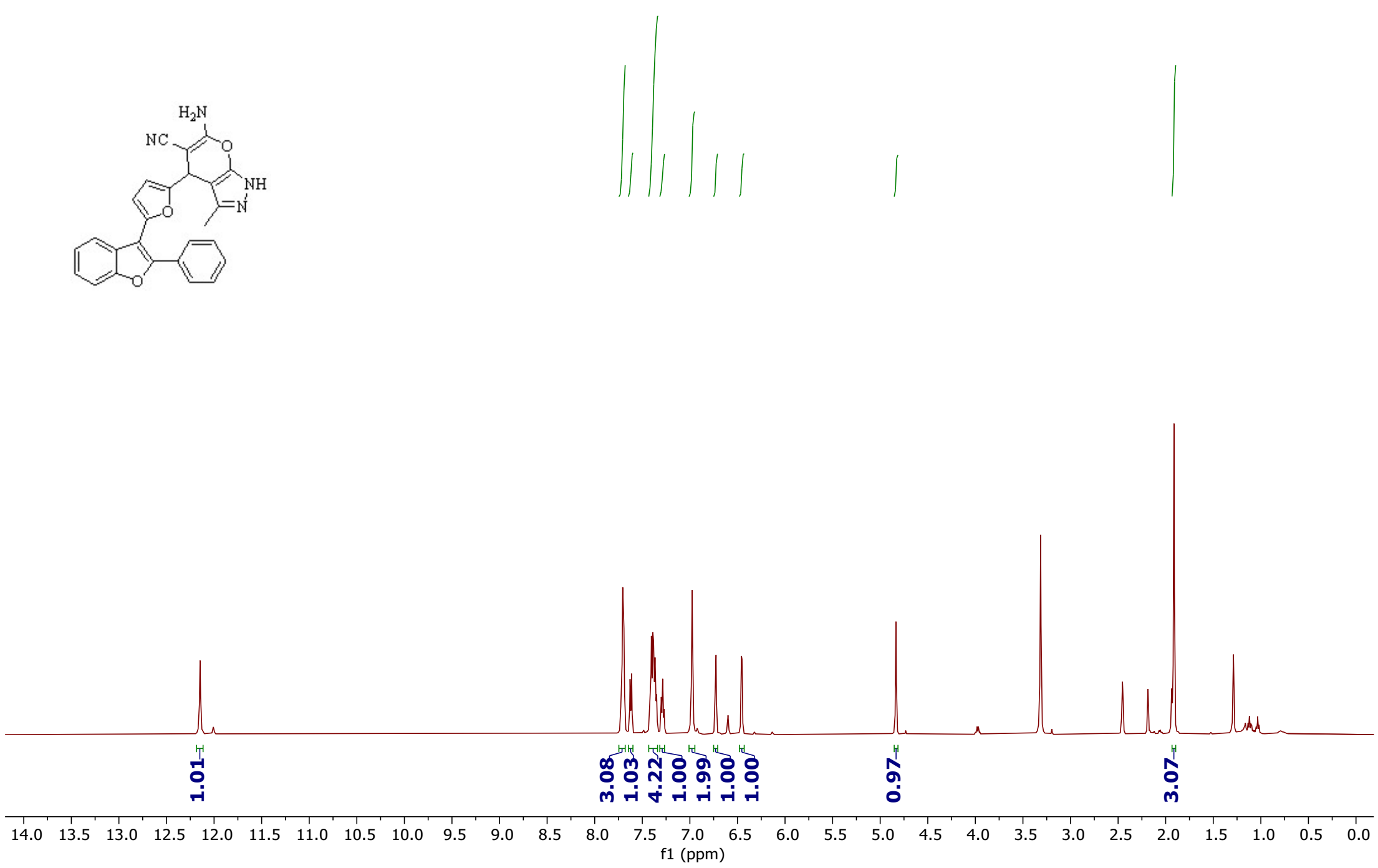

Figure S31. ^{1}H NMR spectrum of **4b**.

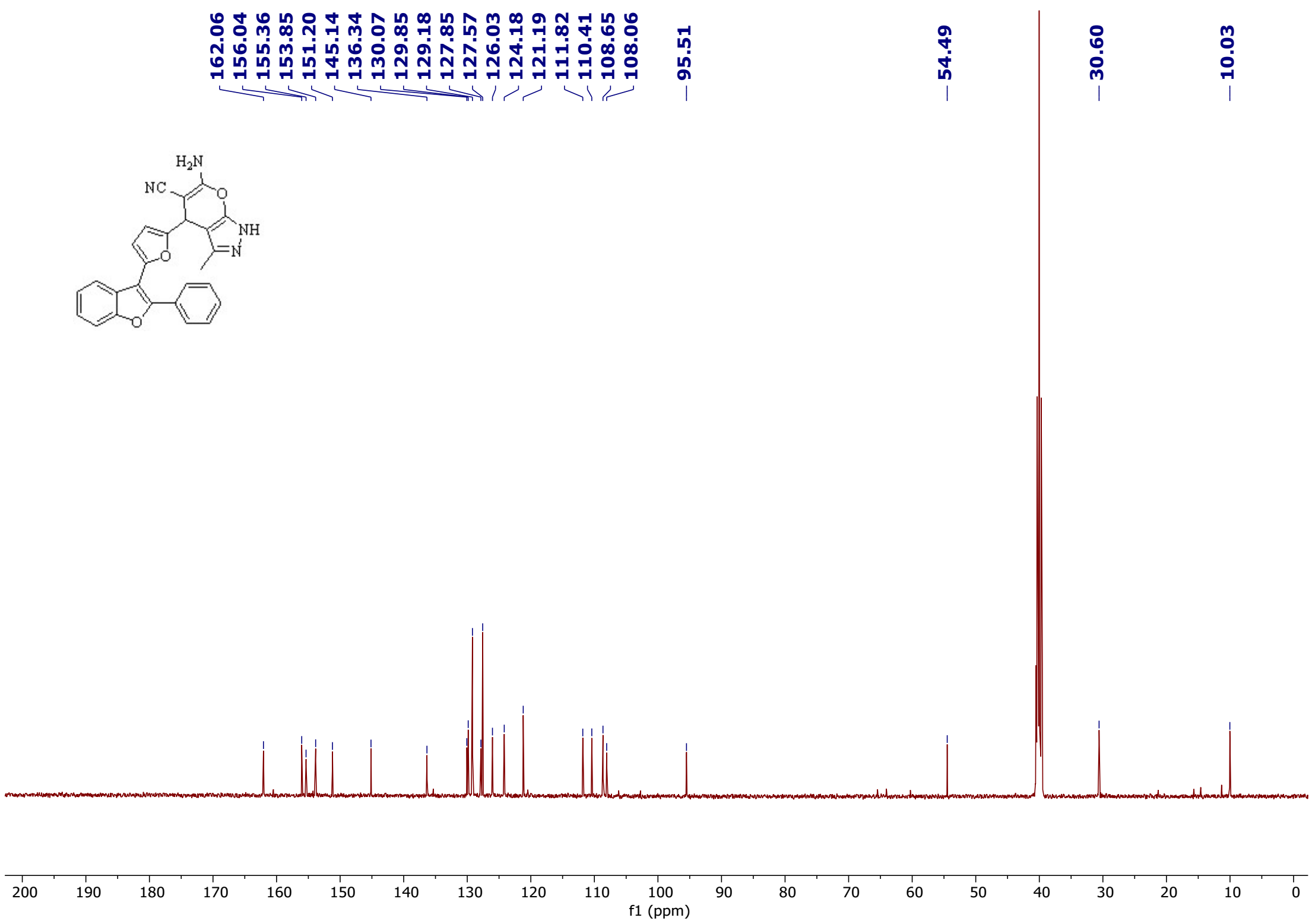

Figure S32. ^{13}C NMR spectrum of **4b**.

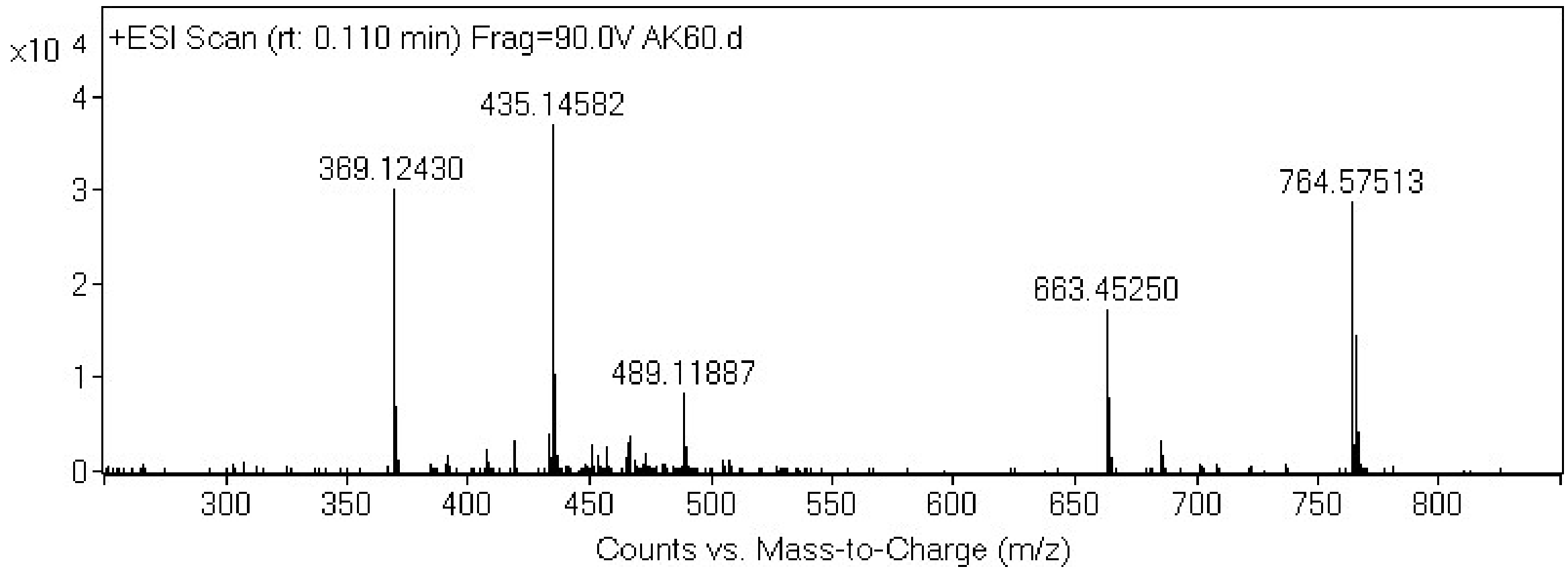

Figure S33. Mass spectrum of compound **4b**.

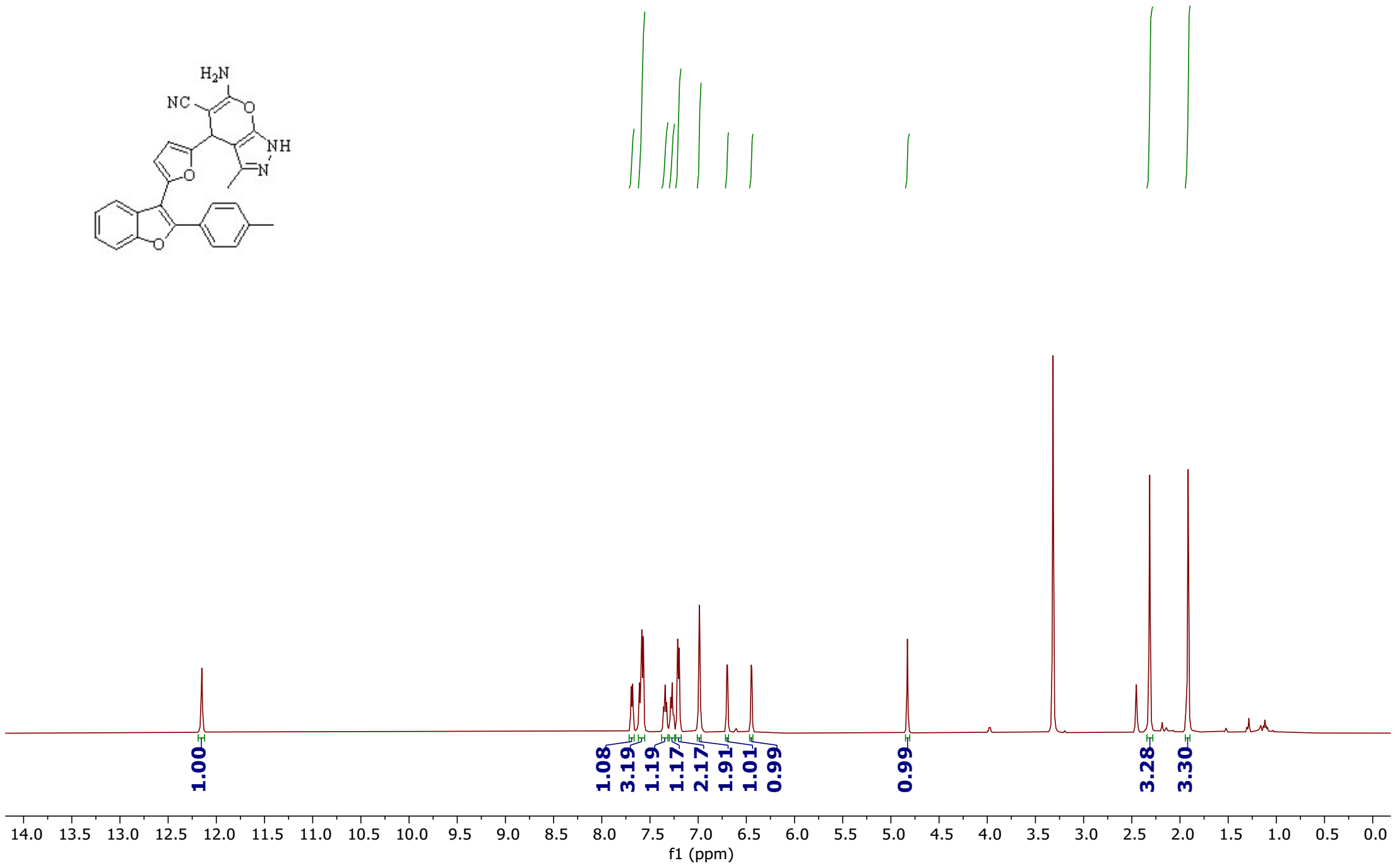

Figure S34. ^{1}H NMR spectrum of **4d**.

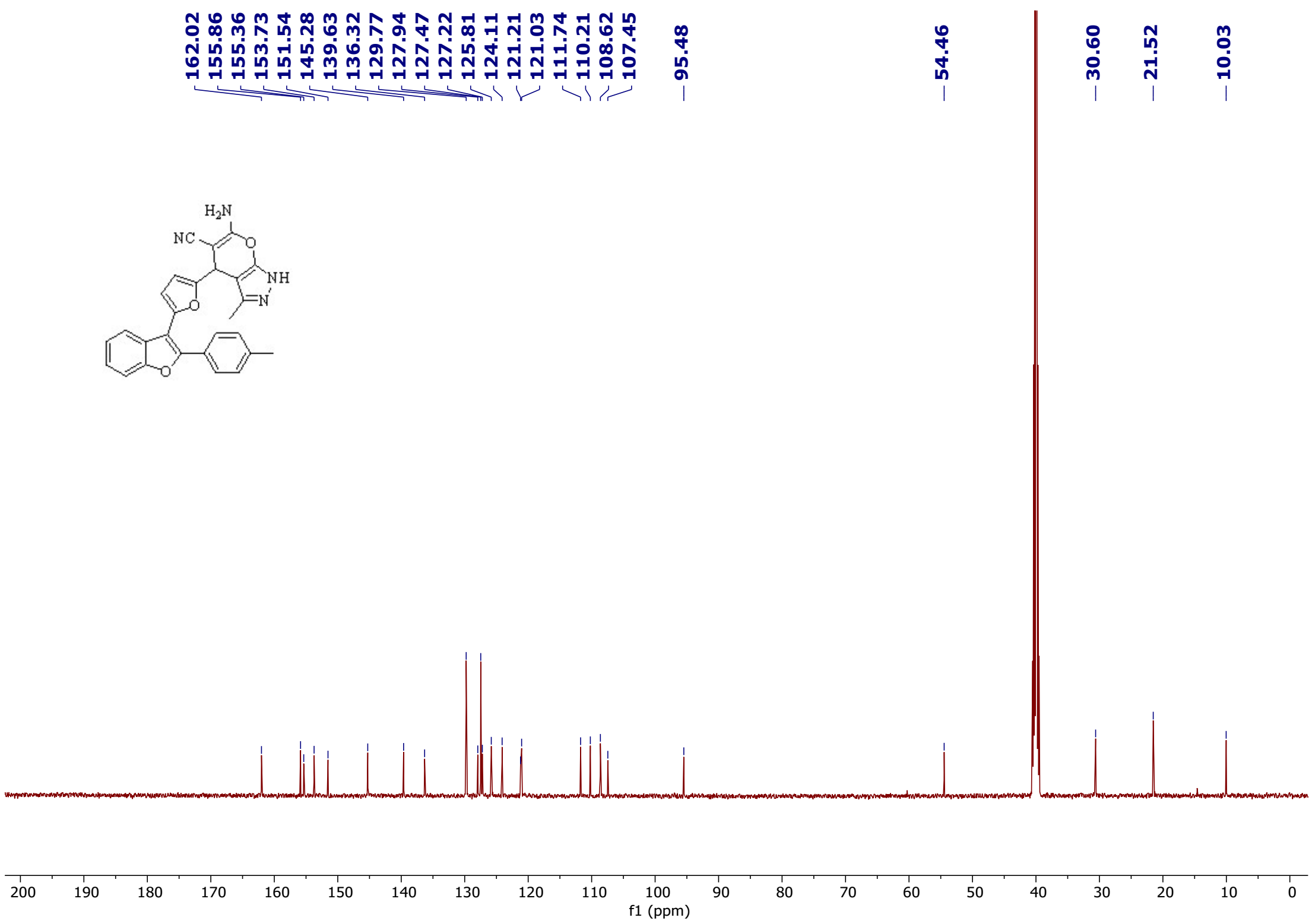

Figure S35. ^{13}C NMR spectrum of **4d**.

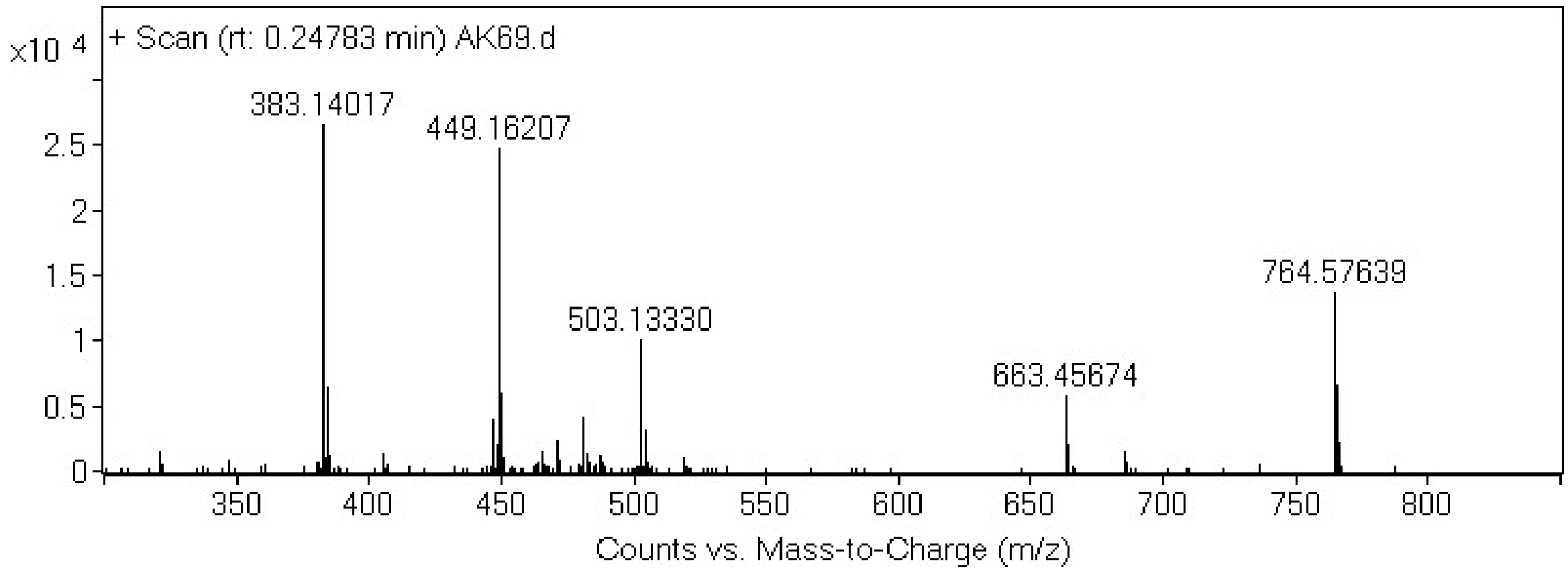

Figure S36. Mass spectrum of compound **4d**.

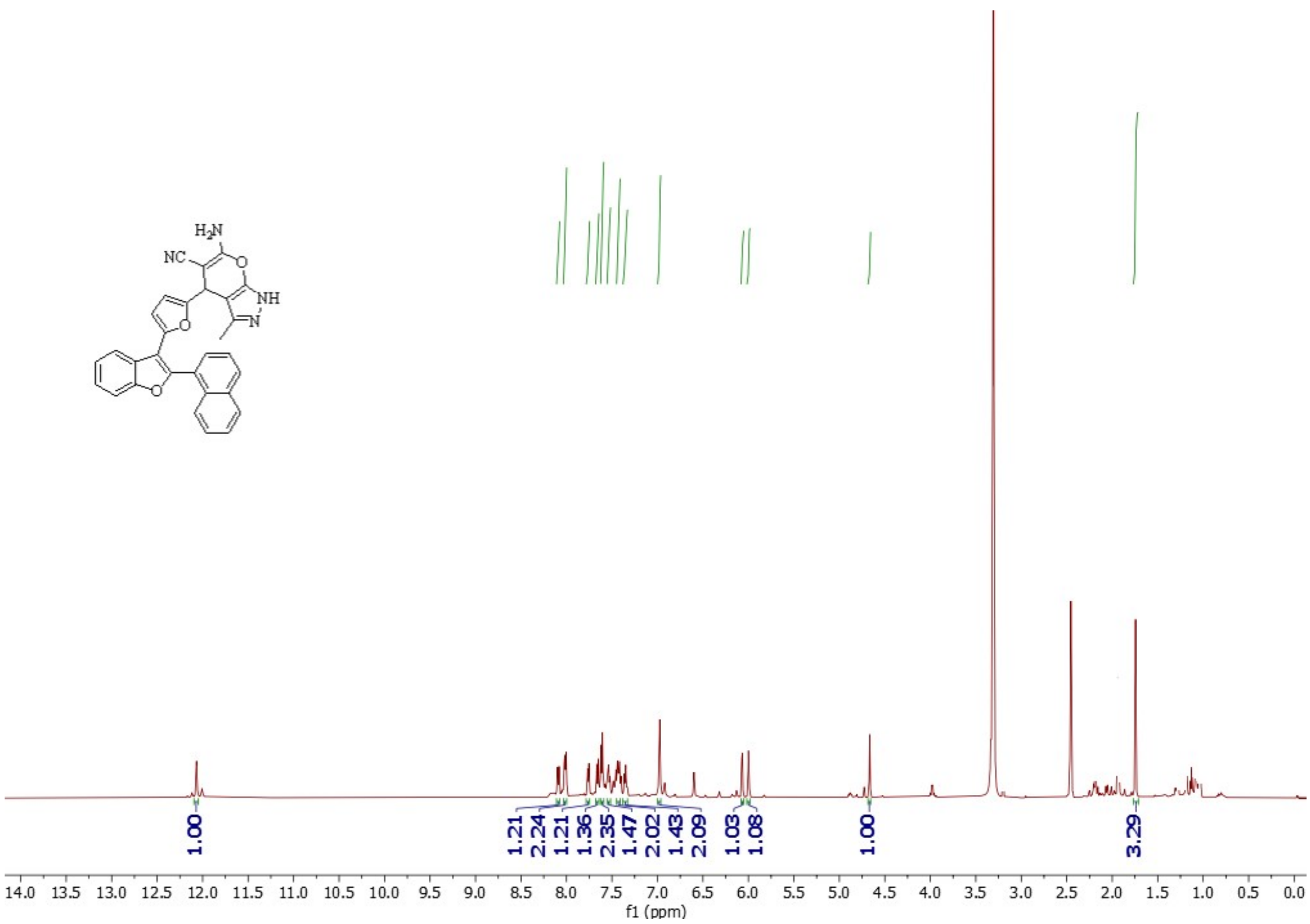

Figure S37. ^{1}H NMR spectrum of **4f**.

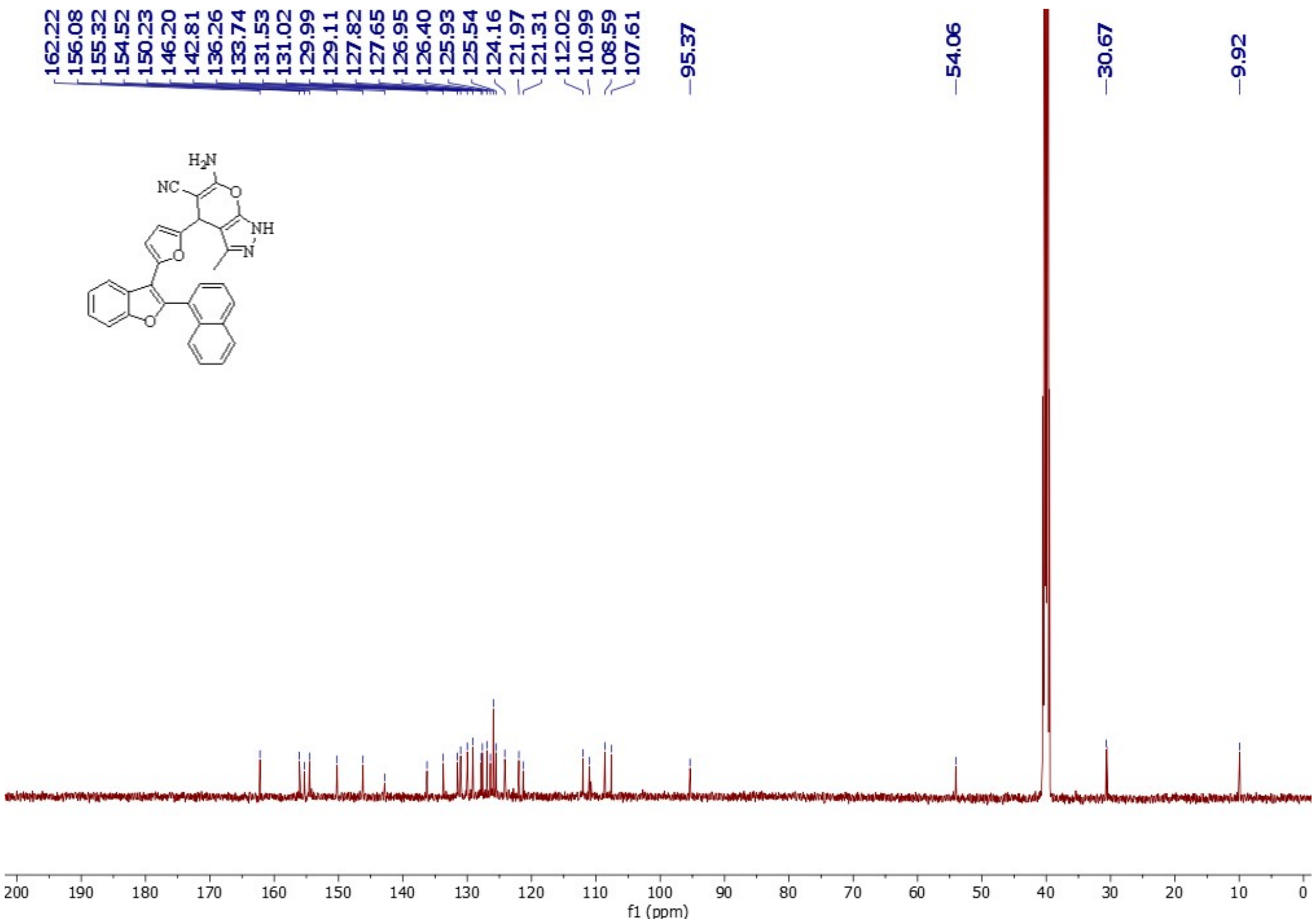

Figure S38. ^{13}C NMR spectrum of **4f**.

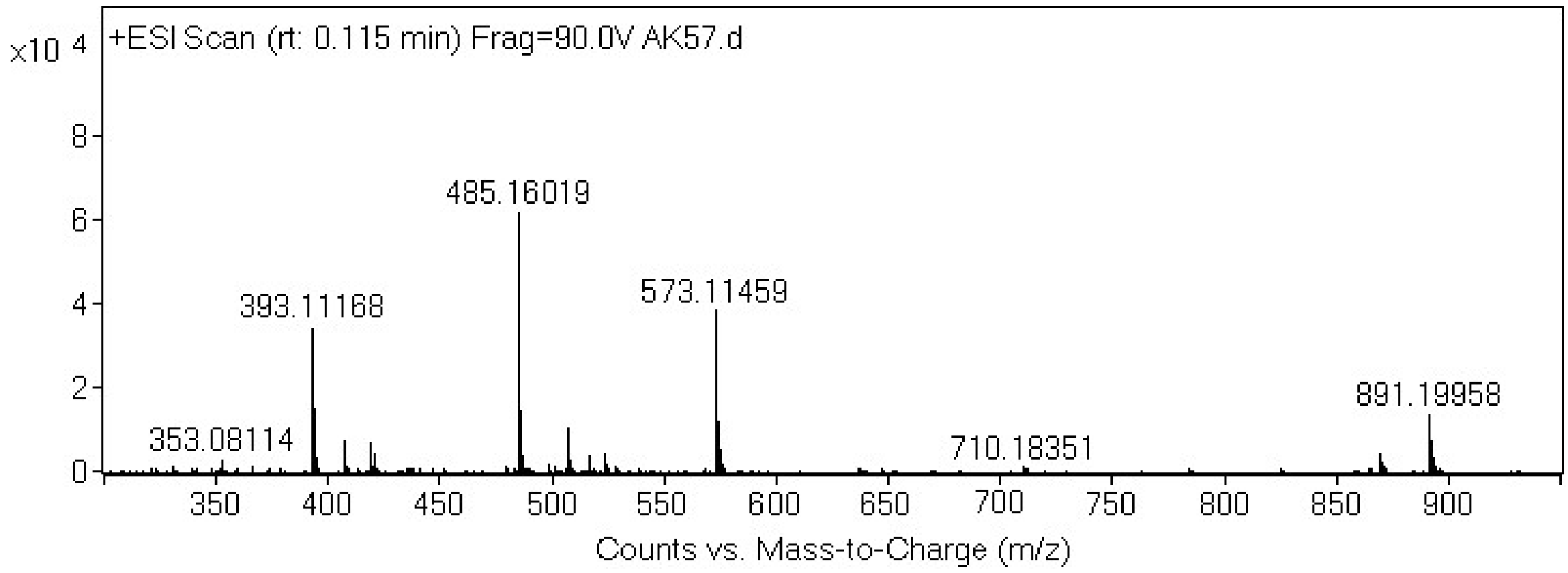

Figure S39. Mass spectrum of compound **4f**.

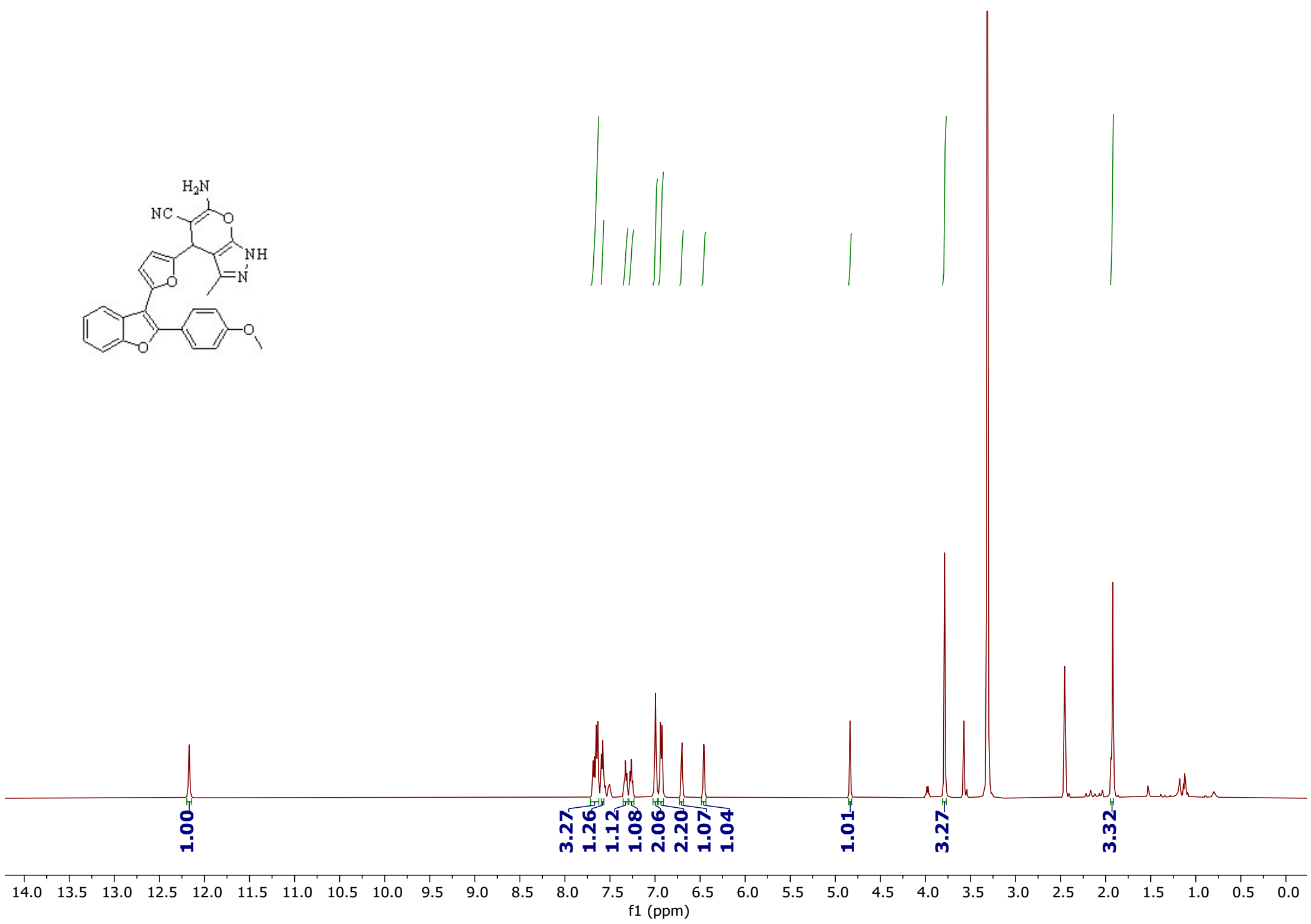

Figure S40. ^{1}H NMR spectrum of **4h**.

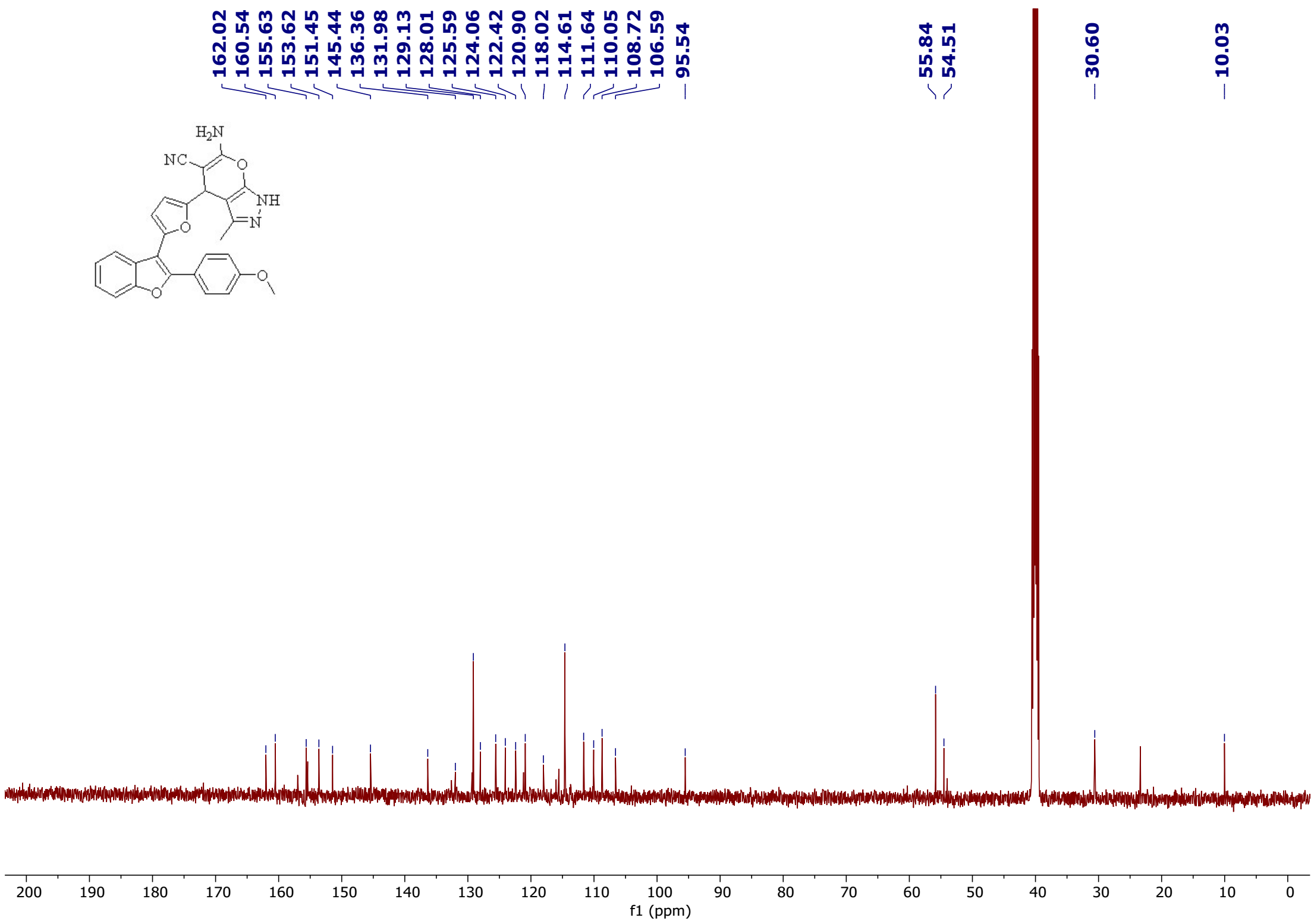

Figure S41. ^{13}C NMR spectrum of **4h**.

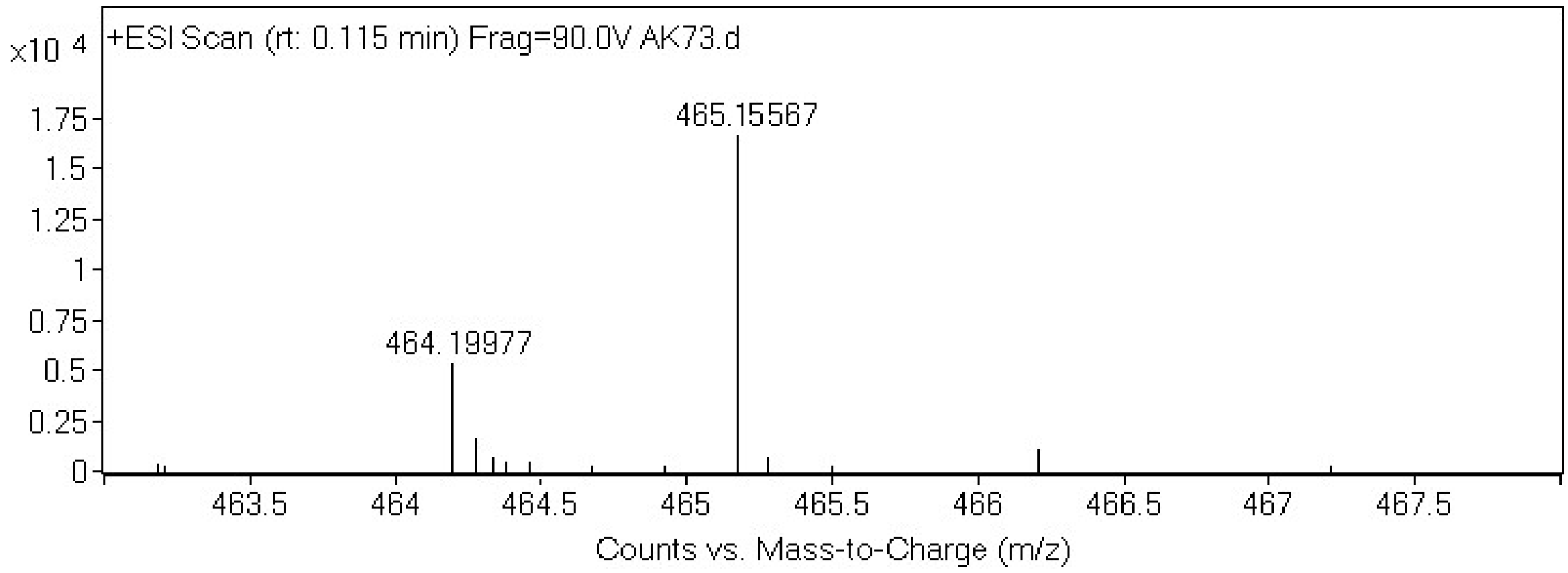

Figure S42. Mass spectrum of compound **4h**.

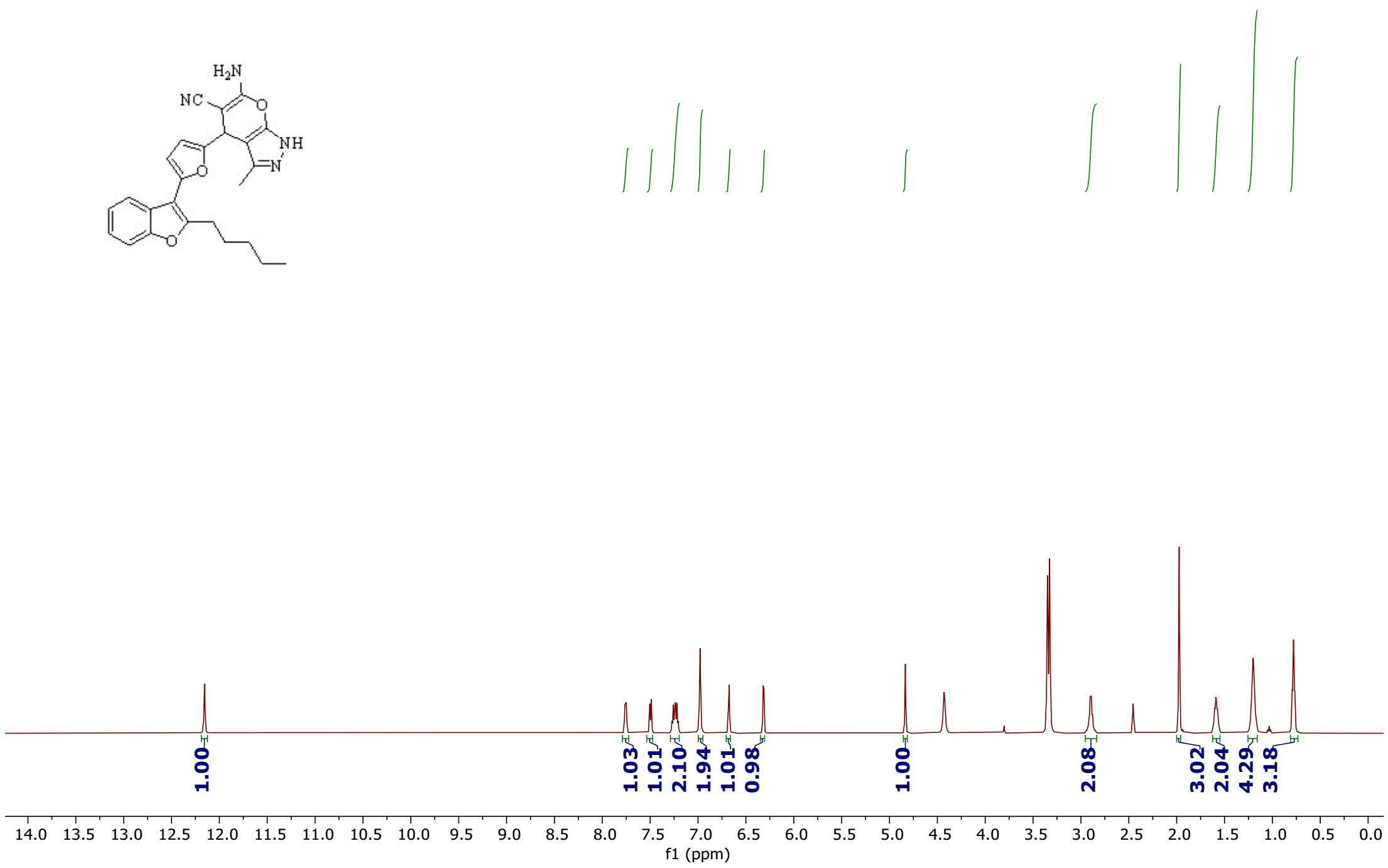

Figure S43. ^{1}H NMR spectrum of **4j**.

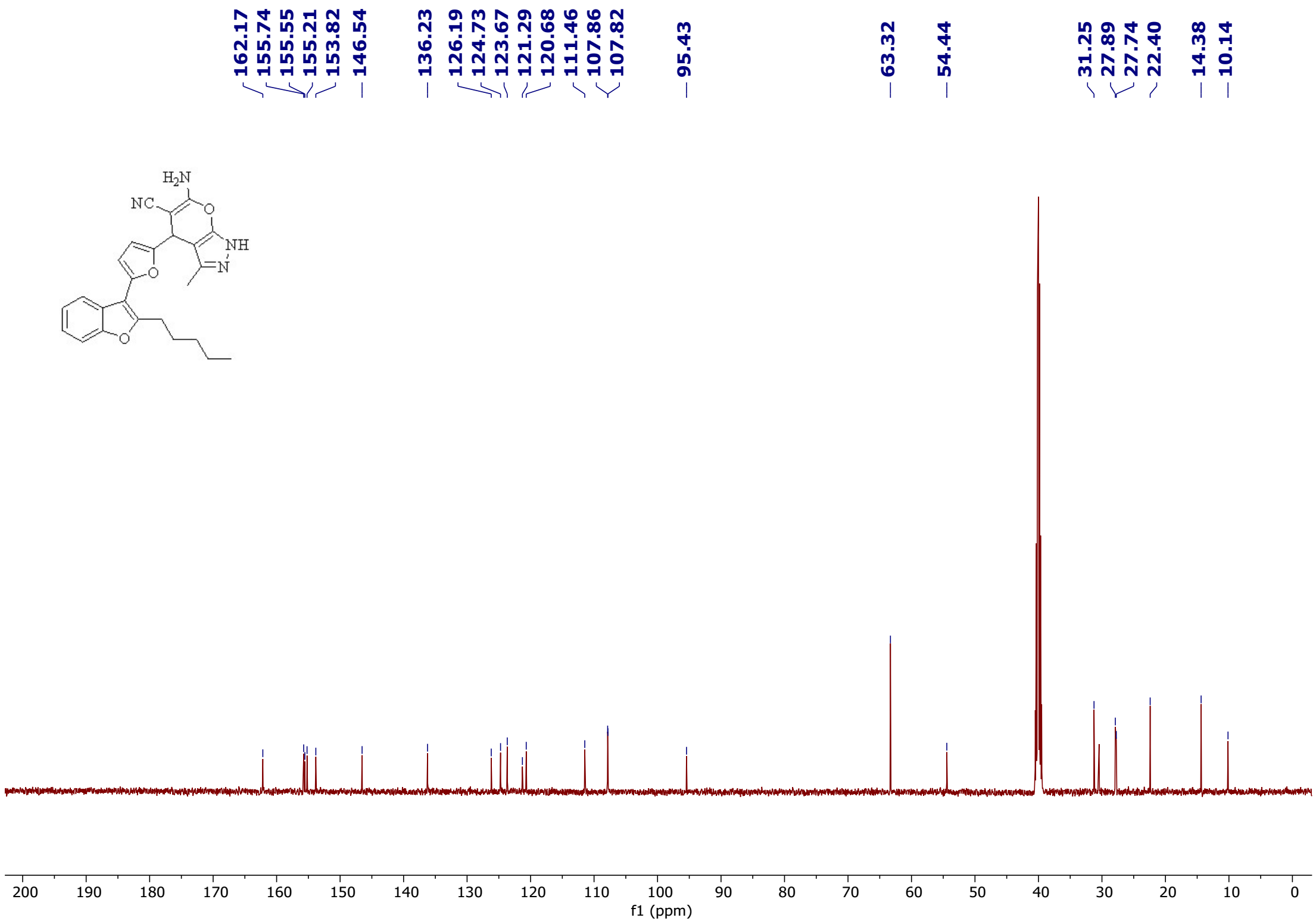

Figure S44. ^{13}C NMR spectrum of **4j**.

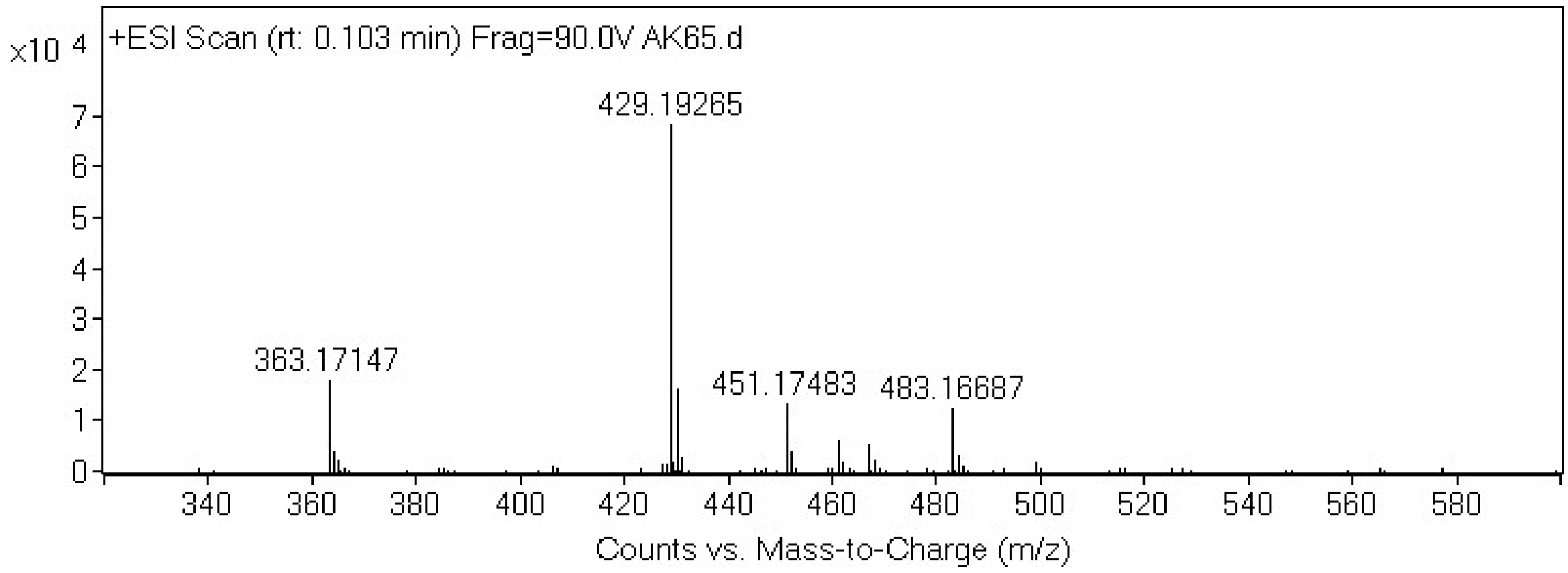

Figure S45. Mass spectrum of compound **4j**.

Complex		3D	2D
ATP	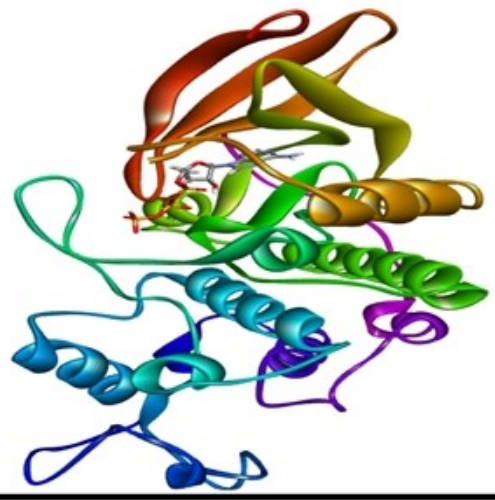	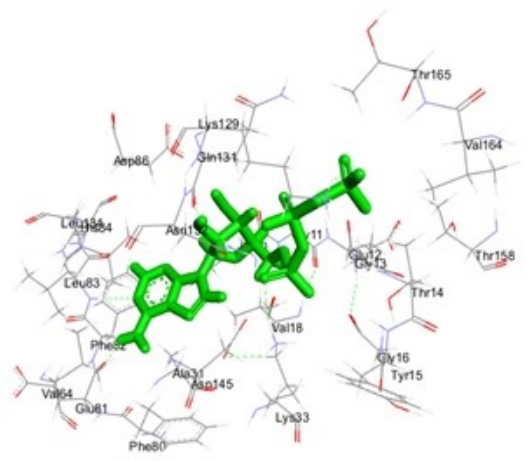	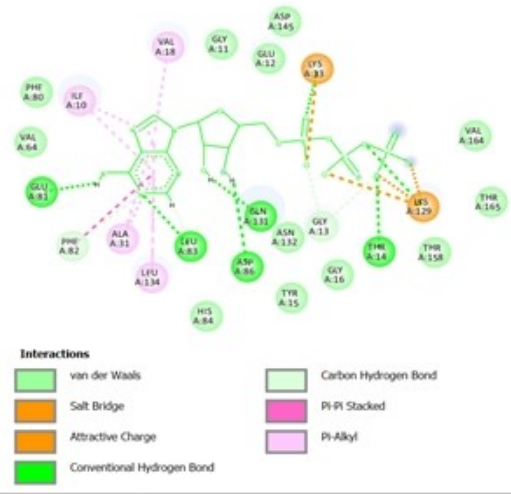
DHT	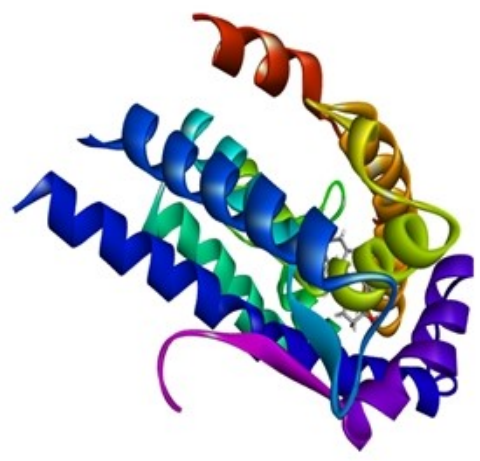	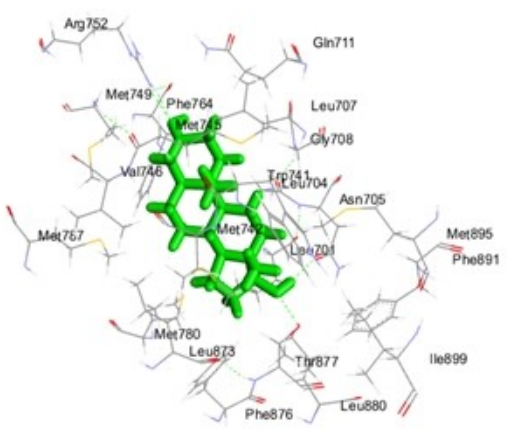	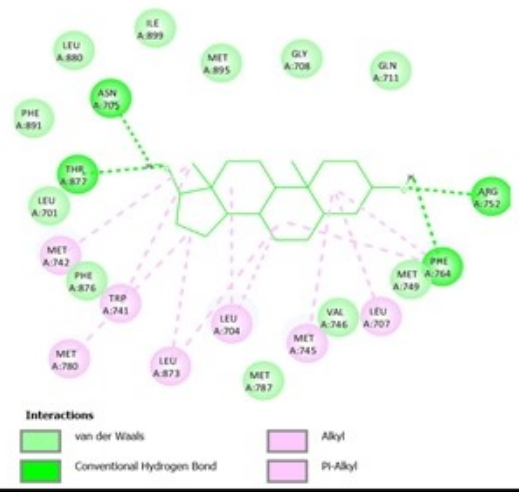
TIV	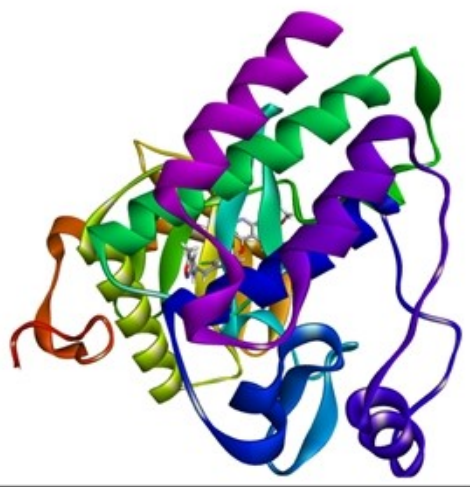	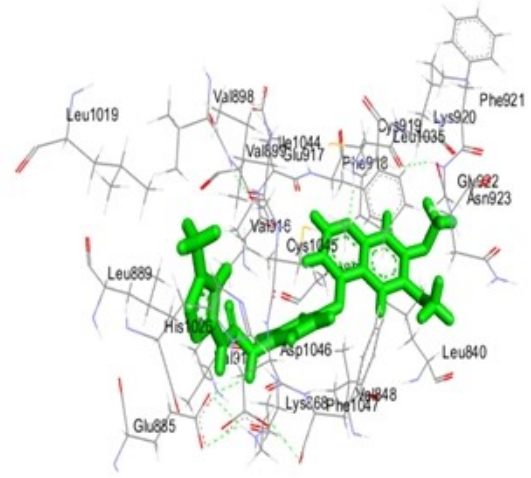	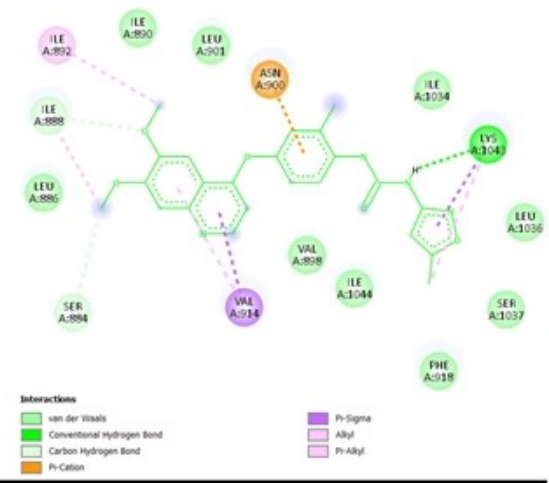
1E9	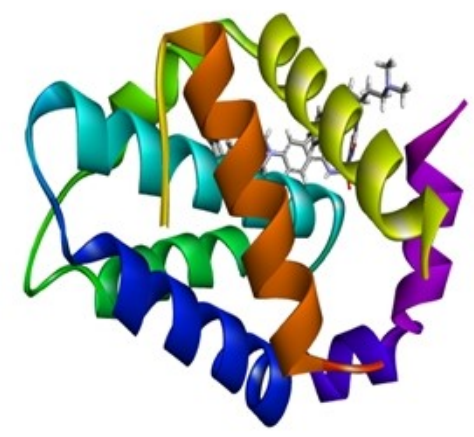	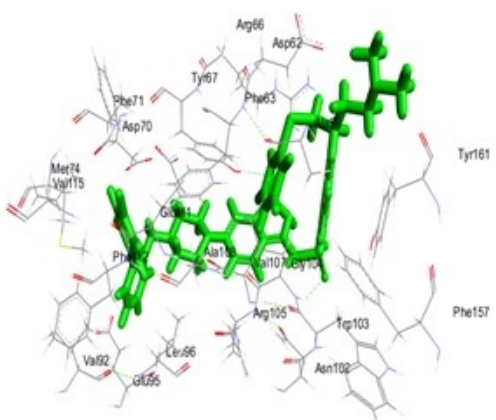	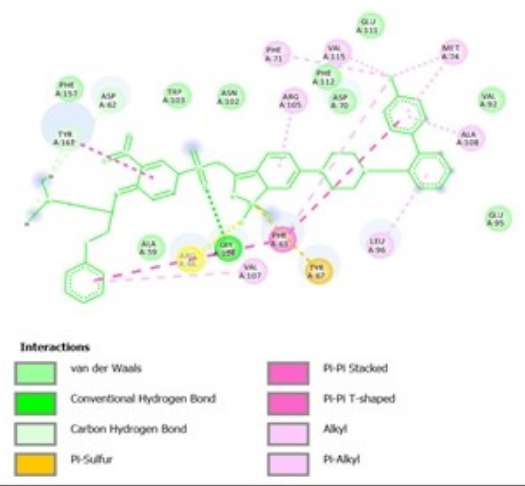

Figure S46. 3D and 2D interaction profiles of redocked ATP, DHT, TIV, and 1E9 in 1HCK, 2AMA, 4ASE, and 4IEH, respectively.

Table S1. Key conventional hydrogen-bonding and hydrophobic/π-related interactions of compounds **4b**, **4d**, **4f**, **4h**, and **4j**, together with the reference ligands SEL, ENZ, TIV, and VEN, within the binding pockets of CDK2 (1HCK), androgen receptor (2AMA), VEGFR-2 (4ASE), and Bcl-2 (4IEH).

Protein	Compound	Key conventional H-bond interactions	Key hydrophobic/π-related interactions
**1HCK**	**4b**	HIS125, LEU148	PHE152 (π-π), LEU148 (π-σ), ILE35, PRO155, ALA149 (π-alkyl)
	**4d**	HIS125	HIS125 (π-π), LEU148 (π-σ), ILE35, ARG36 (π-alkyl)
	**4f**	HIS125, LEU148	PHE152 (π-π), LEU148 (π-σ), ILE35, PRO155 (π-alkyl)
	**4h**	HIS125, LEU148, PHE146	ILE35, PHE80, LEU148 (π-σ), ARG36, ALA144 (π-alkyl)
	**4j**	HIS125, LEU148	HIS125 (π-π), ILE35, LEU148 (π-σ), ALA149 (alkyl/π-alkyl)
	**SEL**	PHE146	ALA149 (π-σ), LEU148 (amide-π stacked), ARG126, VAL154, PRO155, HIS125 (alkyl/π-alkyl)
**2AMA**	**4b**	TYR763	PHE770 (π-π), MET787 (π-σ), CYS784, LEU873, VAL746, LEU762, LEU768 (π-alkyl)
	**4d**	—	PHE876 (π-π), LEU701, LEU873, MET742, LEU880, ILE899, PHE891, LEU881, LEU707 (alkyl/π-alkyl)
	**4f**	LEU700	PHE770 (π-π), LEU701 (π-σ), LEU707, MET745, VAL746, LEU762 (π-alkyl); MET742/MET787 (π-sulfur)
	**4h**	LEU707, GLU706, GLY708, MET745	PHE770 (π-π), LEU707/MET742 (π-σ), LEU762, LEU768, LEU873, MET787 (π-alkyl)
	**4j**	LEU700, LEU701, LEU707, GLU706, GLY708	LEU707 (π–σ), LEU762, LEU768, PHE770 (π-alkyl); MET745 (π-sulfur)
	**ENZ**	LEU701, GLU706, SER703, GLY708, LEU707, GLY750, SER753	PHE770 (π-σ), LEU762, MET787 (π-alkyl), VAL746 (halogen interaction)
**4ASE**	**4b**	PHE918, GLY922, ILE1034, LEU1036, LYS1043	LYS1043 (π-alkyl)
	**4d**	ASN900, ARG1032, ASN1033, GLY922, LEU1036	VAL898 (π-σ), ILE1044 (amide-π), LYS1043, VAL865 (alkyl/π-alkyl)
	**4f**	ASN900, PHE918, VAL867	ILE915 (π-σ), LEU902, VAL865, LYS1043 (π-alkyl)
	**4h**	ASN900, LEU1036, VAL867, VAL914	LYS1043 (alkyl/π-alkyl)
	**4j**	ASN1033, ASN900, LEU1036, LYS1043	LYS1043 (π-σ/alkyl), LEU902 (π-alkyl)
	**TIV**	LYS1043	VAL914, LYS1043 (π-σ), ILE892 (π-alkyl), ASN900 (π-cation)
**4IEH**	**4b**	ARG68, SER64, ARG26	VAL115, VAL118 (π-σ), LYS22 (π-alkyl)
	**4d**	GLU119	VAL115 (π-σ), VAL118, LYS22, ALA72 (alkyl/π-alkyl)
	**4f**	GLU111	VAL115, VAL118 (π-σ), ALA72 (π-alkyl)
	**4h**	LYS22, VAL115, ALA72	VAL115 (π-σ), VAL118 (π-alkyl)
	**4j**	ARG26, ARG68	VAL115 (π-σ), VAL118, LYS22 (alkyl/π-alkyl)
	**VEN**	ARG68, ASP70	VAL115 (π-σ), GLY114 (amide-π stacked), VAL118, PHE112, LEU96 (alkyl/π-alkyl), ALA72 (π-cation)

Complex		3D	2D
**4b**	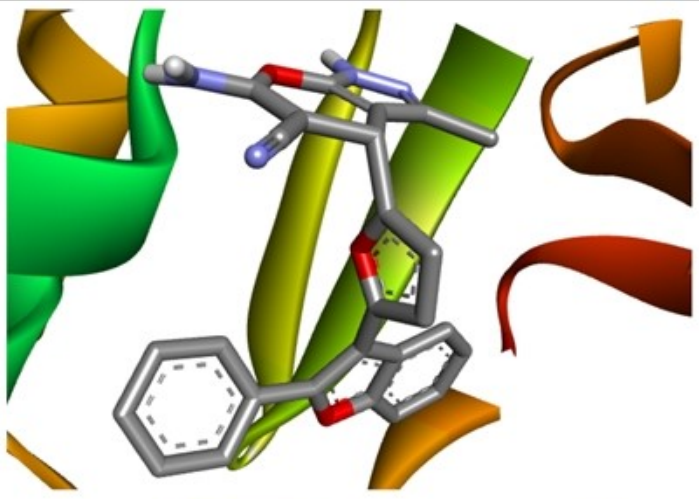	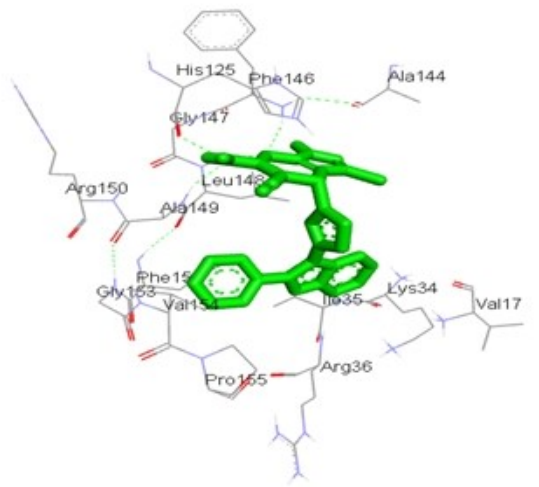	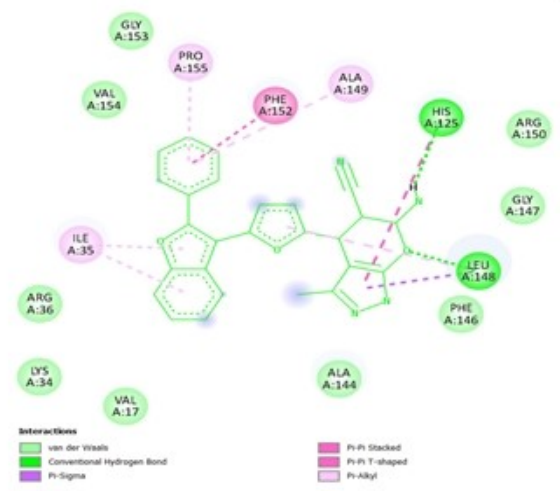
**4d**	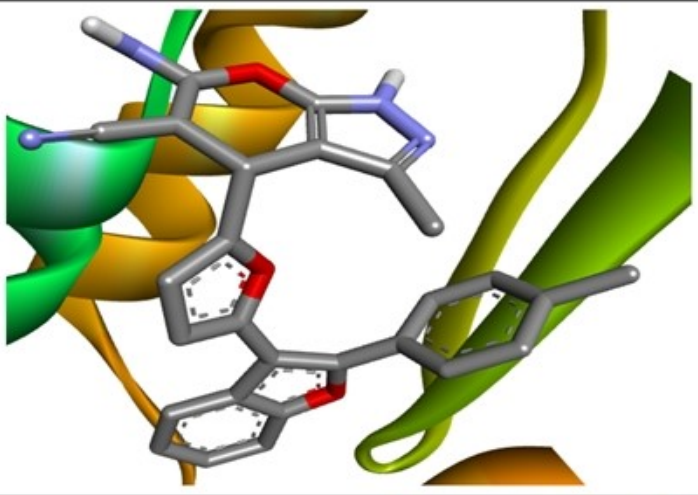	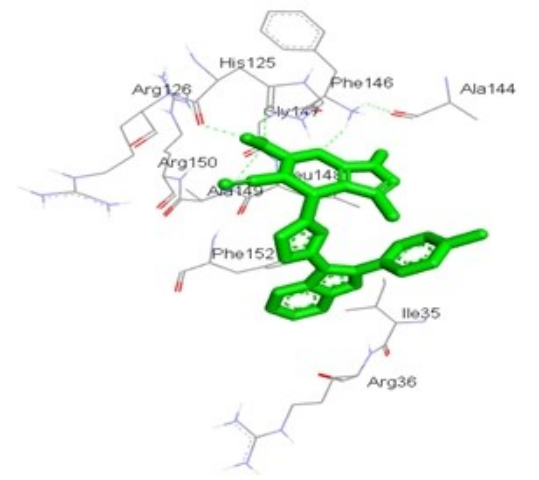	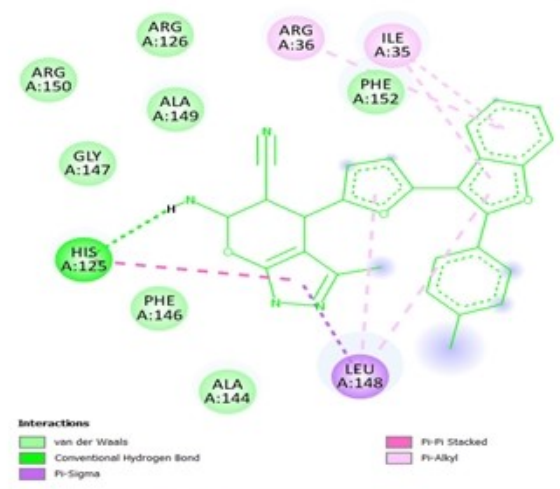
**4f**	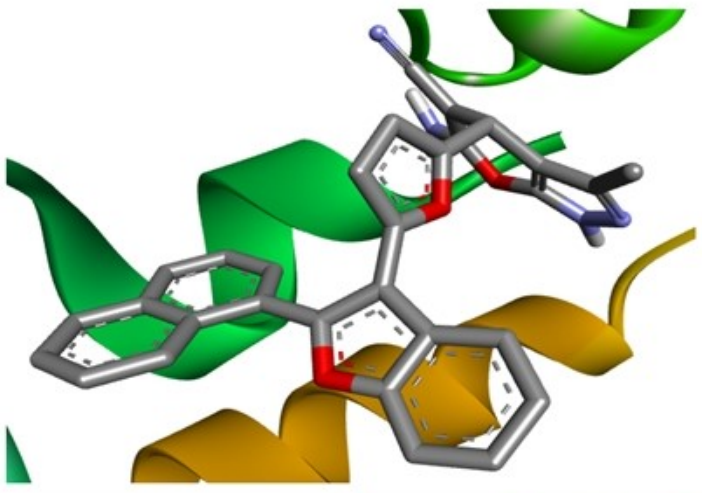	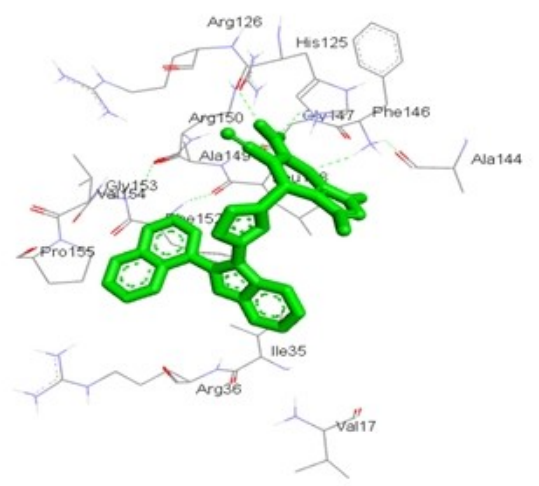	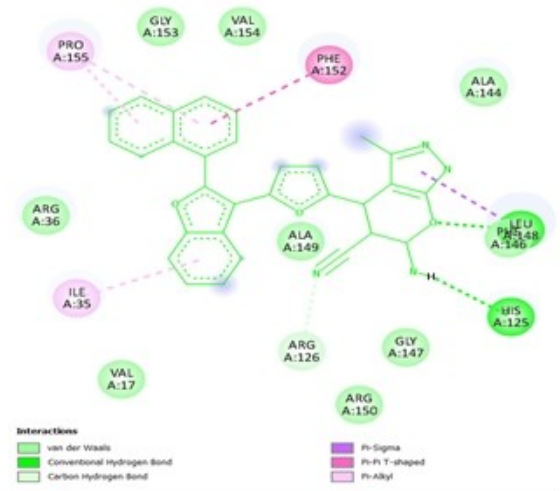
**4h**	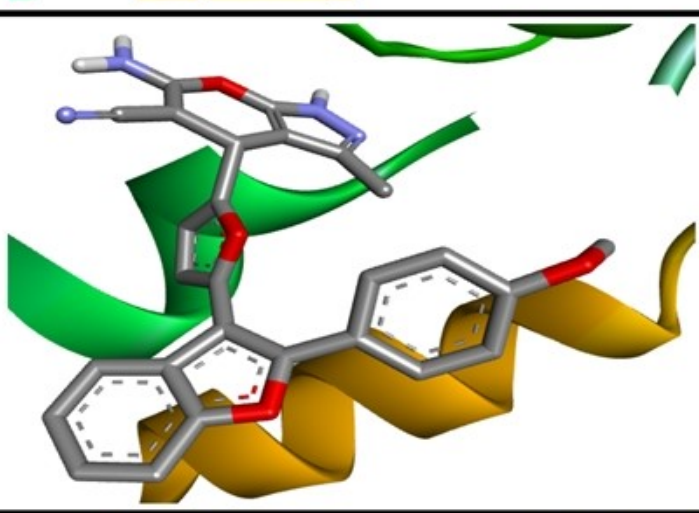	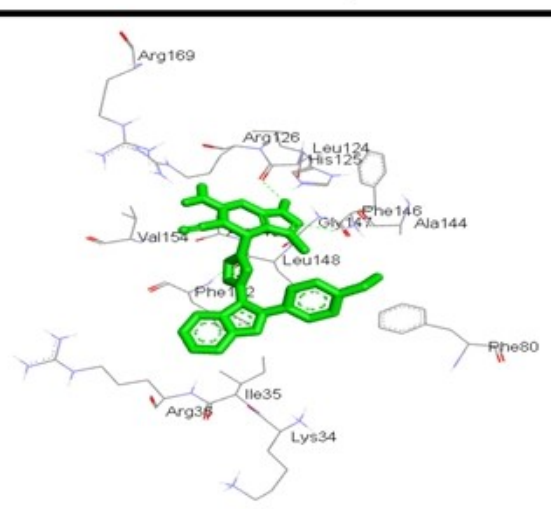	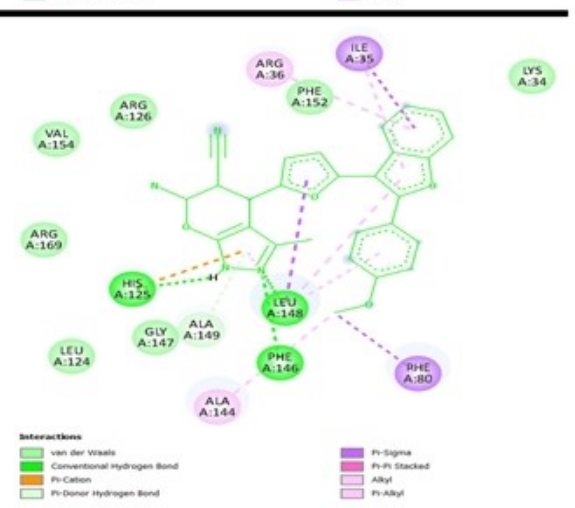
**4j**	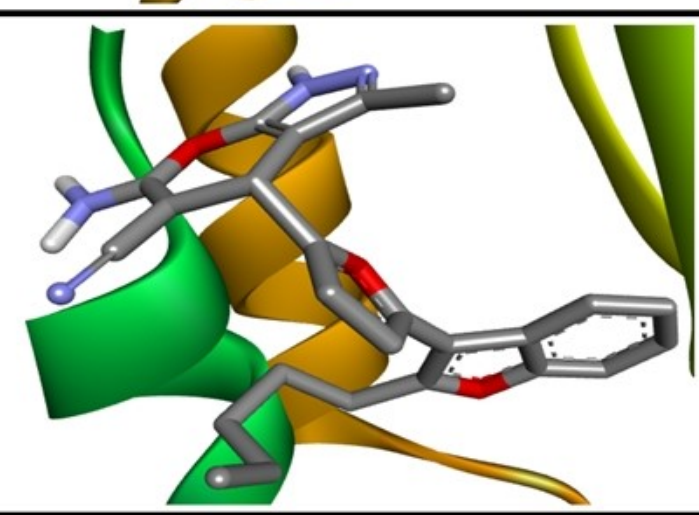	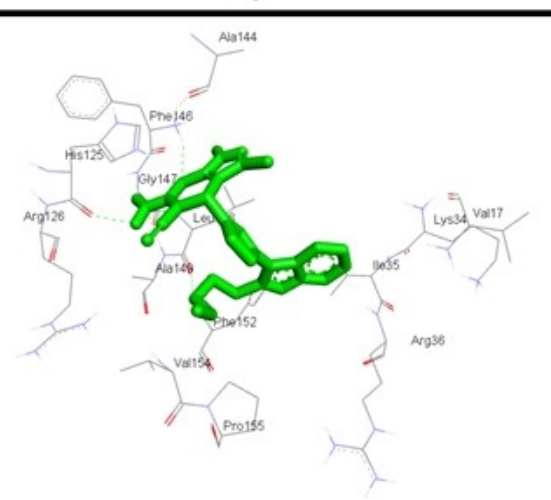	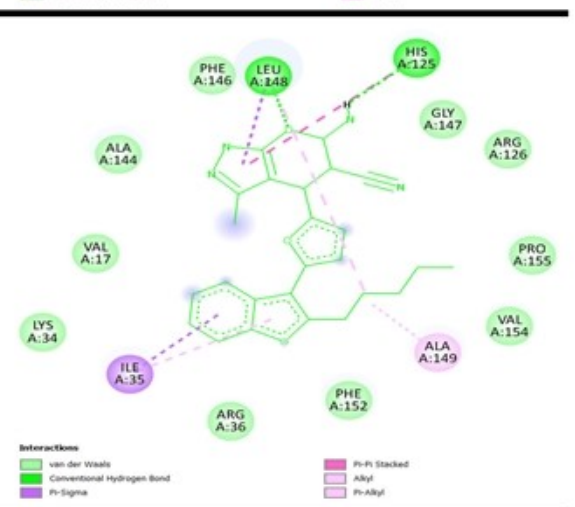
**SEL**	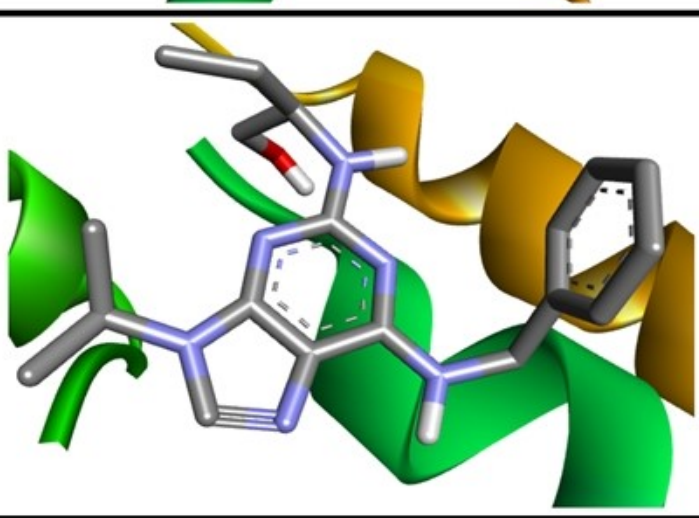	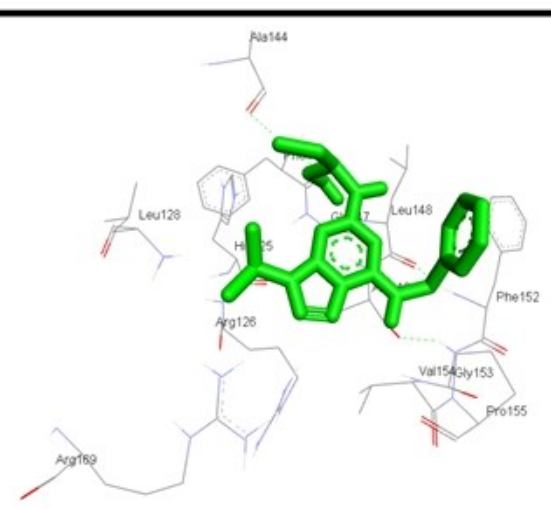	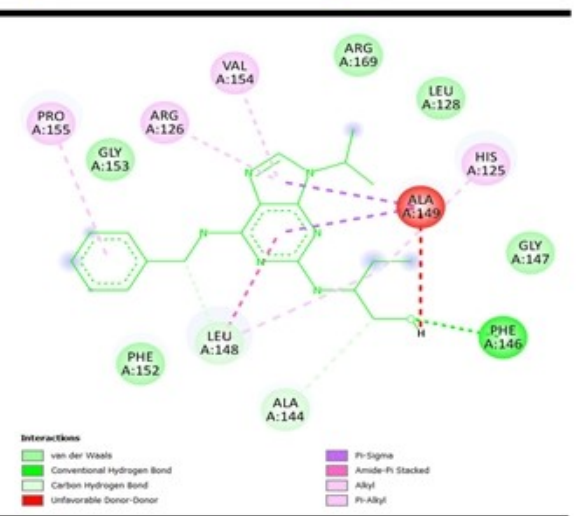

Figure S47. 3D and 2D docking interaction views of benzofuran-furan-pyrano[2,3-c]pyrazole hybrids (**4b, 4d, 4f, 4h, and 4j**) and the reference drug seliciclib (SEL) within the active site of 1HCK.

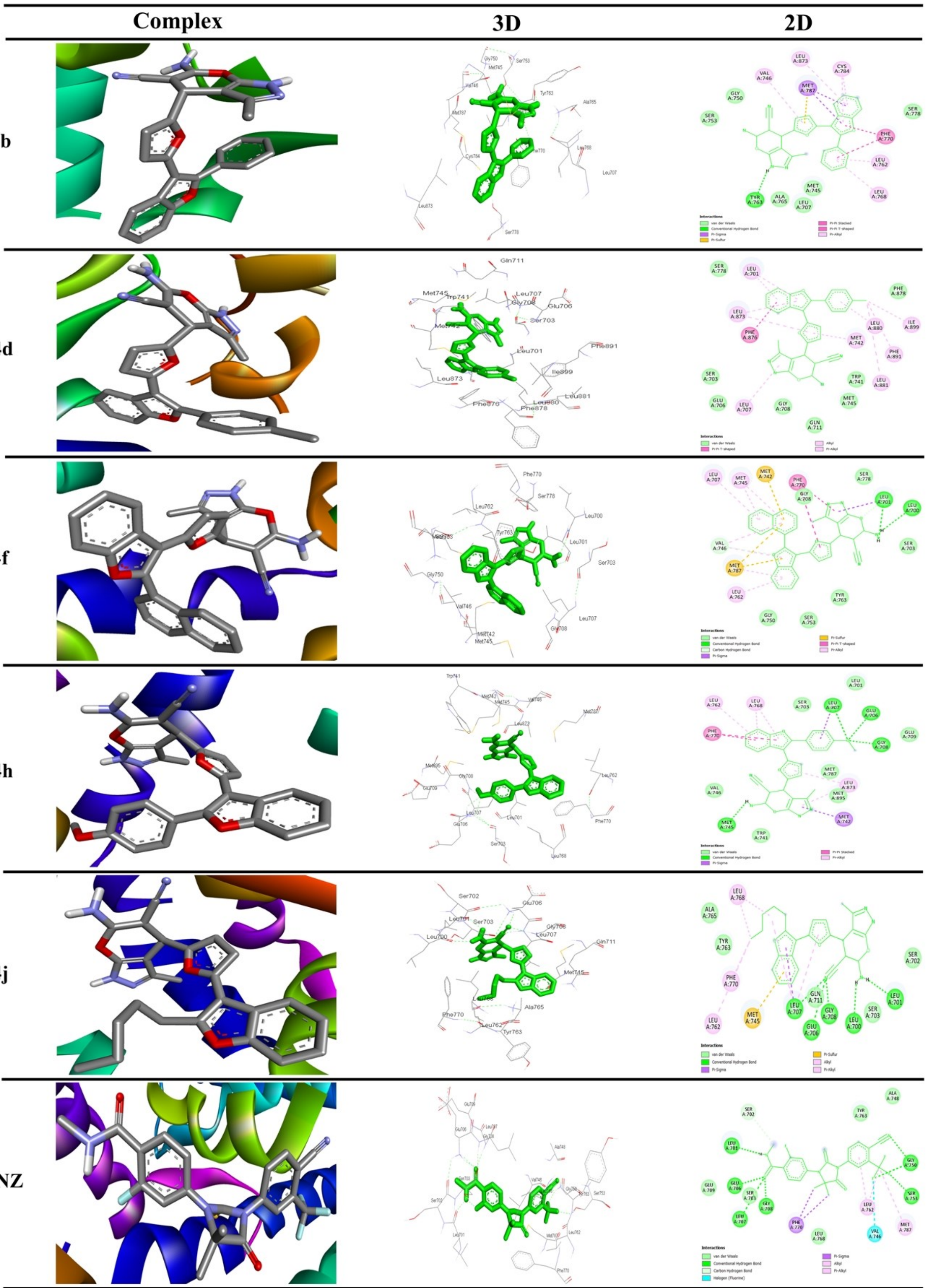

Figure S48. 3D and 2D docking interaction views of benzofuran-furan-pyrano[2,3-c]pyrazole hybrids (**4b, 4d, 4f, 4h, and 4j**) and the reference drug enzalutamide (ENZ) within the active site of 2AMA.

Complex		3D	2D
4b	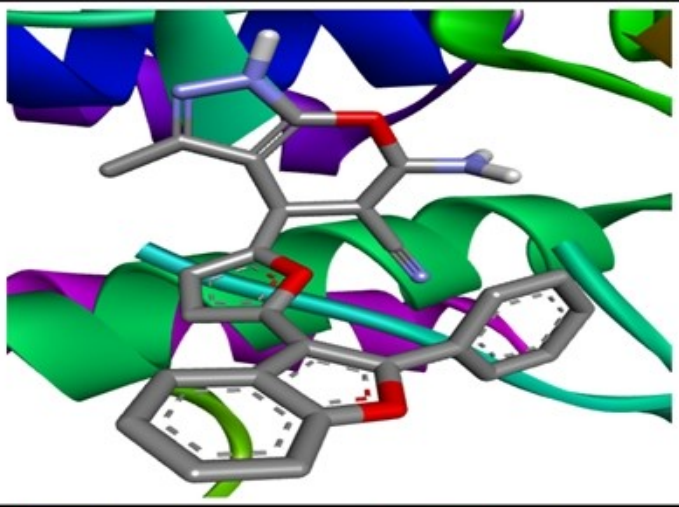	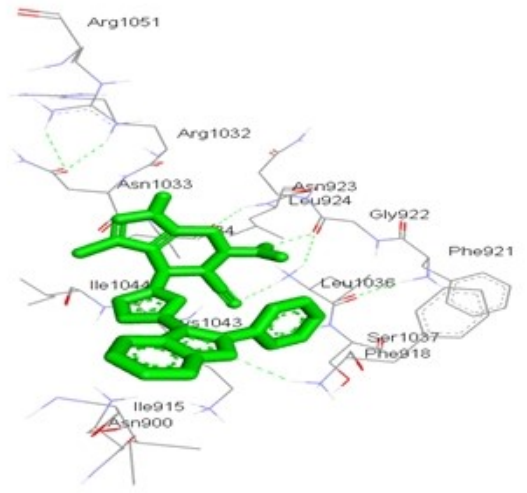	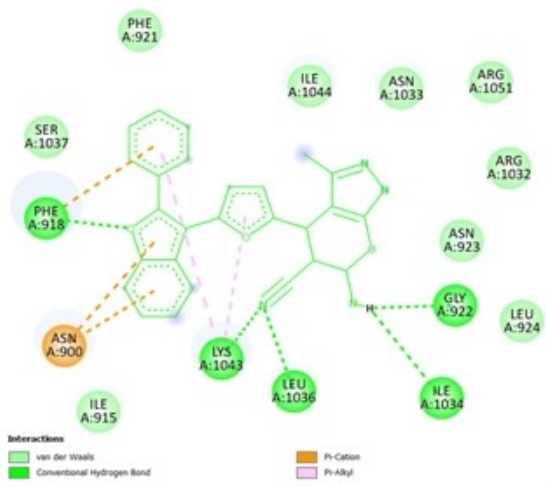
4d	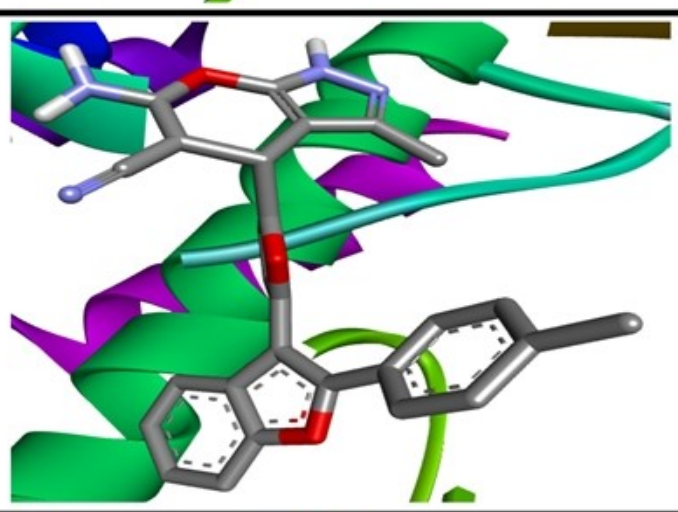	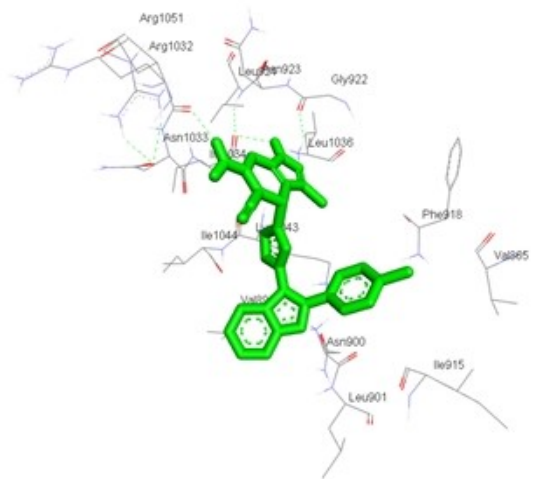	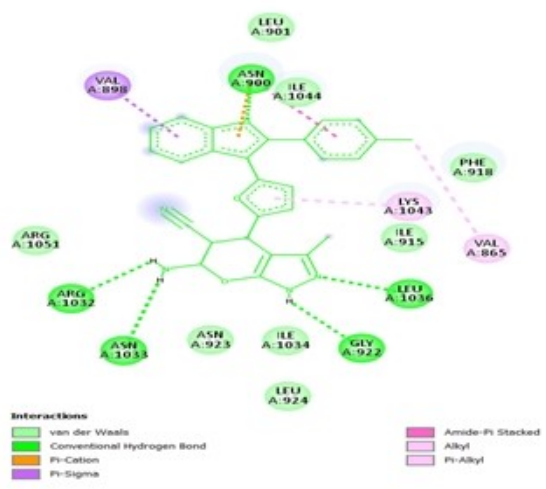
4f	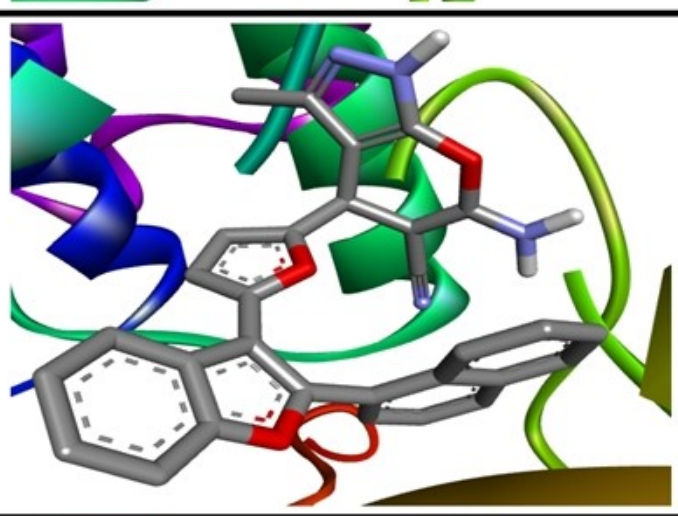	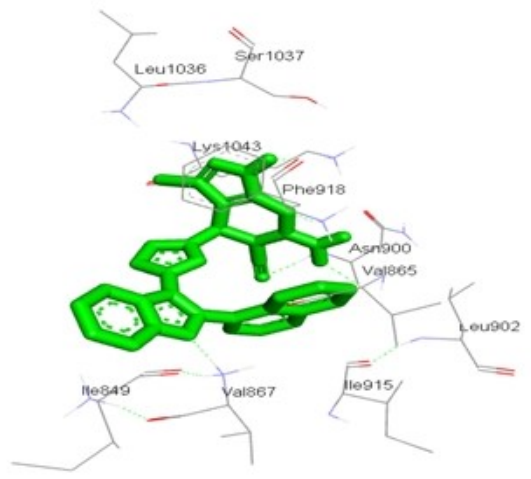	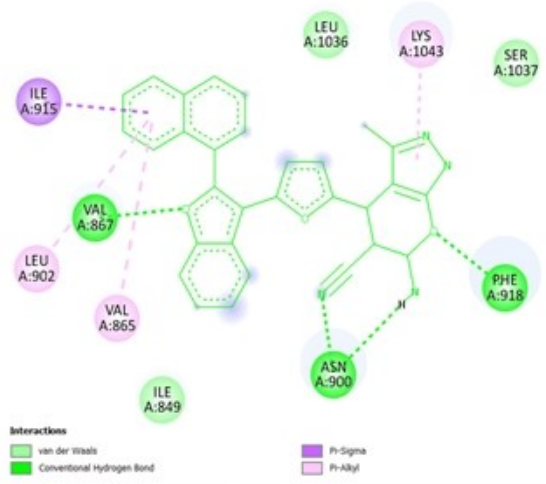
4h	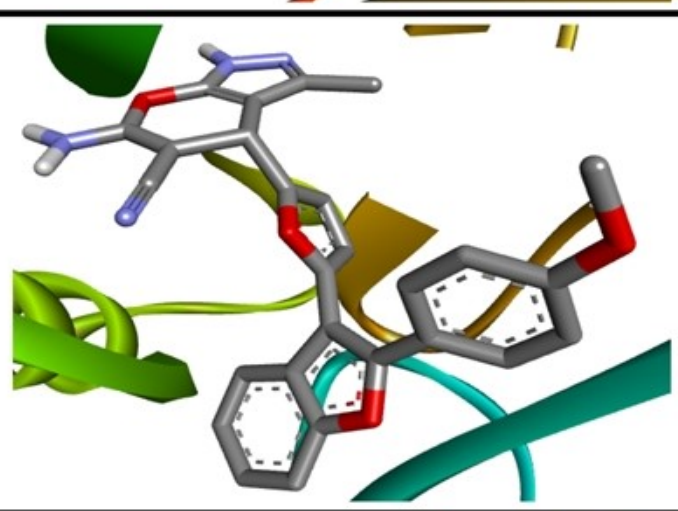	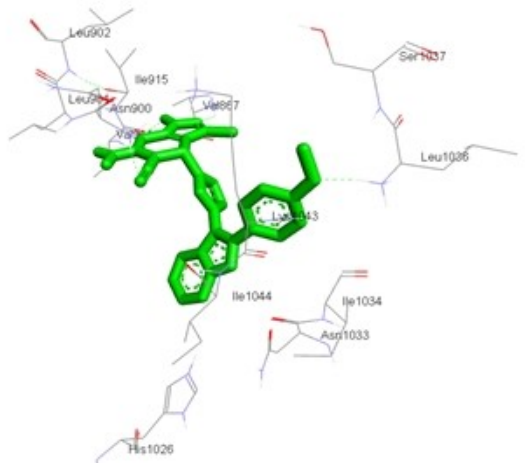	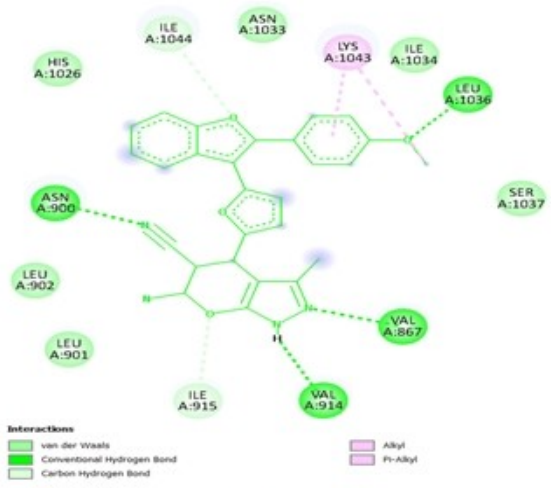
4j	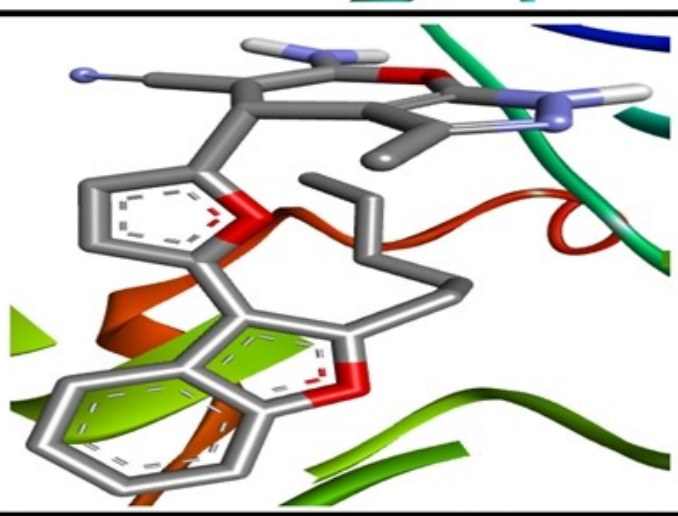	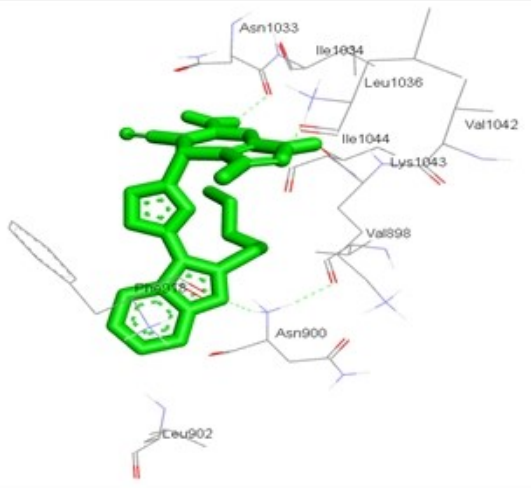	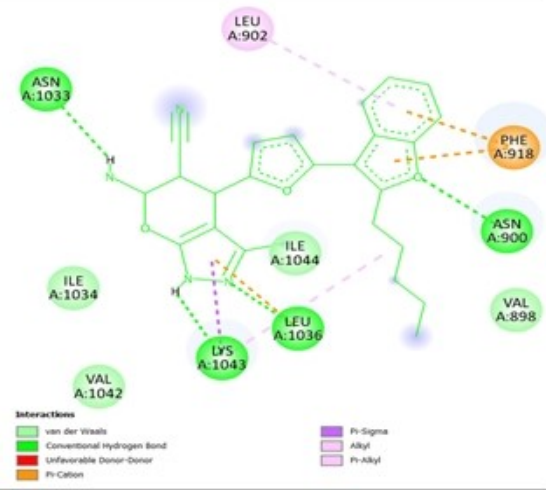
TIV	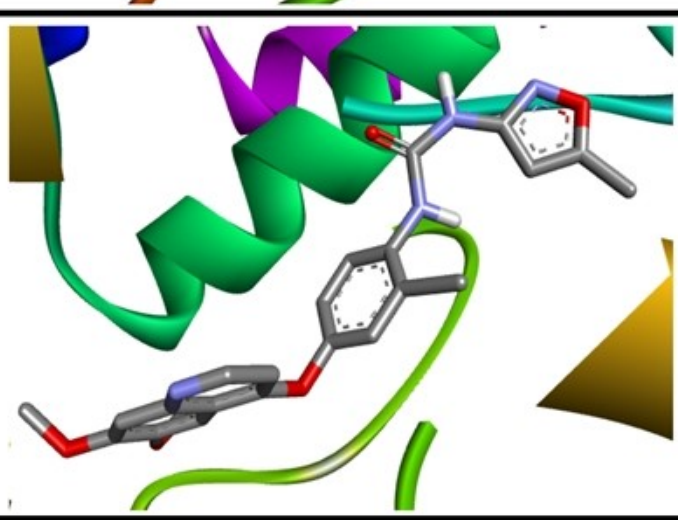	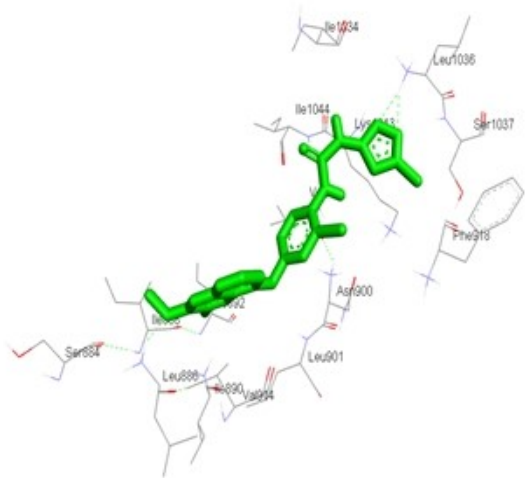	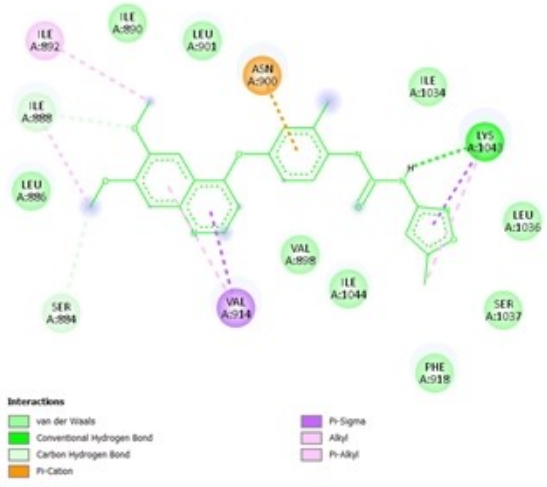

Figure S49. 3D and 2D docking interaction views of benzofuran-furan-pyrano[2,3-c]pyrazole hybrids (**4b, 4d, 4f, 4h, and 4j**) and the reference drug tivozanib (TIV) within the active site of 4ASE.

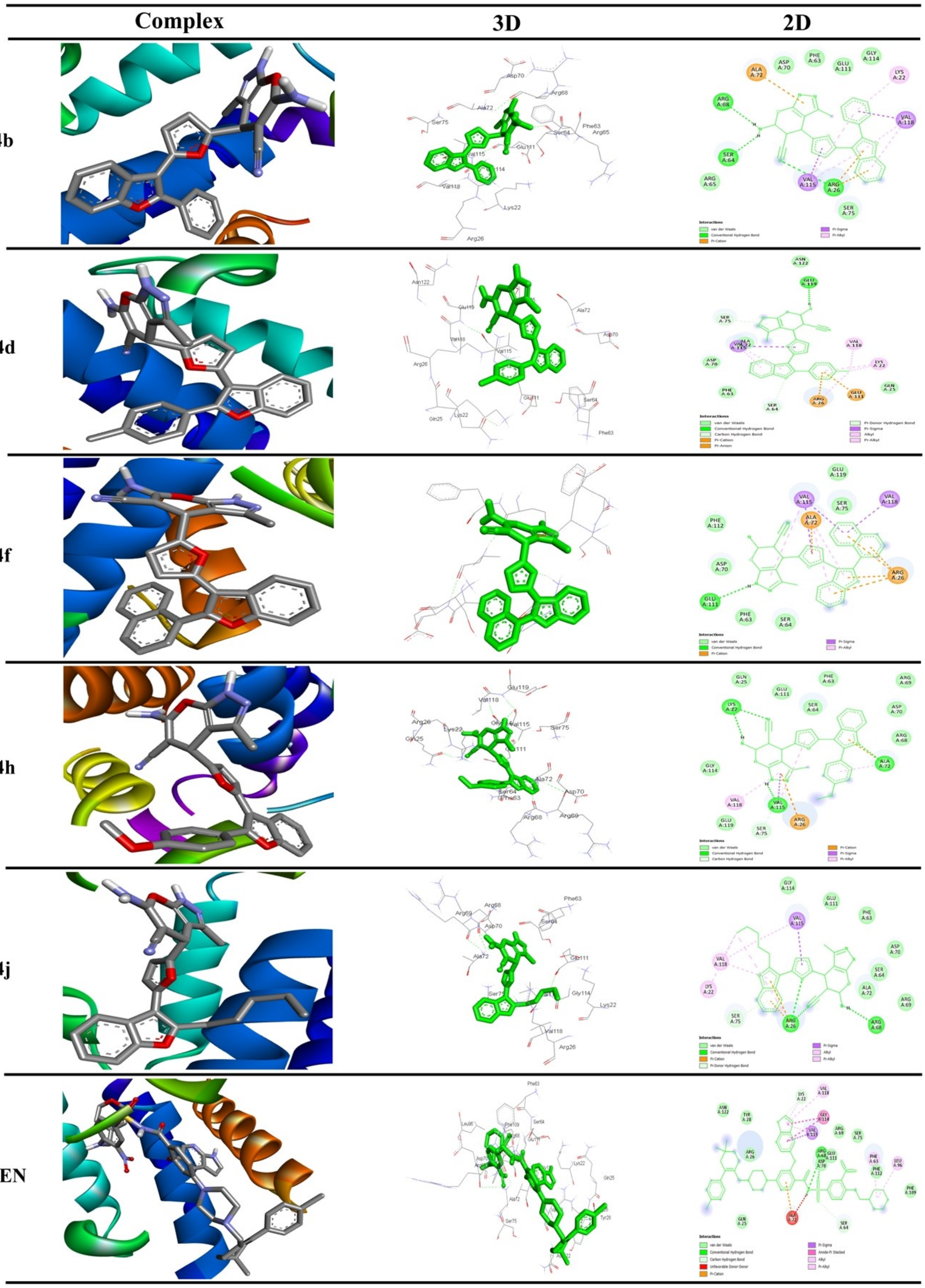

Figure S50. 3D and 2D docking interaction views of benzofuran-furan-pyrano[2,3-c]pyrazole hybrids (**4b, 4d, 4f, 4h, and 4j**) and the reference drug venetoclax (VEN) within the active site of 4IEH.
